# Chromenol Derivatives as Novel Antifungal Agents: Synthesis, In Silico and In Vitro Evaluation

**DOI:** 10.3390/molecules26144304

**Published:** 2021-07-16

**Authors:** Marina Zveaghintseva, Eugenia Stingaci, Serghei Pogrebnoi, Anastasia Smetanscaia, Vladimir Valica, Livia Uncu, Victor Ch. Kravtsov, Elena Melnic, Anthi Petrou, Jasmina Glamočlija, Marina Soković, Alejandro Carazo, Přemysl Mladěnka, Vladimir Poroikov, Athina Geronikaki, Fliur Z. Macaev

**Affiliations:** 1 Laboratory of Organic Synthesis, Institute of Chemistry, 3 Str. Academiei 3, MD-2028 Chișinău, Moldova; mari6azv@mail.ru (M.Z.); stingacieugenia@gmail.com (E.S.); richserg@gmail.com (S.P.); 2 Scientific Center for Drug Research, “Nicolae Testemițanu” State University of Medicine and Pharmacy, Bd. Stefan Cel Mare și Sfant 165, MD-2004 Chișinău, Moldova; anastasia.smetanscaia@gmail.com (A.S.); vvalica@usmf.md (V.V.); livia.uncu@usmf.md (L.U.); 3 Laboratory of Physical Methods of Solid State Investigation “Tadeusz Malinowski”, Institute of Applied Physics, Str. Academiei 5, MD-2028 Chișinău, Moldova; kravtsov.xray@gmail.com (V.C.K.); elena.melnic2@gmail.com (E.M.); 4 Department of Pharmacy, School of Health, Aristotle University of Thessaloniki, 54124 Thessaloniki, Greece; anthi.petrou.thessaloniki1@gmail.com; 5 Mycological Laboratory, Department of Plant Physiology, Institute for Biological Research “Siniša Stanković”, National Institute of Republic of Serbia, University of Belgrade, 11060 Beograd, Serbia; jasna@ibiss.bg.ac.rs (J.G.); mris@ibiss.bg.ac.rs (M.S.); 6 Department of Pharmacology and Toxicology, Faculty of Pharmacy, Charles University, 500 05 Hradec Králové, Czech Republic; carazofa@faf.cuni.cz (A.C.); mladenkap@faf.cuni.cz (P.M.); 7 Laboratory of Structure-Function Based Drug Design, Institute of Biomedical Chemistry, Pogodinskaya Str. 10, Bldg. 8, 119121 Moscow, Russia; vladimir.poroikov@ibmc.msk.ru

**Keywords:** vinyl-1,2,4-triazole, chromenol, antifungal activity, *C. albicans* CYP51, PASS, molecular docking

## Abstract

Herein we report the synthesis of some new 1*H*-1,2,4-triazole functionalized chromenols (**3a**–**3n**) via tandem reactions of 1-(alkyl/aryl)-2-(1*H*-1,2,4-triazole-1-yl) with salicylic aldehydes and the evaluation of their antifungal activity. In silico prediction of biological activity with computer program PASS indicate that the compounds have a high novelty compared to the known antifungal agents. We did not find any close analog among the over 580,000 pharmaceutical agents in the Cortellis Drug Discovery Intelligence database at the similarity cutoff of 70%. The evaluation of antifungal activity in vitro revealed that the highest activity was exhibited by compound **3k**, followed by **3n**. Their MIC values for different fungi were 22.1–184.2 and 71.3–199.8 µM, respectively. Twelve from fourteen tested compounds were more active than the reference drugs ketoconazole and bifonazole. The most sensitive fungus appeared to be *Trichoderma viride*, while *Aspergillus fumigatus* was the most resistant one. It was found that the presence of the 2-(*tert*-butyl)-2*H*-chromen-2-ol substituent on the 4th position of the triazole ring is very beneficial for antifungal activity. Molecular docking studies on *C. albicans* sterol 14α-demethylase (CYP51) and DNA topoisomerase IV were used to predict the mechanism of antifungal activities. According to the docking results, the inhibition of CYP51 is a putative mechanism of antifungal activity of the novel chromenol derivatives. We also showed that most active compounds have a low cytotoxicity, which allows us to consider them promising antifungal agents for the subsequent testing activity in in vivo assays.

## 1. Introduction

**Figure 1:**
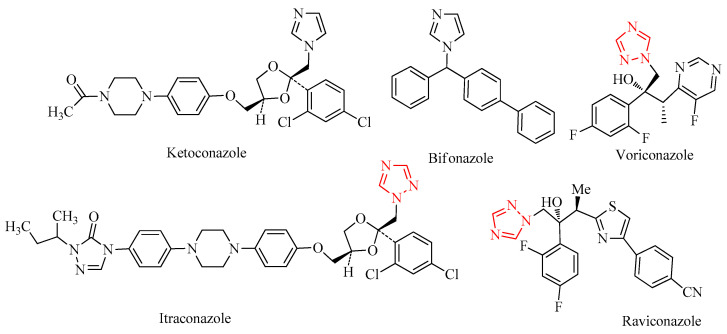
Selected pharmaceutically relevant azoles derivatives and 1,2,4-triazoles emphasized by a red color.

Azoles constitute a significant class of antifungal drugs frequently used to treat infections caused by many pathogenic fungi. Azole antifungals are pretty inexpensive, have similar chemical structures, and are effective against most fungal species. Azoles target a crucial enzyme in the ergosterol biosynthesis pathway, CYP51, whose inhibition leads to a reduced fungal growth [1,2]. Ketoconazole [3,4], itraconazole [3,5], bifonazole [3,6], ravuconazole [3,7], and voriconazole [3,8,9] (Figure 1) are examples of drugs from this class [3]. Since azoles are fungistatic instead of fungicidal, their prolonged use and abuse frequently results in resistance, which is a severe clinical problem in antifungal therapy. The gradual increase in systemic fungal infections over the last three decades has led to high morbidity and mortality rates due to the unavailability of effective medications. The development of resistant strains contributed markedly to this phenomenon. The most aggressive fungal infections occur due to immune suppression associated with several illnesses, such as AIDS, and several drugs which are applied for cancer chemotherapy, immunosuppressive therapy for organ transplantation, and corticosteroid therapy in inflammatory diseases. More than 90% of reported fungal-associated deaths result from species belonging to three genera: Cryptococcus, Candida, and Aspergillus [10]. Clinically, candidiasis and aspergillosis represent 80% to 90% of systemic fungal infections in immunocompromised patients.

Extensive use and long-term therapy with azoles resulted in fungal resistance [11,12]. Most of the drugs used in antifungal treatment, except azoles and polyenes, are characterized by low potency, a narrow spectrum of activity, and many severe side effects. These precipitate an urgent need to develop novel effective antifungal azoles with a better clinical efficacy and low risk of side effects. One way to overcome this rapid development of drug resistance is to design new agents with chemical characteristics that are different from those of existing agents. Especially the mechanism of action should be ultimately the same, e.g., different binding sites or different targets are the best possibilities for avoiding cross-resistance to existing therapeutics [13].

Triazole derivatives have attracted considerable interest in the scientific community due to their vast range of biological activities. In addition to antifungal action [14,15,16,17,18,19], they were shown to possess other antimicrobial effects such as antibacterial, including anti-tuberculous activity [20,21,22,23,24], antiparasitic [25,26,27] and anti-HIV effects [28] as well as anticholinesterase [29], antiangiogenic [30], anticancer [31,32], antidiabetic [33,34] and anticonvulsant activities [35].

Another interesting structural core is the chromen ring. Chromen derivatives were also reported to display a wide range of biological activities such as antioxidant [36,37], anti-inflammatory [38,39], antimicrobial [40,41,42,43,44,45], anti-HIV [46,47] and others [48,49].

These findings focused particular interest on incorporating a triazole with a chromen ring in one frame to obtain compounds with improved antifungal activity. According to the current literature, more efficacious antibacterial compounds have been designed by joining two or more biologically active heterocyclic nuclei in a single molecular framework [50,51,52,53].

Taking all these into account, herein we report the design, synthesis, and evaluation of the antifungal activity of fourteen new chromenol–triazole hybrids.

## 2. Results and Discussion

### 2.1. Chemistry

**Figure 2:**
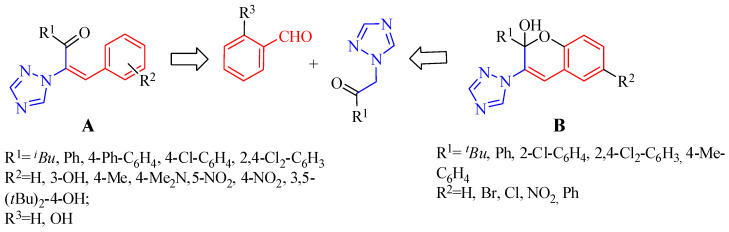
Structure of vinyl-1,2,4-triazole derivatives (**A**) and target chromene–triazole hybrid (**B**).

The synthesis of new vinyl-1,2,4-triazole derivatives **A** (Figure 1) as antimicrobial agents was previously reported [54]. Recent attention has only been paid to the tandem reactions of salicylic aldehydes or salicylic imines with α,β-unsaturated compounds [55]. We considered that an applicable route to the synthesis of 1*H*-1,2,4-triazole-functionalized chromene **B** (Figure 2) would be possible through the coupling of substituted triazolyl ethanones with salicylic aldehydes.

**Figure 3:**
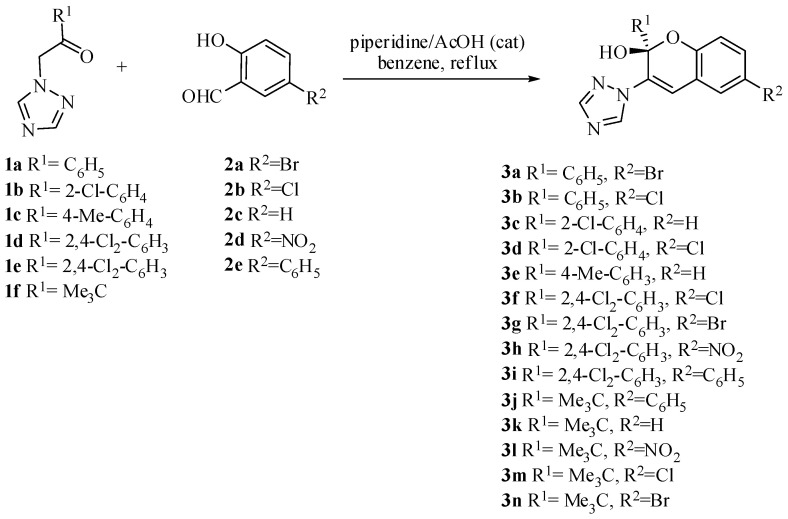
Synthesis of 1*H*-1,2,4-triazole functionalized chromenols.

Indeed, we performed the first synthesis of 1*H*-1,2,4-triazole-functionalized chromenols via tandem reactions of 1-(alkyl/aryl)-2-(1*H*-1,2,4-triazole-1-yl) with salicylic aldehydes (Figure 3).

The reaction of 1-(alkyl/aryl)-2-(1*H*-1,2,4-triazole-1-yl)ethanones **1a**–**1f** with salicylic aldehydes **2a**–**2e** in the presence of piperidine and acetic acid as catalyst-afforded crystalline products **3a**–**3n** with varying yields ranging from 25% up to 75%.

The structure of the obtained derivatives was supported by IR, ^1^H and ^13^C NMR spectroscopic data and by elemental analysis (see Supplementary Materials).

The IR spectrum of products showed an absorption band for both **3a** and **3b** at 880 cm^−1^, for **3c** at 877 cm^−1^, for **3d** at 894 cm^−1^, for **3e** at 896 cm^−1^, for all three **3f, 3g, 3h** at 825 cm^−1^, for **3i** at 874 cm^−1^, for **3j** at 891 cm^−1^, for **3k** at 875 cm^−1^, for **3l** at 883 cm^−1^, for **3m** at 873 cm^−1^ and for **3n** at 831 cm^−1^, characteristic of the cyclic vinyl C-H stretching frequency. The absorption band for **3a** at 1576 cm^−1^, for both **3c** and **3b** at 1573 cm^−1^, for **3d** at 1571 cm^−1^, for **3e** at 1578 cm^−1^, for both **3f** and **3h** at 1584 cm^−1^, for **3g** at 1560 cm^−1^, for **3i** at 1509 cm^−1^, for both **3j** and **3k** at 1508 cm^−1^, for **3l** at 1503 cm^−1^, for **3m** at 1505 cm^−1^ and for **3n** at 1502 cm^−1^, suggests the presence of a C–N bond. The absorption band for both **3a** and **3b** at 1140 cm^−1^, for **3c** at 1131cm^−1^, for **3d** at 1104 cm^−1^, for **3e** at 1111 cm^−1^, for **3f** at 1129 cm^−1^, for **3g** at 1126 cm^−1^, for **3h** at 1129 cm^−1^, for **3i** at 1147 cm^−1^, for **3j** at 1113 cm^−1^, for **3k** at 1125 cm^−1^, for **3l** at 1131 cm^−1^, for **3m** at 118 cm^−1^ and for **3n** at 1130 cm^−1^, was assigned to a hemiketal group of compounds **3a**–**n**. 

The examination of the NMR spectra of the discussed compounds further confirmed the formation of 1*H*-1,2,4-triazole functionalized chromenols by the intramolecular addition of a hydroxy group to a ketonic carbonyl group. The peaks in the ^13^C NMR spectra at 118.4 ppm for **3a**, 95.01 ppm for **3b**, 95.9 ppm for **3c**, 96.13 ppm for **3d**, 97.9 ppm for **3e**, 95.81 ppm for **3f**, 95.74 ppm for **3g**, 96.8 ppm for **3h**, 95.9 ppm for **3i**, 104.0 ppm for **3j**, 103.8 ppm for **3k**, 105.5 ppm for **3l** and 104.2 ppm for both **3m** and **3n** are ascribed to the carbon at the cyclic ether bound to the hemiketal hydroxyl, while the peak in the region of 200 ppm, typical to the carbonyl ^13^C nucleus, was absent. A broad singlet at 8.86 ppm for **3a**, 8.85 ppm for **3b**, 8.75 ppm for **3c**, 8.95 ppm for **3d**, 8.61 ppm for **3e**, 9.05 ppm for **3f**, 9.16 ppm for **3g**, 9.43 ppm for **3h**, 8.9 ppm for **3i**, 8.1 ppm for **3j**, 8.0 ppm for **3k**, 8.68 ppm for **3l**, 8.26 ppm for **3m** and 8.27 ppm for **3n** in the respective ^1^H NMR spectra was assigned to a hydroxyl group. The chemical shifts of the protons in the triazole ring were environment-dependent (8.04, 8.36 ppm for **3a**; 8.04, 8.36 ppm for **3b**; 8.09, 8.39 ppm for **3c**; 8.04, 8.14–8.06, 8.40 ppm for **3d**; 8.02, 8.32 ppm for **3e**; 8.06, 8.48 ppm for **3f**; 8.03–7.96, 8.48 ppm for **3g**; 8.51, 8.55 ppm for **3h**; 8.06–8.11, 8.50 ppm for **3i**; 8.1, 8.21 ppm for **3j**; 8.18, 8.69 ppm for **3k**; 8.27, 8.72 ppm for **3l**; 8.18, 8.7 ppm for **3m;** 8.17, 8.7 ppm for **3n**). The structures of compounds were confirmed by recording their mass spectra. The molecular ion of **3a** appeared at *m/z* 370.2/371.1 corresponding to the molecular formula C_17_H_12_BrN_3_O_2_, which is consistent with the structure assigned to it. The characterization data of compounds **3a**–**n** are given in experimental part. 

**Figure 4:**
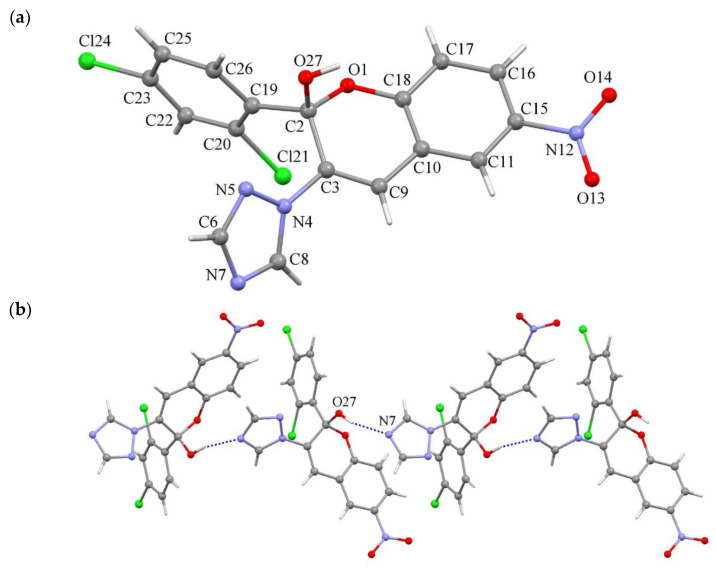
Molecular structure of 3 h determined by single-crystal X-ray diffraction method (**a**), a fragment of crystal packing in structure 3 h illustrates the formation of intermolecular hydrogen bonds O27–H… N7, which bind 3 h molecules into a chain (**b**).

The structure of the compound **3h** was characterized by a single-crystal X-ray diffraction method. The compound crystallizes in the centrosymmetric monoclinic space group *C*2/*c*; therefore, it is a racemate. The unit cell parameters were: *a* = 24.207(2), *b* = 9.7963(7), *c* = 14.9130(13) Å, *β* = 100.229(8)° and *V* = 3480.2(5) Å^3^. The structure of the molecule is shown in Figure 3. The nitro group was nearly co-planar to the chromenol fragment and the corresponding dihedral angle was 10.00°, while triazole and 1,3-dichlorobenzene fragments formed with the root mean square plane determined by the atoms of the chromenol fragment dihedral angles of 50.79 and 89.81°, respectively. The length of the double C3–C9 bond in the chromenol fragment equals 1.318(5) Å, in the 1,2,4-triazole fragment all bonds were delocalized as indicated by their length and the formally double N5–C6 and N7–C8 bonds were longer than expected for double bonds, equal 1.315(5) and 1.312(4) Å, respectively, and formally single bonds lengths were shorter than expected and equaled N4–N5 = 1.365(4), N4–C8 = 1.337(4) and N7–C6 = 1.354(5) Å, while the bond length N4–C3 = 1.420(4) Å corresponded to a single one. The molecule of **3h** formed an infinite chain due to the O27–H···N7 hydrogen bonds, along the c crystallographic axis (Figure 4b).

### 2.2. PASS Prediction and Similarity Search in CDDI Database

A PASS prediction of antifungal activity was performed for the whole set of designed molecules, from which fourteen were chosen for synthesis and biological testing. Antifungal activity for all selected compounds was predicted with the probability to be active Pa values ranging from 0.43 to 0.53 (Table 1). The mechanism of antifungal activity was predicted as well. The prediction revealed that lanosterol 14 alpha demethylase inhibition was estimated with Pa between 0.16 and 0.35. The calculated Pa values for most of the compounds were less than 0.5, indicating their relative novelty compared to the structures of the compounds from the PASS training set [56,57]. Such results lead to the conclusion that the studied compounds have some features different from those of well-known antifungal agents, which may indicate their innovative potential. To check this inference, we performed a chemical similarity search in the Cortellis Drug Discovery Intelligence (CDDI) database [58], one of the most comprehensive sources of current information about pharmacological agents. The CDDI contains data on more than 580 thousand pharmaceutical agents, 90% of which includes a chemical structure. Using the similarity search implemented in the CDDI, no close analogs of the compounds under study were identified with the Tanimoto coefficient (TC) exceeding 70%. Earlier, Yvonne Martin and co-authors [59] showed that even at the TC > 85%, there is only a 30% chance to find compounds with the same biological activity. Thus, the similarity search results in the CDDI database confirm that the designed compounds have a high novelty compared to the known antifungal agents.

### 2.3. Biological Evaluation

#### Antifungal Activity

Compounds **3a**–**3n** were evaluated by a microdilution method against a panel of eight fungi using bifonazole and ketoconazole as reference drugs. All compounds showed antifungal activity, and the results are presented in Table 2.

The order of antifungal activity can be presented as follows: **3k** > **3n** > **3g** > **3m** > **3d** > **3c** > **3h** > **3b** > **3e** > **3l** > **3a** > **3i** > **3f** > **3j**. The best antifungal activity was achieved for compound **3k** with MIC ranging 22.1–184.2 µM and MFC at 46.0–368.5 µM. The lowest antifungal activity was exhibited by compound 3j with MIC and MFC at 143.9–1151 µM and 575.6–1439 µM, respectively.

As far as reference drugs are concerned, ketoconazole showed an antifungal potential with MIC at 380–4750 µM and MFC at 950–5700 µM, whereas bifonazole showed MIC at 480–640 µM and MFC at 640–800 µM. Thus, all compounds were more effective antifungal agents than ketoconazole, and almost all (twelve from fourteen) exhibited a higher activity than bifonazole.

It is interesting to mention that the fungi showed a different sensitivity towards the compounds tested. Thus, the order of activity of tested compounds against the most sensitive fungi *T. viride* is **3k** = **3n** > **3b** = **3g** = **3m** > **3c** = **3d** = **3e** = **3h** = **3i** = **3l** = **3j** > **3a** > **3f,** whereas the sensitivity of the most resistant fungi, *A. fumigatus,* can be presented as **3d** = **3e** = **3k** > **3g** = **3h** = **3m** > **3b** = **3n** > **3c** = **3l** > **3j** > **3a** = **3i** > **3f**. Not only were there differences in the sensitivity among species, but also in each fungus were observed. At the same time, all species appeared to be sensitive to compound **3k** and not sensitive to **3f** and **3j**.

Compound **3k** showed perfect activity with MIC 22.1 µM against *A. niger*. It also exhibited good activity with MIC 33.1 µM against *A. fumigatus*, and *T. viride* and *A. versicolor* with MIC 92.1 µM. The same good activity was achieved against *T. viride* by compound 3n, against *A. versicolor* by 3d and 3e, and against *P. ochraceus* by **3b**, **3c**, **3d,** and **3l**.

The study of the structure–activity relationship revealed that the presence of the 2-(tert-butyl)-2*H*-chromen-2-ol substituent **3k** on the 4th position of the triazole ring was very beneficial for antifungal activity. The introduction on 2-(tert-butyl)-2*H*-chromen-2-ol bromine at position six, resulted in a slightly less active compound **3n**, while substitution on position two of the 2-(tert-butyl)-2*H*-chromen-2-ol by 2,4-dichlorophenyl group (in **3g**) decreased the antifungal activity more. In series of 2-H chromen-2-ol derivatives with a tert-butyl substituent in position two of the chromen ring, the most beneficial was the unsubstituted benzene (**3k**) derivative followed by derivatives with a substituted benzene ring with electron withdrawal (EWG) substituents (Br, Cl, NO_2_), while the electron-donating (EDG) substituent (Ph) (**3j**) was detrimental for antifungal activity of these derivatives. In the case of the 2,4-dichlorophenyl substituent in position two of the chromen-2-ol moiety, the favorable effect was observed for the derivative with bromine (**3g**) as a substituent in the benzene ring. The order of activity can be presented as Br > NO_2_ > Ph > Cl. There is no strong correlation of activity with the EWG/EDG character of substituents in this case. From all mentioned above, it can be concluded that antifungal activity depends not only on the triazole ring, but also on its substituents.

### 2.4. Docking Studies

All the synthesized compounds and the reference drug ketoconazole were docked to lanosterol 14α-demethylase (known as well as cytochrome 51-CYP51) of *C. albicans* and DNA topoisomerase IV (Table 3). Results revealed that the values of free binding energy for DNA topoisomerase IV were higher than CYP51. Thus, the inhibition of this enzyme may be the putative mode of action of the analyzed novel chromenol derivatives.

**Figure 5:**
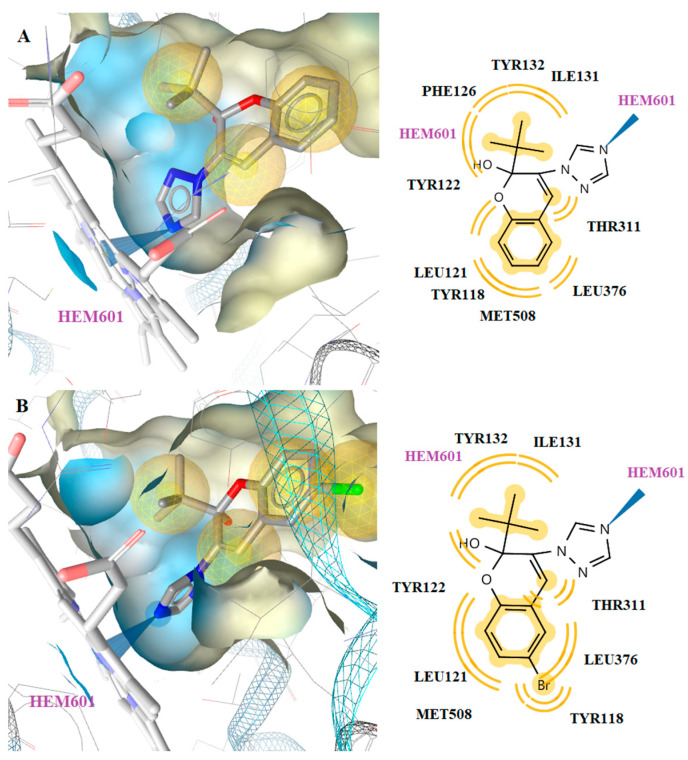
Docked conformation of compounds **3k** (**A**) and **3n** (**B**) in lanosterol 14α-demethylase of *C*. *albicans* (CYP51).

Docking results revealed that the most active compound, **3k**, bound the CYP51 enzyme of *C. albicans* in a way that allowed the interaction with the heme. In particular, the N atom of the piperazine ring of the compound interacted strongly with the Fe of the heme group. Moreover, hydrophobic interactions were detected with the residues Tyr118, Ile131, Tyr132, Phe126, Tyr122, Leu121, Thr311, Leu376 and Met508. Hydrophobic interactions were also observed between the methyl substituents and the heme group of the enzyme. The same interactions were detected in the binding of compound **3n** (Figure 5).

**Figure 6:**
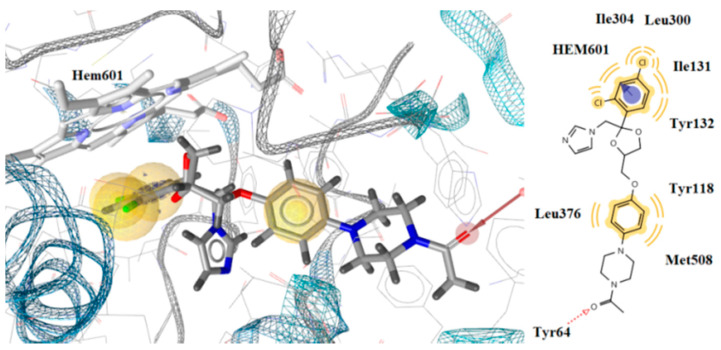
Docking of ketoconazole on CYP51of *Candida albicans*.

Interaction with the heme group was also observed with the benzene ring of ketoconazole, which formed a hydrophobic and aromatic interaction (Figure 6). However, compounds **3k** and **3n** had stronger interactions than ketoconazole and formed more stable complexes with the enzyme which is the likely reason for the better antifungal activity than ketoconazole.

### 2.5. In Silico Predictive Studies

Drug-likeness is an essential part of drug research and development that provides the base for the molecules to be powerful drug candidates. Various rules, such as, e.g., Lipinski, Ghose, Veber, Egan, and Muegge, were considered to measure the drug-likeness of the tested compounds to find out whether they can be bioactive drug candidates according to some critical criteria such as the molecular weight, LogP, number of hydrogen bond acceptors and donors. The number of violations to the rules, along with bioavailability and drug-likeness scores, are given in Table 4. The results revealed that none of the compounds violated any rule, and their bioavailability score was around 0.55. All the tested molecules were suggested to pass the blood–brain barrier (BBB) except for in compounds **3h** and **3l**. All compounds exhibited moderate to good drug-likeness scores, ranging from −0.63 to 0.29.

**Figure 7:**
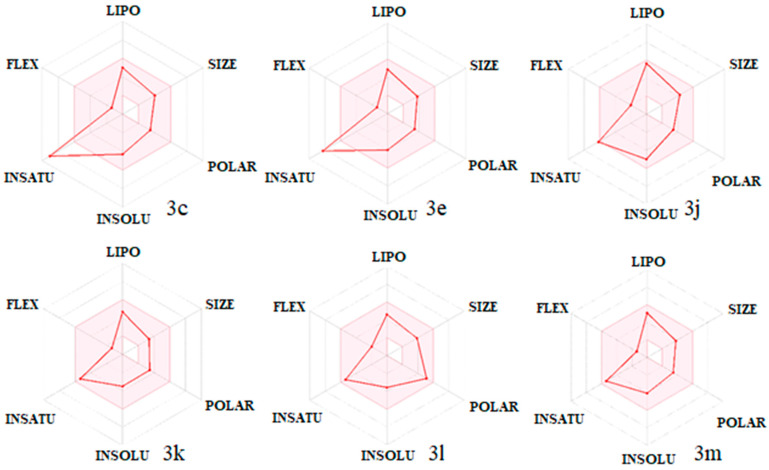
Bioavailability radar of the tested compounds. The pink area represents the optimal range for each property for oral bioavailability. Lipophilicity (LIPO): XLOGP3 between −0.7 and +5.0; molecular weight (SIZE): MW between 150 and 500 g/mol; polarity (POLAR): TPSA between 20 and 130 Å^2^; solubility (INSOLU): log S not higher than 6; saturation (INSATU): fraction of carbons in the sp3 hybridization not less than 0.25; flexibility (FLEX): no more than 9 rotatable bonds.

Moreover, the bioavailability radar of some of the compounds is displayed in Figure 7. The most active compound, **3k**, appeared to be the best in the in silico predictions, with a drug-likeness score of 0.29 without any rule violation. However, compounds **3k** and **3n** had stronger interactions than ketoconazole and formed more stable complexes with the enzyme which are the likely reasons for the better antifungal activity than ketoconazole.

### 2.6. Cytotoxicity Studies

To ensure the safety margin for the tested compounds, cytotoxicity was tested in both the cancerous breast cancer cell line MCF7/S0.5 and the non-cancerous cell renal epithelial line HK-2.

**Figure 8:**
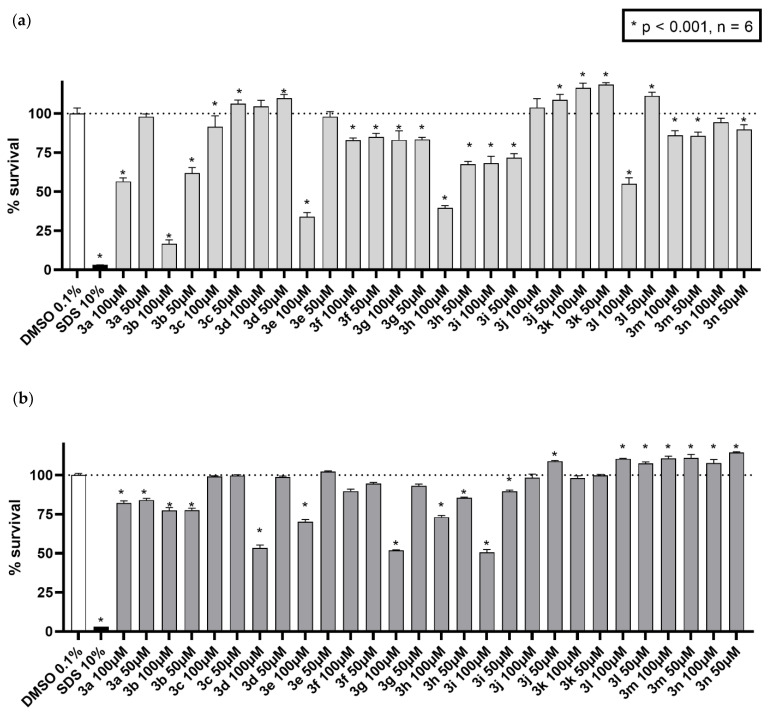
Cytotoxicity activity of the tested compounds. The fourteen derivates were tested in (**a**) breast cancer cell line MCF7/S0.5 and (**b**) renal epithelia HK-2 cell line for 48 h in two high concentrations. Results are expressed as the mean ± s.d. of three independent experiments performed in triplicates, where 100% survival was set to the positive control wells (DMSO 0.1%). Statistical assay one-way ANOVA was performed to assess the significance of the results compared to positive control. * *p* < 0.001 vs. the negative control DMSO.

Initial screening at a high concentration was performed in the MCF7/S0.5 cell line. At a concentration of 100 µM, seven compounds (**3c**, **d**, **j**, **k**, **l**, **m**, and **n**) showed little toxicity. When tested at a, still high, concentration of 50 µM, several other compounds (**3a** and **3e**) showed a higher survival compared to the 100 µM results (Figure 8a). Since cultured cancer cell lines are more sensitive to xenobiotic treatment, the same experiments were performed with the non-cancerous cell line HK-2 to further confirm the safety of the compounds.’. As shown in Figure 8b, general survival values were much higher for cells treated in the same way as in MCF7/S0.5 cells, as expected. These data indicate that nine out of fourteen synthesized compounds can be considered potential candidates for furthe drug development. When integrated with antifungal activity data, the most promising compounds seemed to be **3c**, **3d**, **3k**, **3m** and **3n**.

## 3. Materials and Methods

### 3.1. Chemistry

#### 3.1.1. General Information

The chemicals used were of reagent grade. Removal of all solvents was carried out under reduced pressure. The ^1^H and ^13^C NMR spectra were recorded in d6-DMSO 2% solutions on a “Bruker Avance III” (400.13 and 100.61 MHz) (Karlsruhe, Germany). Chemical shifts δ are given in ppm referring to the signal center using the solvent peaks for reference: d6-DMSO 2.50 ppm. IR spectra were recorded on a Spectrum 100 FT-IR spectrophotometer (PerkinElmer) using the universal ATR sampling accessory Agilent 5975C VL MSD (Waltham, MA, USA) with triple axis detector, performed using the method by Druta: temperature 60–320 °C, run time: 32.333 min, flow 1.1062 mL/min, pressure 9.418 psi and method steroid 250, temperature 180–250 °C, run time 29 min, flow 1.1062 mL/ min, pressure 16.528 psi. All products were analyzed by CHN elemental analysis (Elementar Vario EL analyzer) (Santa Clara, CA, USA). Melting points (uncorrected) were determined on a Boetius apparatus (Dresden, Germany). Thin-layer chromatography was carried out on Merck aluminum TLC plates, silica gel 60 coated with fluorescent indicator F254.

#### 3.1.2. Synthesis

For the preparation of tested compounds, the following method was used: One-necked flask equipped with a Dean–Stark receiver and reflux condenser were charged with triazolyl ketone (10 mmol), aldehyde (11 mmol), catalyst (1 mol%, mixture of acetic acid and piperidine 1:1) and benzene (100 mL). The resulting mixture was heated in an oil bath to maintain a gentle solvent reflux for 18 h. The reaction was monitored by TLC. After cooling to room temperature, the reaction mixture was washed with water (2 × 50 mL) in a separation funnel, dried with magnesium sulphate and distilled in vacuo.

**6-Bromo-2-phenyl-3-(1*H*-1,2,4-triazole-1-yl)-2*H*-chromen-2-ol (3a)**. Yield 65%, white crystals (ethanol), mp 97–99 °C. IR (ν/cm^−1^): 3676, 3061, 2989, 2902, 1652, 1576, 1479, 1436, 1281, 1249, 1210, 1140, 1075, 967, 880, 760, 699, 673; ^1^H NMR (400 MHz, DMSO-d_6_): 8.85 (1H, s, broad), 8.36 (1H, s), 8.04 (1H, s), 7.75 (1H, d, J = 2.4 Hz), 7.52 (2H, dd, J = 7.7, 1.8 Hz), 7.43 (2H, d, J = 1.9 Hz), 7.32 (3H, dd, J = 5.0, 1.4 Hz), 6.93 (1H, d, J = 8.7 Hz); ^13^C NMR (100 MHz, DMSO): 152.1, 149.8, 144.1, 140.9, 132.9, 131.9, 130.3, 129.4, 128.7, 126.4, 121.7, 118.4, 16.8, 113.3, 98.1. Anal. Calcd for C_17_H_12_BrN_3_O_2_ C 55.15; H 3.27; N 11.35.%. Found C 54.8; H 2.9; N 10.8%. MS: calcd for m/z 370.2, found 371.1.

**6-Chloro-2-phenyl-3-(1*H*-1,2,4-triazole-1-yl)-2*H*-chromen-2-ol (3b)**. Yield 43%, white crystals (ethanol), mp 92–94 °C. IR (ν/cm^−1^): 3676, 3061, 2989, 2902, 1652, 1576, 1479, 1436, 1281, 1249, 1210, 1140, 1075, 967, 880, 760, 699, 673; ^1^H NMR (400 MHz, DMSO-d_6_): 8.86 (1H, s, broad), 8.36 (1H, s), 8.04 (1H, s), 7.75 (1H, d, J = 2.4 Hz), 7.53–7.51 (2H, m), 7.44 (2H, d, J = 7.6 Hz), 7.33 (3H, td, J = 5.9, 3.0 Hz), 6.93 (1H, d, J = 8.7 Hz); ^13^C NMR (100 MHz, DMSO): 152.08, 149.76, 144.06, 140.86, 132.96, 131.83, 130.28, 129.37, 128.66, 126.39, 121.19, 118.37, 116.72, 113.31, 98.01. Anal. Calcd for C_17_H_12_ClN_3_O_2_ C 62.68; H 3.71; N 12.90%. Found C 62.1; H 3.53; N 12.45%. MS: calcd for m/z 324.75, found 327.0.

**2-(2-Chlorophenyl)-3-(1*H*-1,2,4-triazole-1-yl)-2*H*-chromen-2-ol (3c)**. Yield 71%, white crystals (ethanol), mp 193–195 °C. IR (ν/cm^−1^): 3628, 3115, 2988, 2301, 1654, 1573, 1486, 1444, 1279, 1238, 1200, 1131, 1001, 877, 756, 691, 661; ^1^H NMR (400 MHz, DMSO-d_6_): 8.75 (1H, s, broad), 8.39 (1H, s), 8.09 (1H, dd, J_1_ = 7.9 Hz, J_2_ = 1.6 Hz), 7.95 (1H, s), 7.42–7.29 (6H, m), 7.08 (1H, td, J_1_ = 7.5 Hz, J_2_ = 0.9 Hz), 6.99 (1H, d, J = 8.1 Hz); ^13^C NMR (100 MHz, DMSO-d6): 151.9, 150.9, 144.0, 137.7, 132.9, 131.2, 131.1, 130.7, 129.74, 129.7, 128.4, 127.0, 122.1, 118.9, 118.8, 116.3, 95.9. Anal. Calcd for C_17_H_12_ClN_3_O_2_ C 62.68; H 3.71; N 12.90%. Found C 62.0; H 3.2; N 12.0.%. MS: calcd for m/z 325.75, found 327.0.

**6-Chloro-2-(2-chlorophenyl)-3-(1*H*-1,2,4-triazole-1-yl)-2*H*-chromen-2-ol (3d)**. Yield 53%, white crystals (ethanol), mp 167–171 °C. IR (ν/cm^−1^): 3630, 3126, 3035, 2309, 1651, 1571, 1482, 1441, 1285, 1244, 1211, 1134, 1104, 969, 894, 762, 695, 670; ^1^H NMR (400 MHz, DMSO-d_6_): 8.95 (s, 1H, broad), 8.40 (1H, s), 8.14–8.06 (1H, m), 7.99 (1H, s), 7.63 (1H, d, J = 2.6 Hz), 7.60–6.59 (5H, m), 7.03 (1H, d, J = 8.7 Hz); ^13^C NMR (100 MHz, DMSO-d_6_): 152.06, 149.59, 144.08, 137.41, 132.89, 131.37, 131.12, 130.80, 130.05, 129.72, 127.47, 127.12, 125.76, 120.69, 118.05, 117.58, 96.13. Anal. Calcd for C_17_H_11_Cl_2_N_3_O_2_ C 56.69; H 3.08; N 11.67%. Found C 56.1; H 2.7; N 11.1%. MS: calcd for m/z 360.19, found 361.1

**2-(4-methylphenyl)-3-(1*H*-1,2,4-triazole1-yl)-2*H*-chromen-2-ol (3e)**. Yield 30%, white crystals (ethanol), mp 87–90 °C. IR (ν/cm^−1^): 3658, 3160, 3086, 2989, 1655, 1578, 1487, 1456, 1284, 1241, 1219, 1146, 1111, 1039, 967, 896, 753, 693, 669; ^1^H NMR (400 MHz, DMSO-d_6_): 8.61 (1H, s, broad), 8.32 (1H, s), 8.02 (1H, s), 7.47 (1H, dd, J = 7.5, 1.5 Hz), 7.39 (2H, d, J = 8.0 Hz), 7.38 (1H, s), 7.29 (1H, td, J = 7.8, 1.5 Hz), 7.13 (2H, d, J = 8.0 Hz), 7.04 (1H, td, J = 7.4, 1.0 Hz), 6.92 (1H, d, J = 8.3 Hz), 2.26 (3H, s); ^13^C NMR (100 MHz, DMSO-d_6_): 151.9, 150.6, 143.9, 138.6, 138.5, 131.1, 130.7, 129.1, 128.3, 126.3, 122.0, 118.9, 117.9, 116.0, 97.9, 21.1. Anal. Calcd for C_18_H_15_N_3_O_2_ C 70.81; H 4.95; N 13.76%. Found C 70.1; H 4.5; N 13.4%. MS: calcd for m/z 305.33, found 307.1.

**6-Chloro-2-(2,4-dichlorophenyl)-3-(1*H*-1,2,4-triazole-1-yl)-2*H*-chromen-2-ol (3f)**. Yield 75%, white crystals (benzene), mp 193–195 °C. IR (ν/cm^−1^): 3113, 3082, 2989, 1660, 1584, 1480, 1341, 1259, 1250, 1129, 1088, 999, 898, 825, 784, 677, 663; ^1^H NMR (400 MHz, DMSO-d6): 9.05 (1H, s), 8.48 (1H, s), 8.06 (1H, d, J = 8.9 Hz), 7.99 (1H, s), 7.61 (1H, d, J = 2.7 Hz), 7.49 (2H, d, J = 6.5 Hz), 7.36 (2H, q, J = 4.7, 3.5 Hz), 7.03 (1H, d, J = 8.7 Hz); ^13^C NMR (100 MHz, DMSO-d6): 152.23, 149.45, 144.41, 136.60, 135.09, 133.82, 131.25, 130.43, 130.30, 130.23, 127.53, 127.28, 125.88, 120.59, 118.16, 118.09, 95.81. Anal. Calcd for C_17_H_10_Cl_3_N_3_O_2_; C 51.74; H 2.55; N 10.65%. Found C 50.9; H 1.9; N 10.1.%. 

**6-Bromo-2-(2,4-dichlorophenyl)-3-(1*H*-1,2,4-triazole-1-yl)-2*H*-chromen-2-ol (3g)**. Yield 89%, white crystals (ethanol), mp 195–197 °C. IR (ν/cm^−1^): 3628, 3040, 2767, 2309, 1646, 1560, 1476, 1376, 1280, 1240, 1126, 1099, 994, 892, 825, 781, 731, 699; ^1^H NMR (400 MHz, DMSO-d6): 9.16 (1H, s, broad), 8.48 (1H, s), 8.03–7.96 (1H, m), 7.93 (s, 1H), 7.66 (1H, d, J = 2.6 Hz), 7.44 (3H, dt, J = 9.2, 2.6 Hz), 7.28 (1H, s), 6.93 (1H, d, J = 8.7 Hz); ^13^C NMR (100 MHz, DMSO-d6): 151.96, 149.82, 144.42, 136.33, 135.26, 133.70, 133.30, 131.12, 130.47, 130.37, 130.04, 128.79, 127.27, 120.89, 118.59, 113.59, 95.74. Anal. Calcd for C_17_H_10_BrCl_2_N_3_O_2_ C 46.50; H 2.30; N 9.57%. Found C 46.0; H 1.9; N 9.1.%.

**2-(2,4-Dichlorophenyl)-6-nitro-3-(1*H*-1,2,4-triazole-1-yl)-2*H*-chromen-2-ol (3h)**. Yield 25%, white crystals (ethanol), mp 193–195 °C. IR (ν/cm^−1^): 3660, 3114, 2989, 1660, 1584, 1481, 1340, 1259, 1251, 1129, 1088, 1000, 898, 825, 784, 677, 663; ^1^H NMR (DMSO-d6, 400 MHz): 9.43 (1H, s), 8.55 (1H, s), 8.51 (1H, s), 8.21 (1H, d, J = 7 Hz), 8.08 (1H, d, J = 9 Hz), 8.01 (1H, s), 7.59 (1H, s), 7.51 (1H, s), 7.51 (1H, d, J = 8 Hz), 7.22 (1H, d, J = 10 Hz); ^13^C NMR (DMSO-d6, 100 MHz): 155.8, 152.4, 146.6, 142.3, 136.0, 135.4, 133.8, 131.3, 130.7, 130.5, 127.4, 126.3, 124.2, 119.3, 118.0, 117.2, 96.8. Anal. Calcd for C_17_H_10_C_l2_N_4_O_4_; C, 50.39; H, 2.49; N, 13.83%. Found C, 50.1; H, 2.0; N, 13.5%. 

**2-(2,4-Dichlorophenyl)-6-phenyl-3-(1*H*-1,2,4-triazole-1-yl)-2*H*-chromen-2-ol (3i)**. Yield 40%, white crystals (ethanol), mp 191–193 °C. IR (ν/cm^−1^): 3165, 3061, 2990, 1652, 1509, 1477, 1434, 1283, 1249, 1232, 1147, 1056, 987, 874, 760, 699, 672; ^1^H NMR (400 MHz, DMSO-d_6_): 8.9 (1H, s, broad), 8.50 (1H, s), 8.11- 8.06 (1H, m), 7.9 (1H, s), 7.83 (1H, d, J = 2.2 Hz), 7.7 (1H, d, J = 7.3 Hz), 7.6 (2H, dd, J = 8.5, 2.3 Hz), 7.53–7.46 (3H, m), 7.4 (1H, s), 7.40–7.33 (m, 2H), 7.11 (1H, d, J = 8.5 Hz); ^13^C NMR (101 MHz, DMSO-d_6_): 152.1, 150.4, 144.3, 139.9, 136.9, 134.9, 134.5, 133.9, 131.3, 130.4, 129.7, 129.4, 129.1, 128.8, 127.6, 127.2, 126.8, 126.6, 119.4, 116.8, 95.9. Anal. Calcd for C_23_H_15_Cl_2_N_3_O_2_ C 63.32; H 3.47; N 9.63%. Found C 62.8; H 2.9; N 9.1%.

**2-tert-Butyl-6-phenyl-3-(1*H*-1,2,4-triazole-1-yl)-2*H*-chromen-2-ol (3j)**. Yield 67%, white crystals (benzene), mp 220 °C. IR (ν/cm^−1^): 3309, 2974, 1610, 1508, 1481, 1456, 1426, 1293, 1260, 1202, 1138, 1113, 1063,987, 891, 825, 764,692, 677; ^1^H NMR (DMSO-d6, 400 MHz): 8.72 (1H, s), 8.21 (1H, s), 8.1 (1H, s), 7.67 (2H, d, J = 2.3 Hz), 7.63 (2H, dd, J1 = 8.3 Hz, J_2_ = 1.3 Hz), 7.58 (1H, dd, J_1_ = 8.5 Hz, J_2_ = 2.3 Hz), 7.45 (2H, t, J = 8.0 Hz), 7.33 (1H, d, J = 7.4 Hz), 7.23 (1H,s), 7.02 (1H, d, J = 8.5 Hz), 0.84 (9H, s); ^13^C NMR (DMSO-d6, 100 MHz): 152.7, 151.9, 145.2, 139.9, 133.4, 130.3, 129.4, 129.2, 128.8, 127.4, 126.6, 126.2, 123.2, 119.3, 115.3, 104.0, 42.9, 24.7. Anal. Calcd for C_21_H_21_N_3_O_2_; C, 72.60; H, 6.09; N, 12.10.%. Found 71.8.3; H 5.8; N 11.6.%. MS: calcd for m/z 347.41, found 347.1.

**2-tert-Butyl-3-(1*H*-1,2,4-triazole-1-yl)-2*H*-chromen-2-ol (3k)**. Yield 70%, white crystals (ether), mp 150–151 °C. IR (ν/cm^−1^): 3068, 2977, 1631, 1508, 1485, 1459, 1411, 1284, 1233, 1156, 1125, 1062, 997, 875, 759, 749, 673, 653; ^1^H NMR (DMSO-d6, 400 MHz): 8.69 (1H, s),8.18 (1H, s), 8.0 (1H, s, broad), 7.28 (1H, dd, J_1_ = 7,4Hz, J_2_ = 0.8Hz), 7.25 (1H, td, J = 7.8 Hz, J = 1.4 Hz), 7.10 (1H, s), 6.9 (1H, td, J = 7.4 Hz, J = 0.8 Hz), 6.89 (1H, d, J = 8.2 Hz), 0.7 (9H, s); ^13^C NMR (DMSO-d6, 100 MHz): 152.9, 151.8, 145.1, 131.1, 129.8, 128.3, 121.4, 123.3, 118.7, 114.8, 103.8, 42.9, 24.6. Anal. Calcd for C_15_H_17_N_3_O_2_; C 66.41; H 6.32; N 15.49%. Found 67.3; H 6.00; N 14.82.%. MS: calcd for m/z [C_15_H_17_N_3_O_2_]^+^ 271.31, found 214.0 [M-C(CH_3_)_3_].

**2-tert-Butyl-6-nitro-3-(1*H*-1,2,4-triazole-1-yl)-2*H*-chromen-2-ol (3l)**. Yield 40%, yellow crystals (ethanol), mp 173–175°C. IR (ν/cm^−1^): 3164, 2982, 1665, 1503, 1483, 1445, 1338, 1273, 1131, 1091, 1064, 971, 883, 751, 728, 663; ^1^H NMR (DMSO-d6, 400 MHz): 8.72 (1H, s), 8.68 (1H, s, broad), 8.27 (1H, d, J = 2.2 Hz), 8.20 (1H, s), 8.13 (1H, d, J_1_ = 9.0 Hz, J_2_ = 2.8 Hz), 7.30 (1H, s), 7.1 (1H, d, J = 9.0 Hz), 0.74 (9H, s); ^13^C NMR (DMSO-d6, 100 MHz): 158.3, 152.5, 145.2, 141.4, 131.5, 126.8, 123.9, 121.5, 119.2, 115.9, 105.5, 43.0, 24.5. Anal. Calcd for C_15_H_16_N_4_O_2_; C 56.96; H 5.10; N 15.18.%. Found C 55.3; H 4.8; N 14.82.%. MS: calcd for m/z [C_15_H_16_N_4_O_2_]^+^ 316.31, found 259.0 [M-C(CH_3_)_3_]. 

**2-tert-Butyl-6-chloro-3-(1*H*-1,2,4-triazole-1-yl)-2*H*-chromen-2-ol (3m)**. Yield 56%, white crystals (ethanol), mp 160–161°C. IR (ν/cm^−1^): 3297, 3060, 2964, 1645, 1505, 1480, 1468, 1428, 1279, 1233, 1144, 1118, 1063, 971, 873, 799, 731, 677, 661; ^1^H NMR (DMSO-d6, 400 MHz): 8.7 (1H, s), 8.26 (1H, s, broad), 8.18 (1H, s), 7.38 (1H, d, J = 1,6 Hz); 7.26 (1H, dd, 1H, J_1_ = 8.06 Hz, J_2_ = 2.6 Hz), 7.12 (1H, s), 6.93 (1H, d, J = 8.6 Hz), 0.73 (9H, s); ^13^C NMR (DMSO-d6, 100 MHz): 151.8, 151.7, 145.1, 130.8, 130.5, 127.2, 124.9, 122.0, 120.4, 116.7, 104.2, 43.0, 24.5. Anal. Calcd for C_15_H_16_ClN_3_O_2_; C 58.92; H 5.27; N 13.74%. Found C 58.1; H 4.8; N 13.1.%. MS: calcd for m/z 305.76, found 305.0.

**6-Bromo-2-tert-butyl-3-(1*H*-1,2,4-triazole-1-yl)-2*H*-chromen-2-ol (3n)**. Yield 70%, white crystals (ethanol), mp 182–183°C. IR (ν/cm^−1^): 3357, 3063, 2978, 1659, 1502, 1480, 1430, 1284, 1235, 1130, 1055, 1000, 970, 831, 731, 661; ^1^H NMR (DMSO-d6, 400 MHz): 8.7 (1H, s), 8.27 (1H, s, broad), 8.17 (1H, s), 7.47 (1H, d, J = 2.0 Hz), 7.37 (1H, dd, 1H, J_1_ = 8.6 Hz, J_2_ = 2.4 Hz), 7.10 (1H, s), 6.86 (1H, d, J = 8.6 Hz), 0.73 (9H, s); ^13^C NMR (DMSO-d6, 100 MHz): 152.2, 151.8, 145.1, 133.4, 130.7, 130.0, 122.0, 121.0, 117.2, 112.4, 104.2, 43.0, 24.5. Anal. Calcd for C_15_H_16_BrN_3_O_2_; C 51.44; H 4.60; N 12.00.Found C 50.9; H 4.1; N 11.8.%. MS: calcd for *m*/*z* 350.21, found 351.1.

### 3.2. Crystallographic Study

Diffraction measurements for single-crystal X-ray analysis of compound **3h** were carried out on an Xcalibur E diffractometer (Cambridge, Great Britain) equipped with a CCD area detector and a graphite monochromator utilizing MoKα radiation at room temperature. All calculations to solve and refine the structure were carried out with the programs SHELXS-97 and SHELXL-2014 [60,61]. Non-hydrogen atoms were refined anisotropically. Positions of H atoms were calculated geometrically and refined isotropically using a rigid-body model. The 1,3-dichlorobenzene moiety of the **3h** molecule was found to be disordered in the structure over two coplanar positions with probabilities of their occupancy 0.591(3):0.409(3). The structure was refined using 3079 (*R*(int) = 0.0512) independent reflections to *R*1 = 0.0630 and *wR*2 = 0.0727 for 1478 reflections with *I* > 2*σ*(*I*) and *GOF* = 1.003. The maximum and minimum residual electron densities in the difference synthesis were 0.196 and −0.277eÅ^–3^. Crystallographic data were deposited with The Cambridge Crystallographic Data Centre, CCDC 2033740, and may be obtained free of charge from The Cambridge Crystallographic Data Centre. 

### 3.3. PASS Predictions and Similarity Assessment

The online version of the computer program PASS (Prediction of Activity Spectra for Substances) [56,57] predicts about 4000 biological activities with an average accuracy of about 95%. Thousands of researchers widely use it from over 100 countries to identify the most promising directions of biological testing of the designed and synthesized compounds. Structural formulae of compounds presented as MOL or SDF files are used as input data. Predictions are presented as a list of probable activities with two probabilities that reflect the chance of compounds belonging to active (Pa) and inactive (Pi) classes. A high Pa value could be obtained for the compounds with close analogs in the PASS training set. If, for a particular activity, the Pa value is less than 0.5, the compound does not have close analogs with this activity in the PASS training set. 

The Cortellis Drug Discovery Intelligence database [58] is one of the most comprehensive informational sources continually curated and updated by the skilled team manually annotated data about pharmaceutical research and development performed worldwide. Currently, the CDDI includes more than 500 thousand chemical structures of pharmaceutical agents under different biological testing stages. Chemical similarity search in the CDDI database allows identifying at different cutoff values (from 60 to 100%) the close analogs of the structure used as a query if such analogs are found.

### 3.4. Biological Evaluation: Antifungal Activity

The microdilution method was used to evaluate anti-fungal properties of the new chromenol–triazole hybrids, following methods described in our earlier study [62]. Fungi, namely, *Aspergillus fumigatus* (human isolate), *Aspergillus ochraceus* (ATCC 12066), *Aspergillus niger* (ATCC 6275), *Aspergillus versicolor* (ATCC 11730), *Trichoderma viride* (IAM 5061), *Penicillium funiculosum* (ATCC 36839), *Penicillium ochrochloron* (ATCC 9112) and *Penicillium verrucosum* var. *cyclopium* (food isolate), were used to investigate the anti-fungal properties of the new chromenol–triazole hybrids. Anti-microbial results were reported as minimum inhibitory (MIC) and minimum fungicidal (MFC) concentrations. Bifonazole and ketoconazole were used as positive controls for anti-fungal evaluation. All experiments were performed in duplicate and repeated three times.

### 3.5. Molecular Docking

Τhe program AutoDock 4.2^®^ software [63] was used for the docking stimulation. The free energy of binding (ΔG) of *E. coli* DNA GyrB, thymidylate kinase, *E. coli* MurA, *E. coli* primase, *E. coli* MurB, DNA topoIV and CYP51 of *C. albicans* in a complex with the inhibitors was generated using this molecular docking program. The X-ray crystal structures data of all the enzymes used were obtained from the Protein Data Bank (PDB 1S16 and 5V5Z, respectively).

To prepare proteins, all water molecules were eliminated and polar hydrogens were added, while for preparation of the inhibitors, charges were added and the rotatable bonds determined. Grid maps were calculated utilizing the AutoGrid algorithm. Autogrid Box was computed by the X-, Y- and Z-coordinates for each enzyme. Three-dimensional structures of all compounds were constructed using ChemBio3D Ultra 12.0 software (Chemical Structure Drawing Standard; PerkinElmer Informatics, Waltham, MA, USA). For the present system, the Lamarckian genetic algorithm was applied for minimization, and the following settings were used: initial population 300, 2,500,000 maximum energy ratings and 27,000 as maximum generation. The pitch was 1.0 Å, while the quaternion and pivot angle were set to 5.0 degrees. For each compound, 200 configurations were produced. The results from the AutoDock calculations were grouped using an RMSD deviation value of 1.5 Å, while the lowest-energy configuration of the largest population group was chosen as the most likely tethering configuration. The discovery studio 2017 R2 silent and LigandScout were used to display the results and process the configurations with the highest tie rating [64]. Finally, the docking process methodology was first validated by redocking all the co-crystalized original ligands in the active sites of all enzymes with deviation (RMSD) values from 0.86 to 1.63 Å.

### 3.6. In Silico Predictive Studies

Drug-likeness is one of the qualitative ideas employed for predicting drug-like property. It is designated as an intricate balance of diverse molecular and structural features which plays a pivotal task in establishing whether the specific drug candidate is alike the known drugs or not. The targeted molecules were appraised for predicting the drug-likeness based on 5 separate filters namely Egan [65], Ghose [66], Muegge [67], Veber [68] and Lipinski [69] rules, accompanying bioavailability and drug-likeness scores using the Molsoft software and SwissADME program (http://swissadme.ch, accessed on 7 July 2021) using the ChemAxon’s Marvin JS structure drawing tool.

### 3.7. Cytotoxicity Experiments

CellTiter 96^®^ Aqueous Non-Radioactive Cell Proliferation Assay (Promega Corporation, Madison, WI, USA) was performed to evaluate the in vitro effects of evaluated compounds in two cell lines. Cancerous MCF7/S0.5 cells, developed to grow in a low-sera environment, and non-cancerous HK-2 renal cells were used for these purposes. MCF7/S0.5 cells were cultivated as specified in our previous paper [70] and HK-2 cells were cultivated in high-glucose Dulbecco’s Modified Eagle Medium (DMEM) complemented with 10% FBS and 2 mM L-glutamine. The assay is based in the reduction in a tetrazolium salt which happens only in the mitochondria of viable cells. Experiments were performed according to manufacturer indications as specified previously [66]. Briefly, cells were treated with the test compounds at concentrations of 100 and 50 μM, negative control (SDS 10%) or vehicle (DMSO 0.1%) for 48 h in 96-well plates. After incubation period, MTS reagent was added to each well and incubated for further 3 h. Absorbance was measured at 490 nm using a plate reader (Hidex Sense Beta Plus plate reader, Hidex Turku, Finland). Results are expressed as the relative cell viability, considering vehicle to have 100% viability.

## 4. Conclusions

We performed, for the first time, the synthesis of 1*H*-1,2,4-triazole-functionalized chromenols via tandem reactions of 1-(alkyl/aryl)-2-(1*H*-1,2,4-triazole-1-yl) with salicylic aldehydes and the evaluation of their antifungal activity. The antifungal activity evaluation was performed by a microdilution assay using, as reference, drugs bifonazole and ketoconazole. The best activity was achieved for compound **3k**. The most sensitive fungal compounds tested were *T. viride*, whereas *A. fumigatus* was the most resistant one. It should be mentioned that almost all compounds, except for **3e**, **3j** and **3l,** against *P.v.c* appeared to be more potent than ketoconazole against all fungi tested, while many compounds were even more active than bifonazole against all fungi tested. Compound **3k** was found to be 32-fold more active than ketoconazole and 16 times more than bifonazole.

PASS predictions demonstrated that the compounds under study have a low structural similarity with the antivirals included in the training set. This conclusion was confirmed by the similarity search in the CDDI database: at the 70% similarity cutoff, no close analog was identified among the 580,000 pharmacological substances. Thus, the synthesized chromenol derivatives have a very high innovative potential in the pharmaceutical field. 

According to molecular docking studies, it seems that the inhibition of 14α-demethylase of *C. albicans* (CYP51) was involved in the mechanism of antifungal activity of compounds tested. Biological experiments have shown that the compound 3k is not toxic to cells. Finally, we can state that the derivative **3k** can be the lead compound for further discovery of more potent antifungal agents.

## Figures and Tables

**Table 1:** Predicted biological activity spectra for the studied molecules.

Compounds ID	A1, Pa	A2, Pa	Compounds ID	A1, Pa	A2, Pa
**3a**	0.43	0.16	**3i**	0.50	0.29
**3b**	0.43	0.25	**3j**	0.45	0.19
**3c**	0.49	0.27	**3k**	0.47	0.20
**3d**	0.l5	0.29	**3i**	0.49	0.23
**3e**	0.l4	0.17	**3m**	0.50	0.28
**3f**	0.51	0.33	**3n**	0.49	0.18

A1—antifungal; A2—lanosterol 14 alpha demethylase inhibitor; Pa—probability to be active.

**Table 2:** Antifungal activity of the tested compounds (μΜ).

Compounds		*A. fum.*	*A.v.*	*A.o.*	*A.n.*	*T.v.*	*P.f.*	*P.o.*	*P.v.c.*
**3a**	**MIC**	270 ± 1.0	540 ± 2.0	270 ± 1.0	270 ± 1.0	190 ± 5.0	270 ± 2.0	270 ± 1.0	270 ± 2.0
	**MFC**	540 ± 1.0	810 ± 3.0	540 ± 1.0	540 ± 2.0	270 ± 1.0	540 ± 2.0	540 ± 1.0	540 ± 3.0
**3b**	**MIC**	600 ± 2.0	210 ± 1.0	150 ± 3.0	150 ± 5.0	113 ± 1.0	150 ± 2.0	76 ± 1.0	300 ± 1.0
	**MFC**	1800 ± 40.0	300 ± 1.0	300 ± 3.0	300 ± 1.0	150 ± 1.0	600 ± 2.0	150 ± 2.0	600 ± 3.0
**3c**	**MIC**	612 ± 3.0	306 ± 1.0	150 ± 1.0	150 ± 2.0	150 ± 1.0	214 ± 2.0	76 ± 1.0	306 ± 1.0
	**MFC**	1224 ± 4.0	612 ± 2.0	306 ± 1.0	306 ± 2.0	306 ± 1.0	306 ± 1.0	150 ± 3.0	612 ± 2.0
**3d**	**MIC**	560 ± 1.0	70 ± 1.0	70 ± 2.0	140 ± 1.0	102 ± 3.0	140 ± 1.0	35 ± 1.0	194 ± 1.0
	**MFC**	1120 ± 30.0	140 ± 1.0	140 ± 1.0	280 ± 1.0	140 ± 1.0	280 ± 1.0	70 ± 2.0	280 ± 1.0
**3e**	**MIC**	654 ± 2.0	81.75 ± 1.0	81.75 ± 2.0	163 ± 1.0	163 ± 1.0	229 ± 3.0	81.75 ± 1.0	654 ± 3.0
	**MFC**	1308 ± 30.0	163 ± 1.0	163 ± 1.0	327 ± 2.0	327 ± 1.0	327 ± 3.0	163 ± 1.0	1630 ± 30.0
**3f**	**MIC**	379.5 ± 1.0	759 ± 2.0	379.5 ± 1.0	379.5 ± 1.0	189.75 ± 2.0	759 ± 2.0	253 ± 1.0	379.5 ± 1.0
	**MFC**	506 ± 2.0	1012 ± 30.0	506 ± 2.0	506 ± 1.0	253 ± 1.0	1012 ± 20.0	506 ± 1.0	506 ± 3.0
**3g**	**MIC**	113.8 ± 2.0	113.8 ± 1.0	56.9 ± 1.0	113.8 ± 1.0	79.7 ± 2.0	113.8 ± 1.0	113.8 ± 2.0	159.4 ± 3.0
	**MFC**	227.7 ± 1.0	227.7 ± 2.0	113.8 ± 1.0	227.7 ± 1.0	113.8 ± 1.0	227.7 ± 1.0	227.7 ± 2.0	227.7 ± 1.0
**3h**	**MIC**	246.7 ± 1.0	123.3 ± 1.0	123.3 ± 2.0	123.3 ± 1.0	123.3 ± 1.0	246.7 ± 1.0	246.7 ± 2.0	246.7 ± 1.0
	**MFC**	493.5 ± 2.0	246.7 ± 1.0	246.7 ± 1.0	246.7 ± 2.0	246.7 ± 1.0	493.5 ± 2.0	493.5 ± 1.0	740.1 ± 2.0
**3i**	**MIC**	458.4 ± 1.0	458.4 ± 1.0	114.6 ± 1.0	229.2 ± 1.0	114.6 ± 1.0	229.2 ± 2.0	229.2 ± 1.0	229.2 ± 1.0
	**MFC**	916.8 ± 3.0	687.6 ± 2.0	229.2 ± 1.0	458.4 ± 1.0	229.2 ± 1.0	458.4 ± 1.0	458.4 ± 2.0	458.4 ± 1.0
**3j**	**MIC**	431.7 ± 1.0	431.7 ± 1.0	201.4 ± 1.0	143.9 ± 1.0	143.9 ± 2.0	431.7 ± 1.0	1151 ± 30.0	1151 ± 20.0
	**MFC**	575.6 ± 1.0	575.6 ± 2.0	287.8 ± 1.0	287.8 ± 1.0	287.8 ± 1.0	575.6 ± 2.0	1439 ± 30.0	1439 ± 20.0
**3k**	**MIC**	33.1 ± 1.0	92.1 ± 1.0	184.2 ± 2.0	22.1 ± 1.0	92.1 ± 1.0	184.2 ± 1.0	184.2 ± 1.0	184.2 ± 1.0
	**MFC**	46.0 ± 1.0	184.2 ± 2.0	368.5 ± 1.0	46.0 ± 1.0	184.2 ± 1.0	368.5 ± 1.0	368.5 ± 1.0	368.5 ± 2.0
**3l**	**MIC**	316.1 ± 1.0	316.1 ± 1.0	158 ± 1.0	316.1 ± 1.0	158 ± 1.0	632.2 ± 2.0	79 ± 1.0	632.2 ± 1.0
	**MFC**	632.2 ± 2.0	632.2 ± 1.0	316.1 ± 1.0	632.2 ± 3.0	316.1 ± 1.0	1264.5 ± 30	158 ± 1.0	1264.5 ± 40
**3m**	**MIC**	229 ± 2.00	163.5 ± 1.0	163.5 ± 1.0	81.7 ± 1.0	115 ± 2.0	163.5 ± 1.0	163.5 ± 1.0	163.5 ± 1.0
	**MFC**	327 ± 1.0	327 ± 1.0	327 ± 2.0	327 ± 1.0	163.5 ± 1.0	327 ± 2.0	327 ± 1.0	327 ± 2.0
**3n**	**MIC**	142.7 ± 1.0	199.8 ± 2.00	71.3 ± 2.0	71.3 ± 1.0	71.3 ± 2.0	142.7 ± 1.0	99.9 ± 2.0	142.7 ± 1.0
	**MFC**	285.5 ± 1.0	285.5 ± 2.0	142.7 ± 1.0	142.7 ± 1.0	142.7 ± 2.0	285.5 ± 1.0	142.7 ± 1.0	285.5 ± 2.0
**Ket/zole**	**MIC**	380 ± 1.20.0	2850 ± 68.0	380 ± 1.20.0	380 ± 8.20.0	4750 ± 58.0	380 ± 1.6	3800 ± 58.0	380 ± 1.2
	**MFC**	950 ± 2.3	3800 ± 84.0	950 ± 3.3	950 ± 6.3	5700 ± 86.0	950 ± 2.6	3800 ± 48.0	950 ± 2.3
**Bif/zole**	**MIC**	480 ± 2.2	480 ± 1.2	480 ± 2.8	480 ± 1.2	640 ± 2.8	640 ± 1.2	480 ± 2.0	480 ± 2.2
	**MFC**	640 ± 3.4	640 ± 0.8	800 ± 1.8	640 ± 2.3	800 ± 3.8	800 ± 2.1	640 ± 1.6	640 ± 3.4

Experiments were performed in duplicate and repeated three times. Values are expressed as means ± SD. *A.fum.*—*Aspergillus fumigatus*, *A.v.*—*Aspergillus versicolor*, *A.o.*—*Aspergillus ochraceus*, *A.n.*—*Aspergillus niger*, *T.v.*—*Trichoderma viride*, *P.f.*—*Penicillium funiculosum*, *P.o.*—*Penicillium ochrochloron* and *P.v.c.*—*Penicillium verrucosum* var. *cyclopium*.

**Table 3:** Molecular docking on antifungal targets.

N/N	Est. Binding Energy (kcal/mol)		I-H	Residues CYP51 of *C. albicans*	Interactions with HEM601
	DNA Topoisomerase IV 1S16	CYP51 5V5Z			
**3a**	−2.15	−8.74	1	Tyr132	Hydrophobic
**3b**	-	−8.52	1	Tyr132	Hydrophobic
**3c**	−1.82	−8.97	1	Tyr135	Hydrophobic, aromatic
**3d**	-	−8.95	1	Tyr145	Hydrophobic, aromatic
**3e**	−1.20	−8.66	1	Tyr132	Hydrophobic
**3f**	-	−8.62	1	Tyr145	Hydrophobic
**3g**	−4.10	−9.12	2	Tyr145, Tyr132	Hydrophobic, aromatic
**3h**	−3.62	−8.83	1	Tyr132	Hydrophobic, aromatic
**3i**	−2.73	−8.84	1	Tyr132	Hydrophobic
**3j**	−1.28	−8.25	1	Tyr132	Hydrophobic
**3k**	-	−9.56	-	-	Hydrophobic, Fe binding
**3l**	-	−8.37	1	Tyr132	Hydrophobic
**3m**	−2.03	−9.02	1	Tyr145	Hydrophobic, aromatic
**3n**	−2.56	−9.51	-	-	Hydrophobic, **Fe binding**
**Ketoconazole**	-	−8.23	1	Tyr64	Hydrophobic, aromatic

**Table 4:** Drug-likeness predictions and physicochemical/pharmacokinetic/ADME properties of tested compounds.

No	MW	Number of HBA ^a^	Number of HBD ^b^	Log *P*_o/w_ (iLOGP) ^c^	Log S ^d^	TPSA ^e^	BBB Permeant ^f^	Lipinski, Ghose, Veber, Egan, and Muegge Violations	Bioavailability Score	Drug-Likeness Model Score
**3a**	369.01	4	1	2.78	Moderately soluble	60.17	Yes	0	0.55	−0.56
**3b**	325.06	4	1	2.67	Moderately soluble	60.17	Yes	0	0.55	−0.23
**3c**	325.75	4	1	2.51	Moderately soluble	60.17	Yes	0	0.55	−0.54
**3d**	360.19	4	1	2.77	Moderately soluble	60.17	Yes	0	0.55	−0.44
**3e**	305.33	4	1	2.53	Moderately soluble	60.17	Yes	0	0.55	−0.27
**3f**	394.64	4	1	3.04	Poorly soluble	60.17	Yes	0	0.55	−0.53
**3g**	439.09	4	1	3.15	Poorly soluble	60.17	Yes	0	0.55	−0.29
**3h**	405.19	6	1	2.38	Moderately soluble	105.99	No	0	0.55	−0.60
**3i**	436.29	4	1	3.46	Poorly soluble	60.17	Yes	1	0.55	−0.26
**3j**	347.41	4	1	3.08	Moderately soluble	60.17	Yes	0	0.55	0.29
**3k**	271.31	4	1	2.02	Soluble	60.17	Yes	0	0.55	0.29
**3l**	316.31	6	1	1.85	Soluble	105.99	No	0	0.55	0.29
**3m**	305.76	4	1	2.25	Moderately soluble	60.17	Yes	0	0.55	−0.63
**3n**	350.21	4	1	2.38	Moderately soluble	60.17	Yes	0	0.55	0.29

^a^ number of hydrogen bond acceptors; ^b^ number of hydrogen bond donors; ^c^ lipophilicity; ^d^ water solubility (SILICOS-IT (S—soluble)); ^e^ topological polar surface area (Å^2^); ^f^ blood–brain barrier permeability.

## Data Availability

Not applicable.

## Supplementary Materials

The following are available online: The copies of ^1^H NMR and ^13^C NMR spectra for all new synthesized compounds.

## Sample Availability

Not available.

## References

[1] Maertens J. (2004). History of the development of azole derivatives. Clin. Microbiol. Infect..

[2] Mast N., Zheng W., Stout C.D., Pikuleva I. (2013). Antifungal Azoles: Structural Insights into Undesired Tight Binding to Cholesterol-Metabolizing CYP46A1. Mol. Pharmacol..

[3] Zavrel M., Esquivel B.D., White T.C. (2017). The Ins and Outs of Azole Antifungal Drug Resistance: Molecular Mechanisms of Transport. Handbook of Antimicrobial Resistance.

[4] Smith E.B., Henry J.C. (1984). Ketoconazole: An Orally Effective Antifungal Agent Mechanism of Action, Pharmacology, Clinical Efficacy and Adverse Effects. Pharmacother. J. Hum. Pharmacol. Drug Ther..

[5] Korting H.C., Schöllmann C. (2009). The significance of itraconazole for treatment of fungal infections of skin, nails and mucous membranes. J. Dtsch. Dermatol. Ges..

[6] Lackner T.E., Clissold S.P. (1989). Bifonazole. Drugs.

[7] Cuenca-Estrella M., Gomez-Lopez A., Mellado E., Garcia-Effron G., Rodriguez-Tudela J.L. (2004). In Vitro Activities of Ravuconazole and Four Other Antifungal Agents against Fluconazole-Resistant or -Susceptible Clinical Yeast Isolates. Antimicrob. Agents Chemother..

[8] Johnson L.B., Kauffman C.A. (2003). Voriconazole: A New Triazole Antifungal Agent. Clin. Infect. Dis..

[9] Greer N.D. (2003). Voriconazole: The Newest Triazole Antifungal Agent. Baylor University Medical Center Proceedings.

[10] Brown G.D., Denning D.W., Gow N.A.R., Levitz S.M., Netea M.G., White T.C. (2012). Hidden Killers: Human Fungal Infections. Sci. Transl. Med..

[11] Chen A., Sobel J.D. (2005). Emerging azole antifungals. Expert Opin. Emerg. Drugs.

[12] Cowen L., Sanglard D., Howard S.J., Rogers P.D., Perlin D.S. (2015). Mechanisms of Antifungal Drug Resistance. Cold Spring Harb. Perspect. Med..

[13] Marichal P., Bossche H.V. (1995). Mechanisms of resistance to azole antifungals. Acta Biochim. Pol..

[14] Yu S., Chai X., Wang Y., Cao Y., Zhang J., Wu Q., Zhang D., Jiang Y., Yan T., Sun Q. (2014). Triazole derivatives with improved in vitro antifungal activity over azole drugs. Drug Des. Devel. Ther..

[15] Cao X., Sun Z., Cao Y., Wang R., Cai T., Chu W., Hu W., Yang Y. (2014). Design, Synthesis, and Structure–Activity Relationship Studies of Novel Fused Heterocycles-Linked Triazoles with Good Activity and Water Solubility. J. Med. Chem..

[16] Gupta D., Jain D.K. (2015). Synthesis, antifungal and antibacterial activity of novel 1,2,4-triazole derivatives. J. Adv. Pharm. Technol. Res..

[17] Wu W., Jiang Y., Fei Q., Du H., Yang M. (2019). Synthesis and antifungal activity of novel 1,2,4-triazole derivatives containing an amide moiety. J. Heterocycl. Chem..

[18] Sadeghpour H., Khabnadideh S., Zomorodian K., Pakshir K., Hoseinpour K., Javid N., Faghih-Mirzaei E., Rezaei Z. (2017). Design, Synthesis, and Biological Activity of New Triazole and Nitro-Triazole Derivatives as Antifungal Agents. Molecules.

[19] Jin R., Liu J., Zhang G., Li J., Zhang S., Guo H. (2018). Design, Synthesis, and Antifungal Activities of Novel 1,2,4-Triazole Schiff Base Derivatives. Chem. Biodivers..

[20] Gao F., Wang T., Xiao J., Huang G. (2019). Antibacterial activity study of 1,2,4-triazole derivatives. Eur. J. Med. Chem..

[21] Yang L., Bao X.-P. (2017). Synthesis of novel 1,2,4-triazole derivatives containing the quinazolinylpiperidinyl moiety and N-(substituted phenyl)acetamide group as efficient bactericides against the phytopathogenic bacterium *Xanthomonas oryzae* pv. *oryzae*. RSC Adv..

[22] Du H., Fan Z., Yang L., Bao X. (2017). Synthesis of novel quinazolin-4(3H)-one derivatives containing the 7-oxo-1,2,4-triazolo[1,5-a]pyrimidine moiety as effective agricultural bactericides against the pathogen *Xanthomonas oryzae* pv. oryzae. Mol. Divers..

[23] Thakur A., Gupta P.S., Shukla P.K., Verma A., Pathak P. (2016). 1, 2, 4-Triazole Scafolds: Recent Advances and Pharmacological Applications. Int. J. Curr. Res. Acad. Rev..

[24] Singh R., Kashaw S.K., Mishra V.K., Mishra M., Rajoriya V., Kashaw V. (2018). Design and Synthesis of New Bioactive 1,2,4-Triazoles, Potential Antitubercular and Antimicrobial Agents. Indian J. Pharm. Sci..

[25] Papadopoulou M.V., Bloomer W.D., Rosenzweig H.S., Chatelain E., Kaiser M., Wilkinson S.R., McKenzie C., Ioset J.-R. (2012). Novel 3-Nitro-1H-1,2,4-triazole-Based Amides and Sulfonamides as Potential Antitrypanosomal Agents. J. Med. Chem..

[26] Papadopoulou M.V., Bloomer W.D., Lepesheva G.I., Rosenzweig H.S., Kaiser M., Aguilera-Venegas B., Wilkinson S.R., Chatelain E., Ioset J.-R. (2015). Novel 3-Nitrotriazole-Based Amides and Carbinols as Bifunctional Antichagasic Agents. J. Med. Chem..

[27] Chu X.-M., Wang C., Wang W.-L., Liang L., Liu W., Gong K.-K., Sun K.L. (2019). Triazole derivatives and their antiplasmodial and antimalarial activities. Eur. J. Med. Chem..

[28] Li Z., Cao Y., Zhan P., Pannecouque C., Balzarini J., De Clercq E., Liu X. (2012). Synthesis and Anti-HIV Evaluation of Novel 1,2,4-triazole Derivatives as Potential Non-nucleoside HIV-1 Reverse Transcriptase Inhibitors. Lett. Drug Des. Discov..

[29] Wu G., Gao Y., Kang D., Huang B., Huo Z., Liu H., Poongavanam V., Zhan P., Liu X. (2017). Design, synthesis and biological evaluation of tacrine-1,2,3-triazole derivatives as potent cholinesterase inhibitors. MedChemComm.

[30] Li Y., Pasunooti K.K., Li R.-J., Liu W., Head S.A., Shi W.Q., Liu J.O. (2018). Novel Tetrazole-Containing Analogues of Itraconazole as Potent Antiangiogenic Agents with Reduced Cytochrome P450 3A4 Inhibition. J. Med. Chem..

[31] El-Sherief H.A., Youssif B.G., Bukhari S.N.A., Abdel-Aziz M., Abdel-Rahman H. (2018). Novel 1,2,4-triazole derivatives as potential anticancer agents: Design, synthesis, molecular docking and mechanistic studies. Bioorg. Chem..

[32] Mahanti S., Sunkara S., Bhavani R. (2019). Synthesis, biological evaluation and computational studies of fused acridine containing 1,2,4-triazole derivatives as anticancer agents. Synth. Commun..

[33] Mohamed M.A.A., Allah O.A.A., Bekhit A.A., Kadry A.M., El-Saghier A.M.M. (2020). Synthesis and antidiabetic activity of novel triazole derivatives containing amino acids. J. Heterocycl. Chem..

[34] Ye G.-J., Lan T., Huang Z.-X., Cheng X.-N., Cai C.-Y., Ding S.-M., Xie M.-L., Wang B. (2019). Design and synthesis of novel xanthone-triazole derivatives as potential antidiabetic agents: α-Glucosidase inhibition and glucose uptake promotion. Eur. J. Med. Chem..

[35] Sari S., Kaynak F.B., Dalkara S. (2018). Synthesis and anticonvulsant screening of 1,2,4-triazole derivatives. Pharmacol. Rep..

[36] Xia Y., Chen C., Liu Y., Ge G., Dou T., Wang P. (2018). Synthesis and Structure-Activity Relationship of Daphnetin Derivatives as Potent Antioxidant Agents. Molecules.

[37] Mashhadinezhad M., Mamaghani M., Rassa M., Shirini F. (2019). A Facile Green Synthesis of Chromene Derivatives as Antioxidant and Antibacterial Agents through a Modified Natural Soil. Chem..

[38] Gandhi D., Agarwal D.K., Kalal P., Bhargava A., Jangid D., Agarwal S. (2018). Synthesis, characterization and evaluation of novel benzothiazole clubbed chromene derivatives for their anti-inflammatory potential. Phosphorus Sulfur Silicon Relat. Elem..

[39] Chougala B.M., Samundeeswari S., Holiyachi M., Naik N.S., Shastri L.A., Dodamani S., Jalalpure S., Dixit S.R., Joshi S.D., Sunagar V.A. (2018). Green, unexpected synthesis of bis-coumarin derivatives as potent anti-bacterial and anti-inflammatory agents. Eur. J. Med. Chem..

[40] Youssef M.S.K., Abeed A.A.O., El-Emary T.I. (2017). Synthesis and evaluation of chromene-based compounds containing pyrazole moiety as antimicrobial agents. Heterocycl. Commun..

[41] Khan M.S., Agrawal R., Ubaidullah M., Hassan I., Tarannum N., Ubaidullah M. (2019). Design, synthesis and validation of anti-microbial coumarin derivatives: An efficient green approach. Heliyon.

[42] Bhagat K., Bhagat J., Gupta M.K., Singh J.V., Gulati H.K., Singh A., Kaur K., Kaur G., Sharma S., Rana A. (2019). Design, Synthesis, Antimicrobial Evaluation, and Molecular Modeling Studies of Novel Indolinedione–Coumarin Molecular Hybrids. ACS Omega.

[43] Prusty J.S., Kumar A. (2020). Coumarins: Antifungal effectiveness and future therapeutic scope. Mol. Divers..

[44] Fouad S.A., Hessein S.A., Abbas S.Y., Farrag A., Ammar Y.A. (2018). Synthesis of Chromen-2-one, Pyrano[3,4-c]chromene and Pyridino[3,4-c]chromene Derivatives as Potent Antimicrobial Agents. Croat. Chem. Acta.

[45] Kushwaha K., Kaushik N., Lata, Jain S.C. (2014). Design and synthesis of novel 2H-chromen-2-one derivatives bearing 1,2,3-triazole moiety as lead antimicrobials. Bioorg. Med. Chem. Lett..

[46] Al-Masoudi N.A., Mohammed H.H., Hamdy A.M., Akrawi O.A., Eleya N., Spannenberg A., Pannecouque C., Langer P. (2013). Synthesis and anti-HIV Activity of New Fused Chromene Derivatives Derived from 2-Amino-4-(1-naphthyl)-5-oxo-4H,5H-pyrano[3,2- c]chromene-3-carbonitrile. Z. Für Nat. B.

[47] Jesumoroti O.J., Faridoon F., Mnkandhla D., Isaacs M., Hoppe H.C., Klein R. (2019). Evaluation of novel N′-(3-hydroxybenzoyl)-2-oxo-2*H*-chromene-3-carbohydrazide derivatives as potential HIV-1 integrase inhibitors. MedChemComm.

[48] Datar P., Auti P.B. (2016). Design and synthesis of novel 4-substituted 1,4-dihydropyridine derivatives as hypotensive agents. J. Saudi Chem. Soc..

[49] Venkateswararao E., Kim M.-S., Sharma V.K., Lee K.-C., Subramanian S., Roh E., Kim Y., Jung S.-H. (2013). Identification of novel chromenone derivatives as interleukin-5 inhibitors. Eur. J. Med. Chem..

[50] Shaveta, Mishra S., Singh P. (2016). Hybrid molecules: The privileged scaffolds for various pharmaceuticals. Eur. J. Med. Chem..

[51] Bérubé G. (2016). An overview of molecular hybrids in drug discovery. Expert Opin. Drug Discov..

[52] Lödige M., Hiersch L. (2015). Design and Synthesis of Novel Hybrid Molecules against Malaria. Int. J. Med. Chem..

[53] Defant A., Vozza A., Mancini I. (2019). Design, Synthesis and Antimicrobial Evaluation of New Norfloxacin-Naphthoquinone Hybrid Molecules. Proceedings.

[54] Stingaci E., Zveaghinteva M., Pogrebnoi S., Lupascu L., Valica V., Uncu L., Smetanscaia A., Drumea M., Petrou A., Ciric A. (2020). New vinyl-1,2,4-triazole derivatives as antimicrobial agents: Synthesis, biological evaluation and molecular docking studies. Bioorganic Med. Chem. Lett..

[55] Shi Y.-L., Shi M. (2007). The synthesis of chromenes, chromanes, coumarins and related heterocycles via tandem reactions of salicylic aldehydes or salicylic imines with α,β-unsaturated compounds. Org. Biomol. Chem..

[56] Filimonov D., Lagunin A.A., Gloriozova T.A., Rudik A., Druzhilovskii D.S., Pogodin P.V., Poroikov V.V. (2014). Prediction of the Biological Activity Spectra of Organic Compounds Using the Pass Online Web Resource. Chem. Heterocycl. Compd..

[57] Poroikov V.V. (2020). Computer-Aided Drug Design: From Discovery of Novel Pharmaceutical Agents to Systems Pharmacology. Biochem. Suppl. Ser. B Biomed. Chem..

[58] Cortellis Drug Discovery Intelligence Database. https://www.cortellis.com/drugdiscovery/.

[59] Martin Y.C., Kofron A.J.L., Traphagen L.M. (2002). Do Structurally Similar Molecules Have Similar Biological Activity?. J. Med. Chem..

[60] Sheldrick G.M. (2008). A short history of SHELX. Acta Crystallogr. Sect. A Found. Crystallogr..

[61] Sheldrick G.M. (2015). Crystal structure refinement with SHELXL. Acta Crystallogr. Sect. C Struct. Chem..

[62] Amiranashvili L., Nadaraia N., Merlani M., Kamoutsis C., Petrou A., Geronikaki A., Pogodin P., Druzhilovskiy D., Poroikov V., Ciric A. (2020). Antimicrobial Activity of Nitrogen-Containing 5-α-Androstane Derivatives: In Silico and Experimental Studies. Antibiotics.

[63] Trott O., Olson A.J. (2010). AutoDock Vina: Improving the speed and accuracy of docking with a new scoring function, efficient optimization, and multithreading. J. Comput. Chem..

[64] Wolber G., Langer T. (2004). LigandScout: 3-D Pharmacophores Derived from Protein-Bound Ligands and Their Use as Virtual Screening Filters. J. Chem. Inf. Model..

[65] Egan W.J., Merz K.M., Baldwin J.J. (2000). Prediction of Drug Absorption Using Multivariate Statistics. J. Med. Chem..

[66] Ghose A.K., Viswanadhan V.N., Wendoloski J.J. (1999). A Knowledge-Based Approach in Designing Combinatorial or Medicinal Chemistry Libraries for Drug Discovery. 1. A Qualitative and Quantitative Characterization of Known Drug Databases. J. Comb. Chem..

[67] Muegge I., Heald S.L., Brittelli D. (2001). Simple Selection Criteria for Drug-like Chemical Matter. J. Med. Chem..

[68] Veber D.F., Johnson S.R., Cheng H.-Y., Smith B.R., Ward K.W., Kopple K.D. (2002). Molecular Properties That Influence the Oral Bioavailability of Drug Candidates. J. Med. Chem..

[69] Benet L.Z., Hosey C.M., Ursu O., Oprea T.I. (2016). BDDCS, the Rule of 5 and drugability. Adv. Drug Deliv. Rev..

[70] Mladěnka P., Karlíčková J., Hrubša M., Veljović E., Muratović S., Carazo A., Mali A.S., Špirtović-Halilović S., Saso L., Pour M. (2020). Interaction of 2,6,7-Trihydroxy-Xanthene-3-Ones with Iron and Copper, and Biological Effect of the Most Active Derivative on Breast Cancer Cells and Erythrocytes. Appl. Sci..

