# Supplementary figures and images for: Chromenol Derivatives as Novel Antifungal Agents: Synthesis, In Silico and In Vitro Evaluation
Source: Molecules. 2021 Jul 16;26(14):4304. doi: 10.3390/molecules26144304 (PMC8307147; doi:10.3390/molecules26144304)

3A

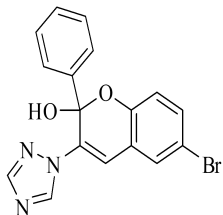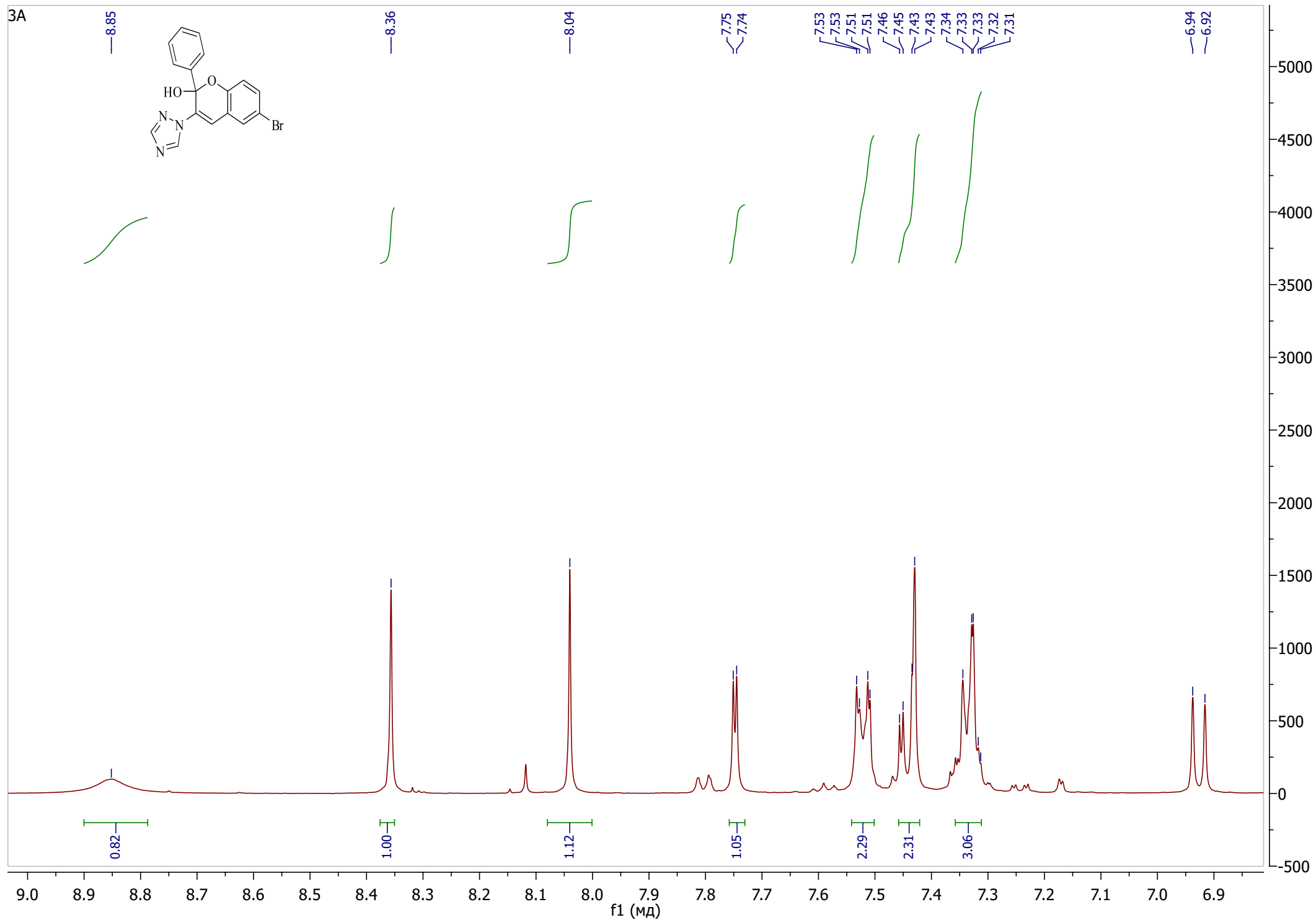

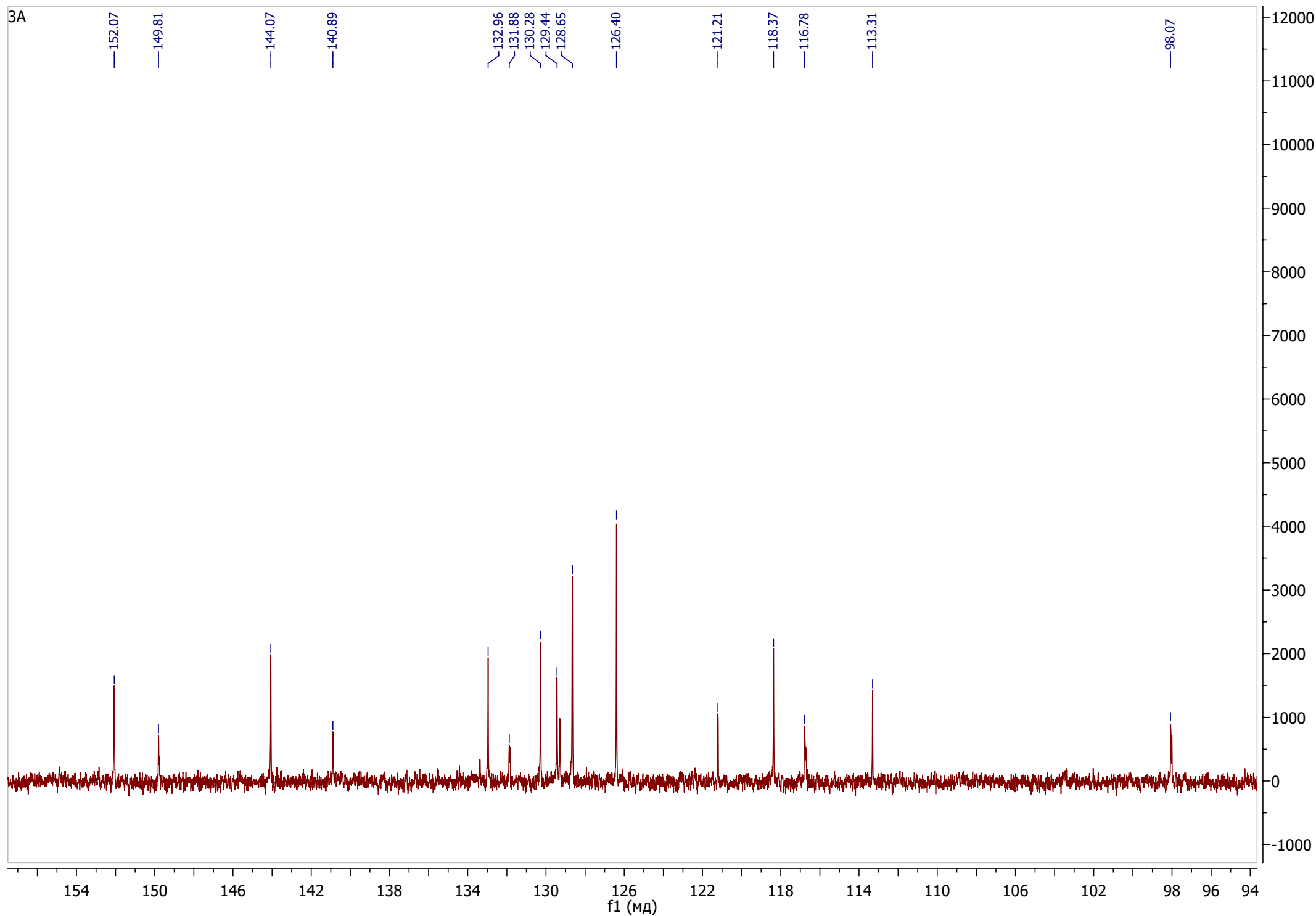

3B

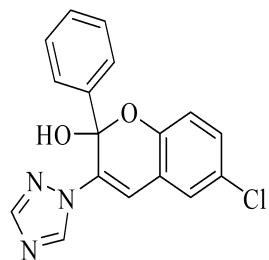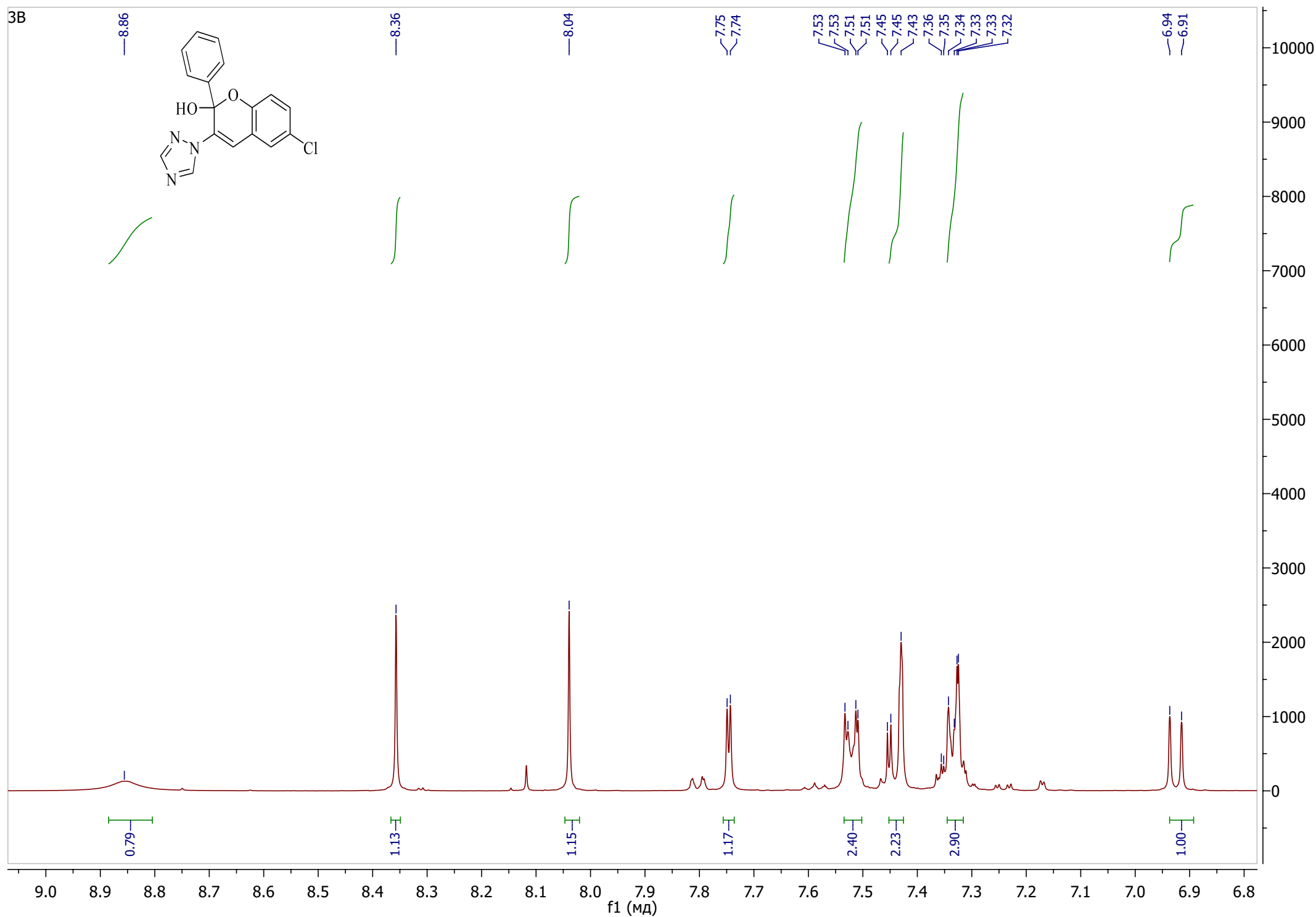

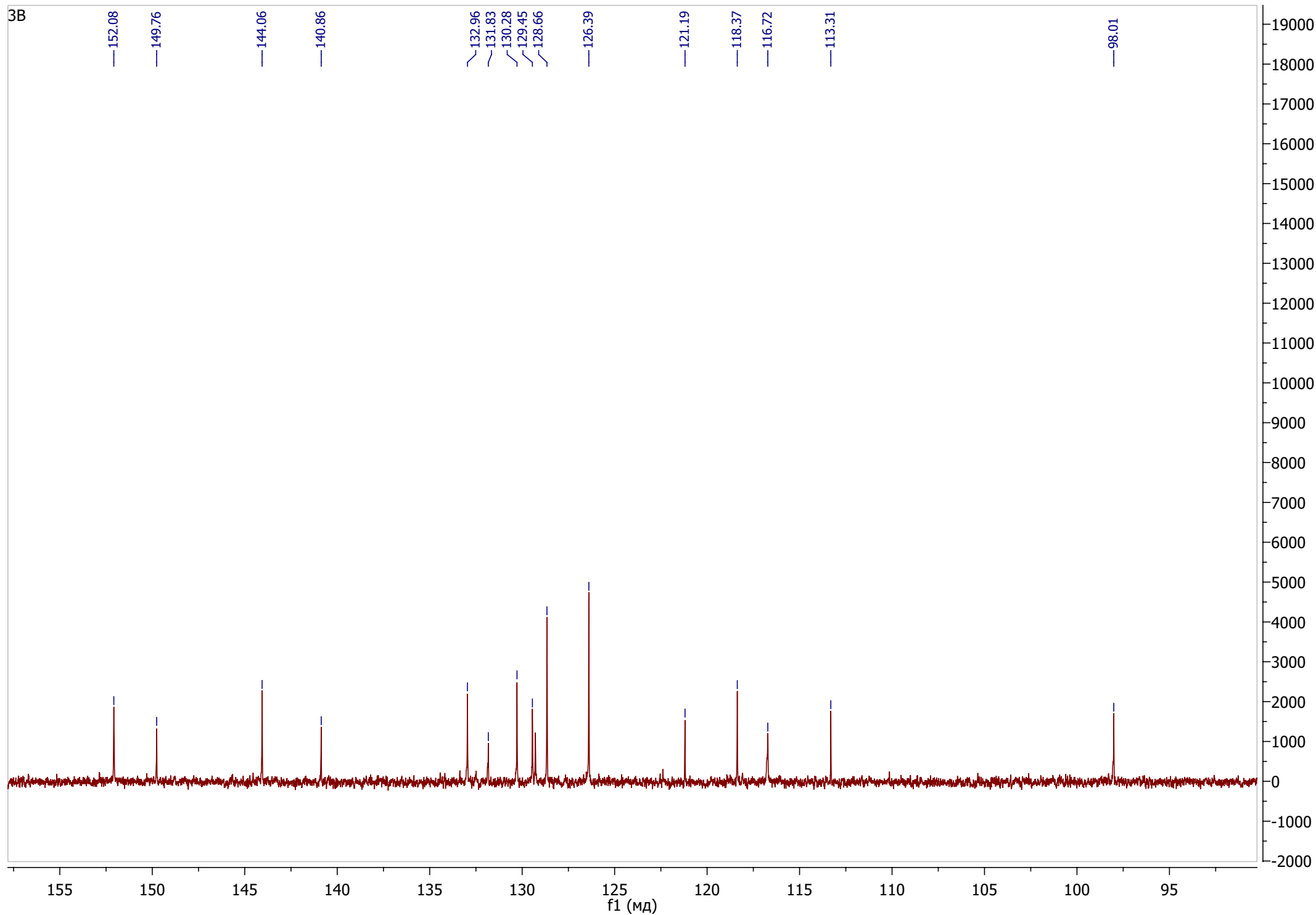

3C

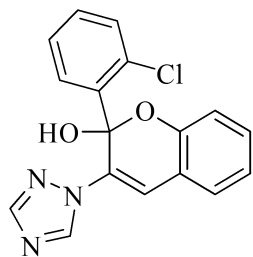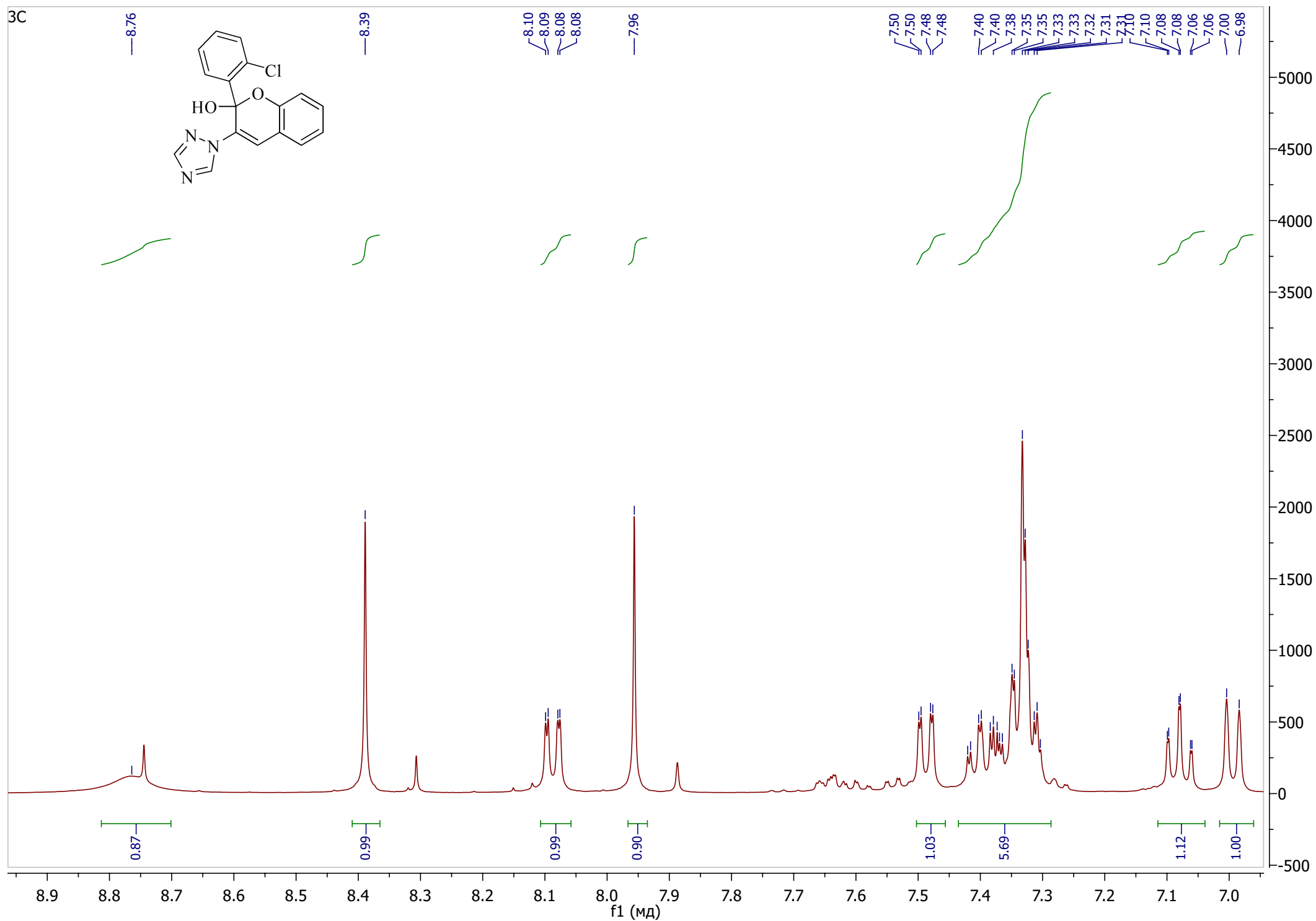

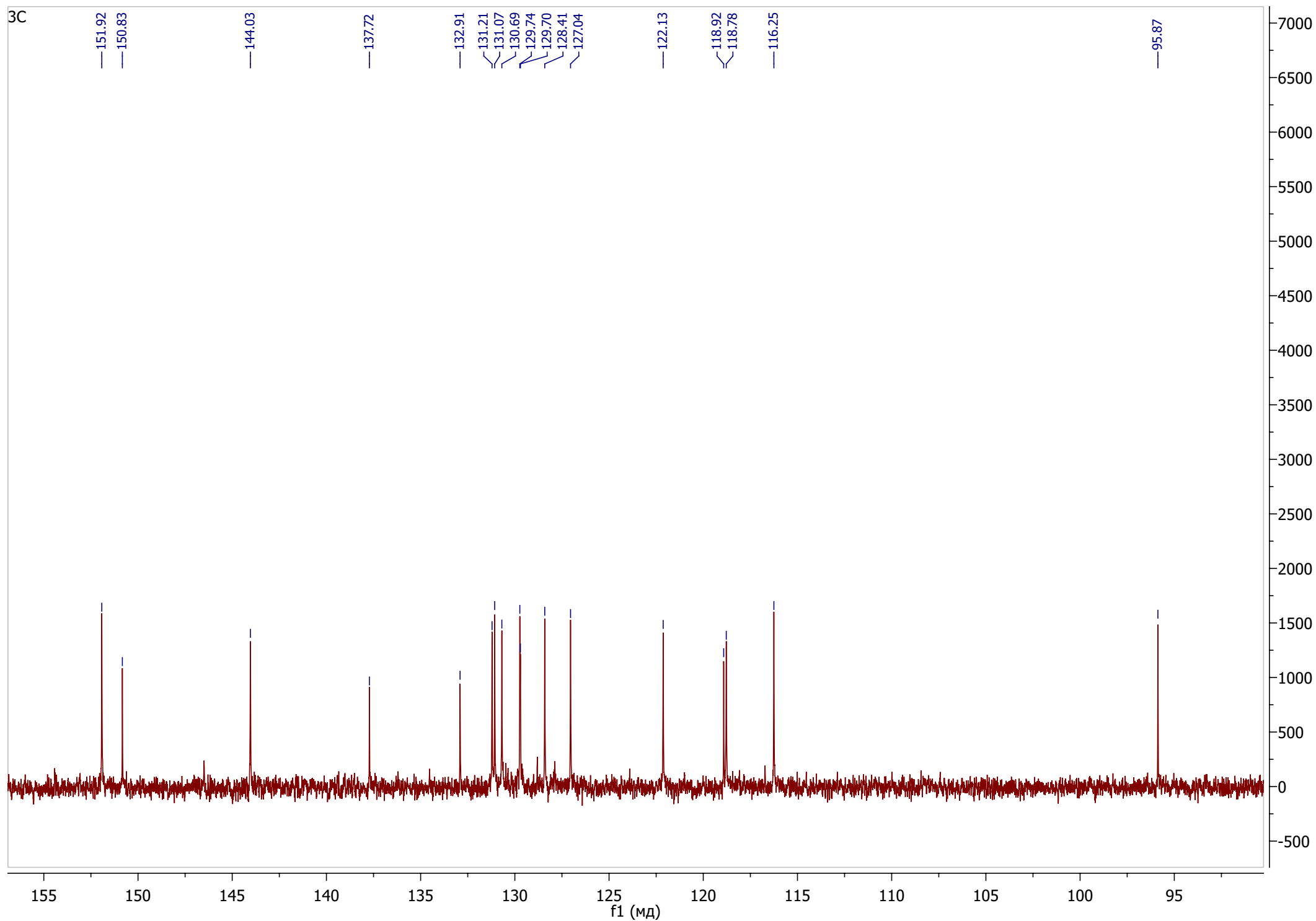

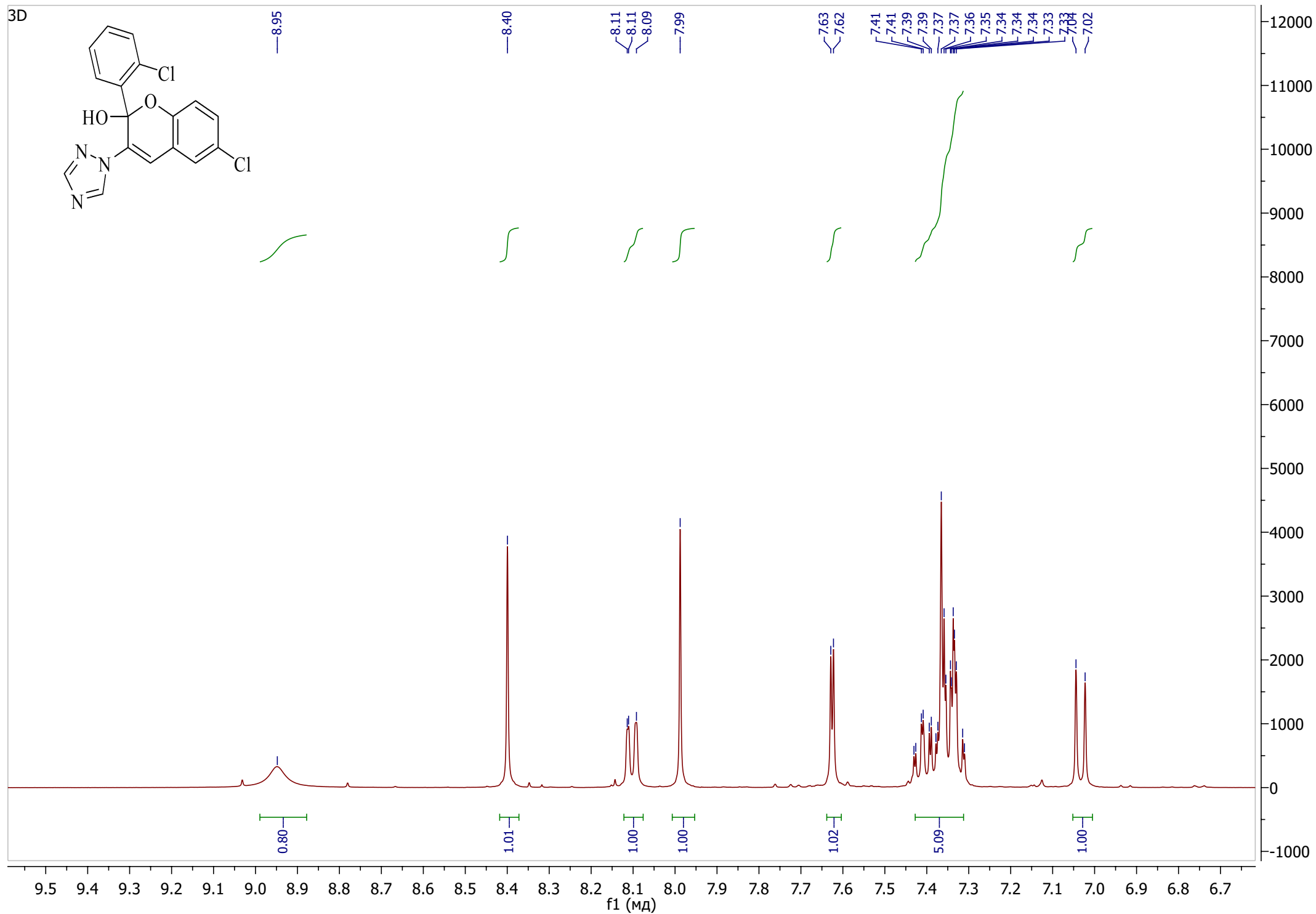

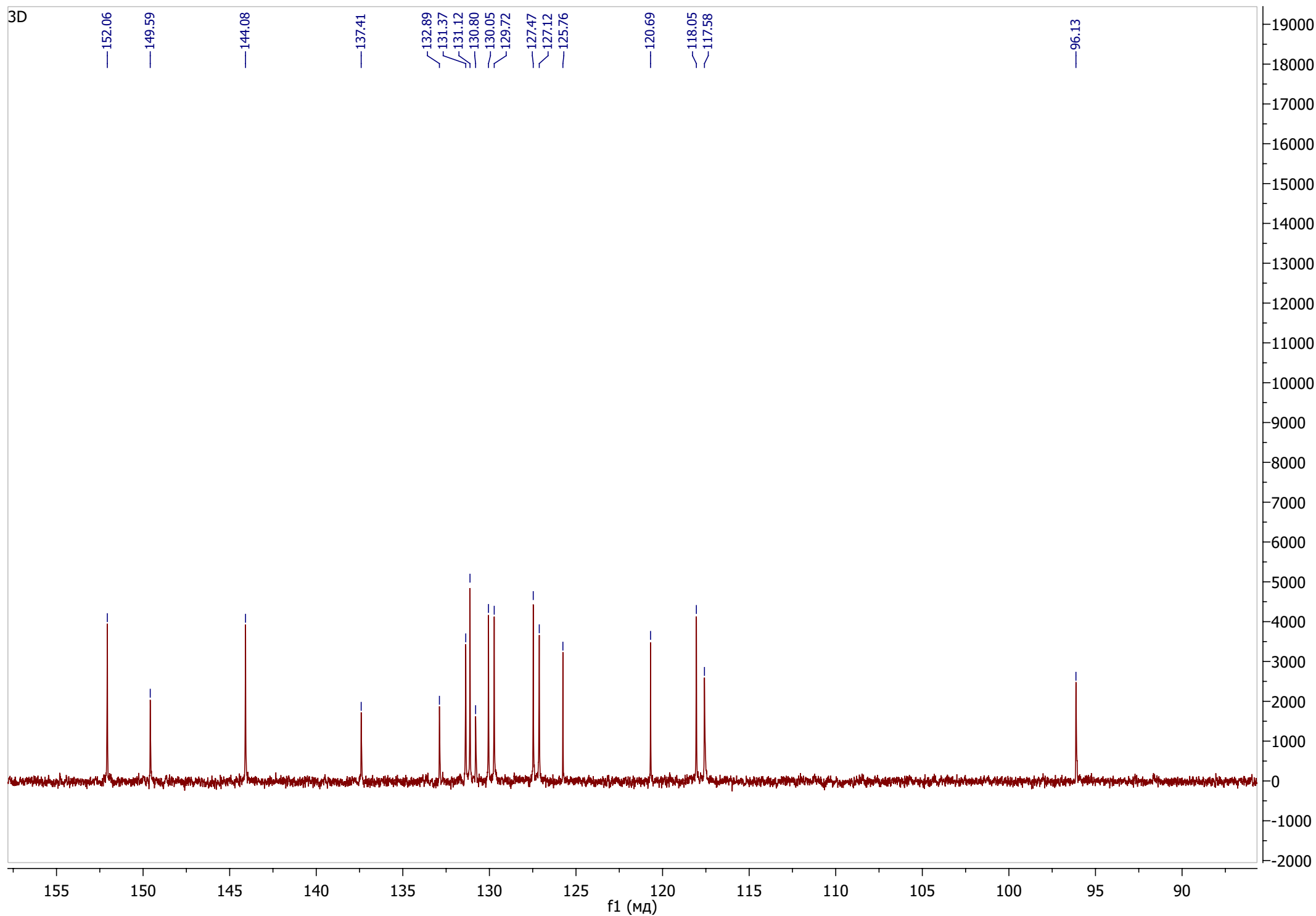

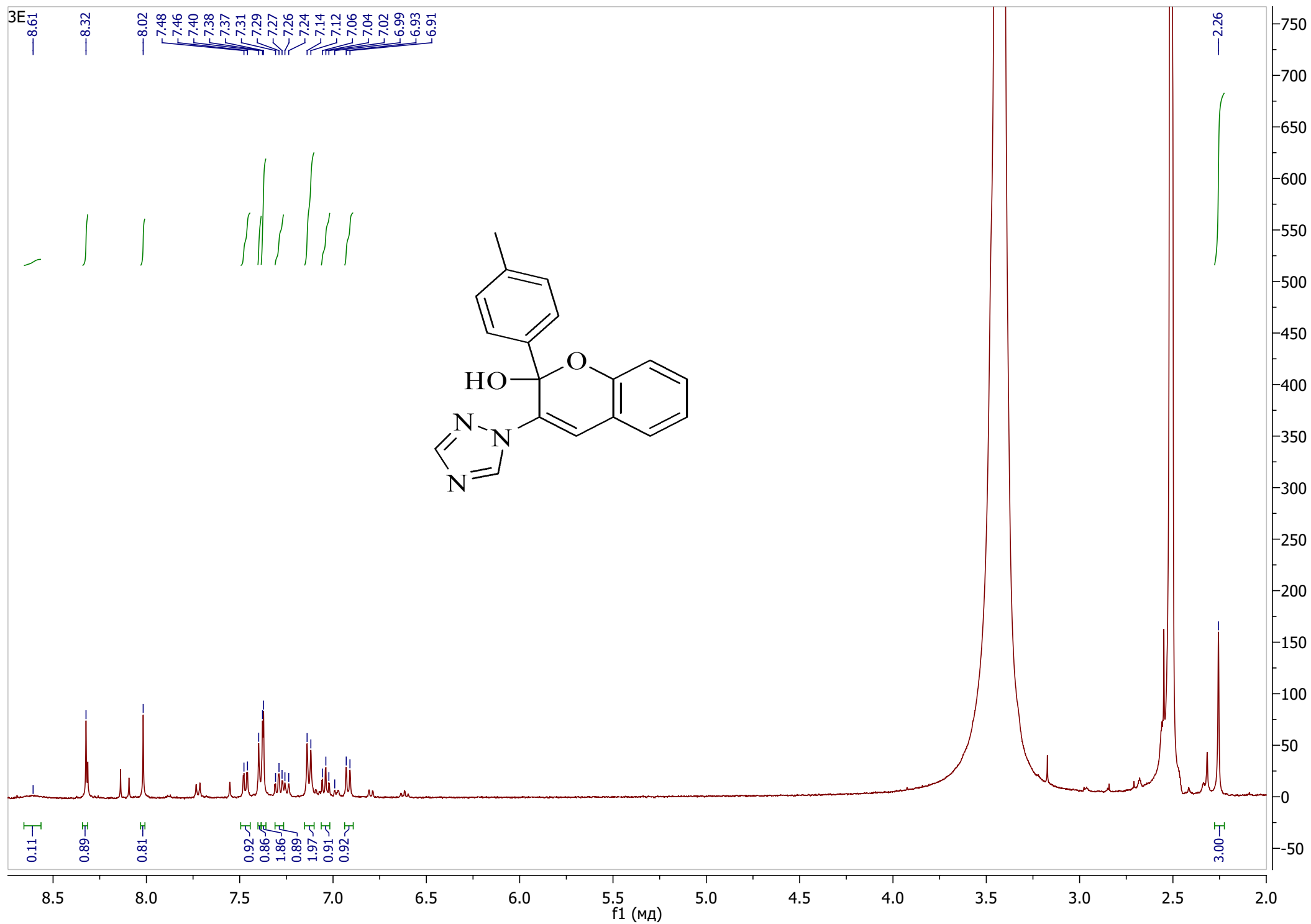

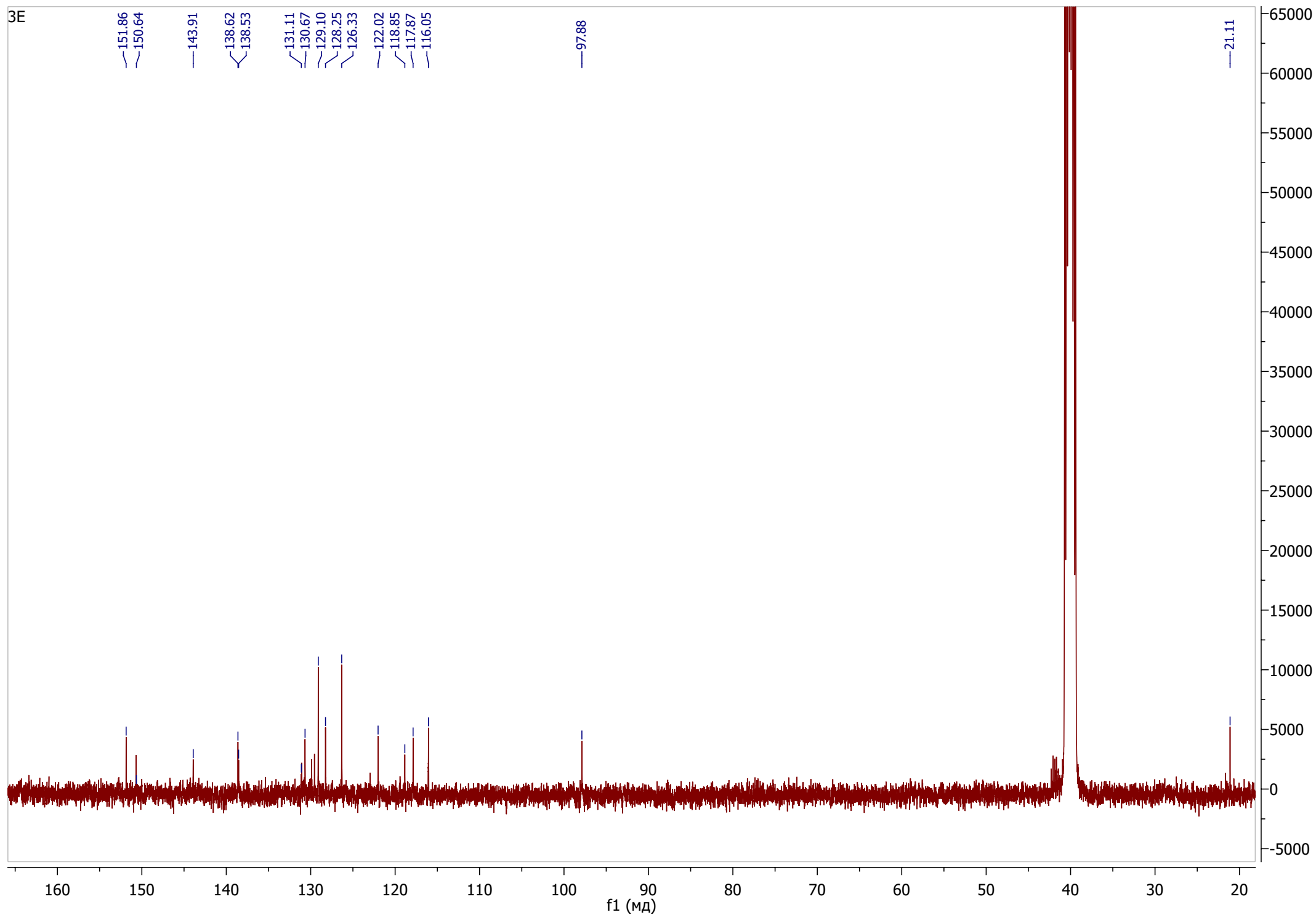

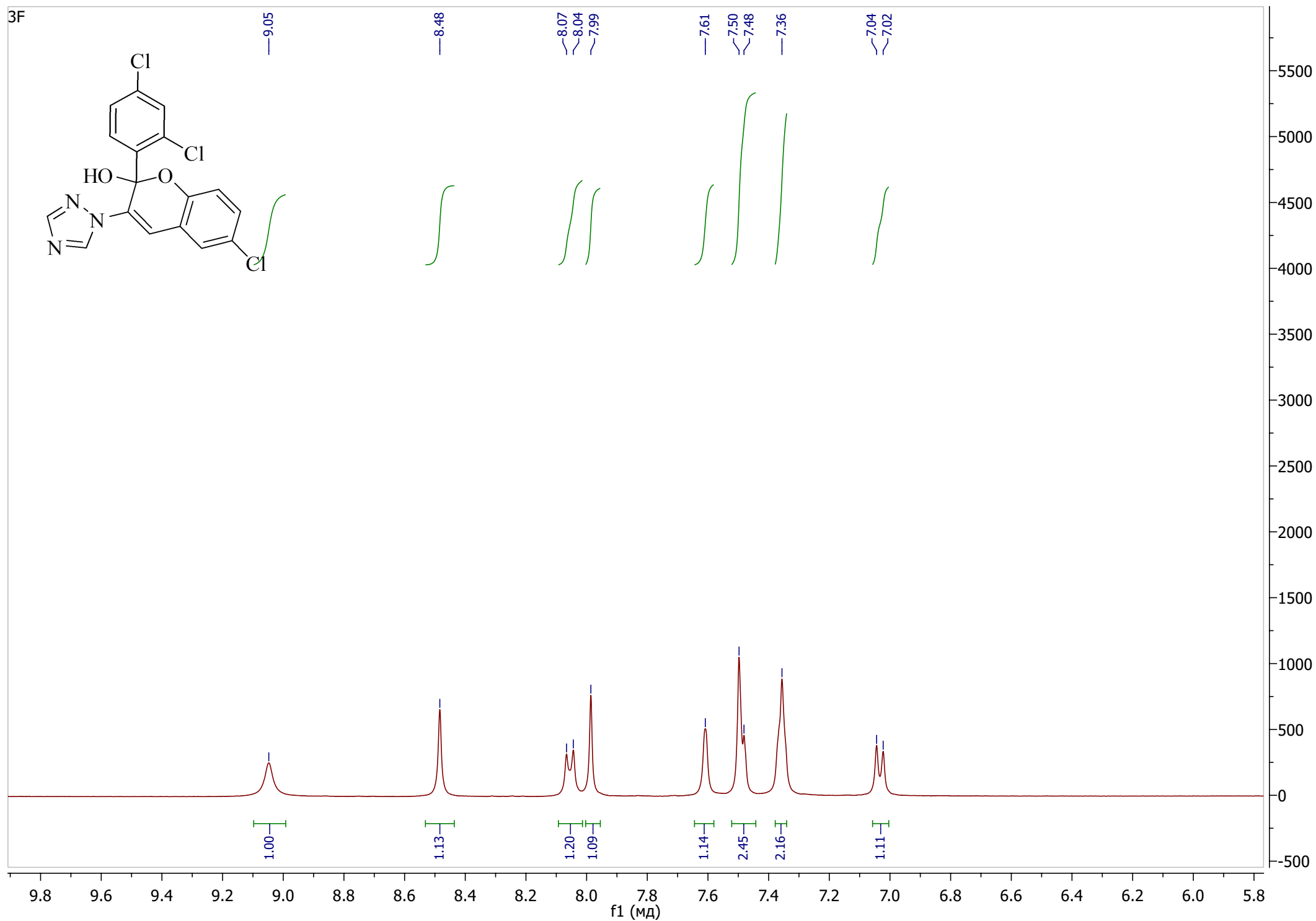

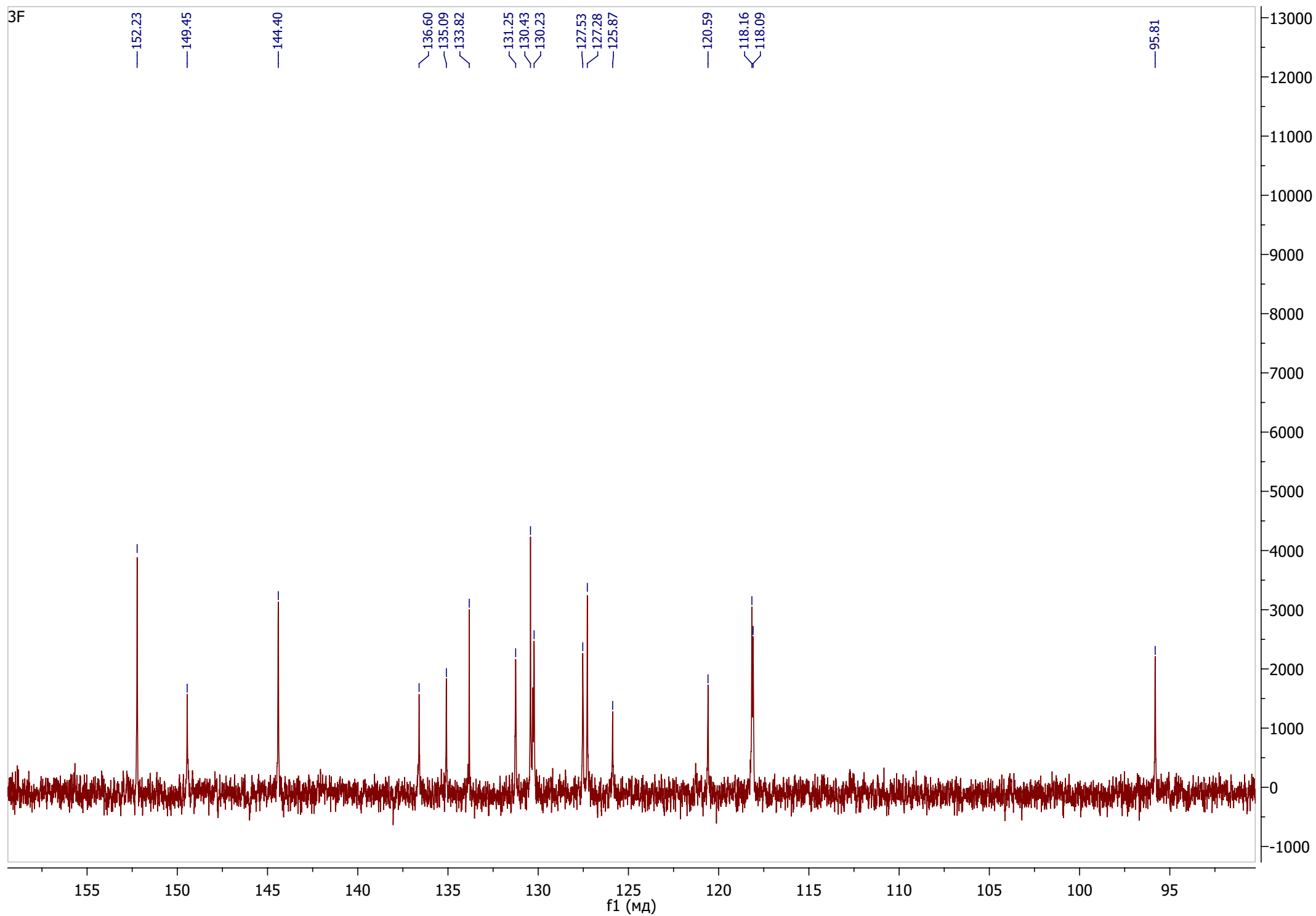

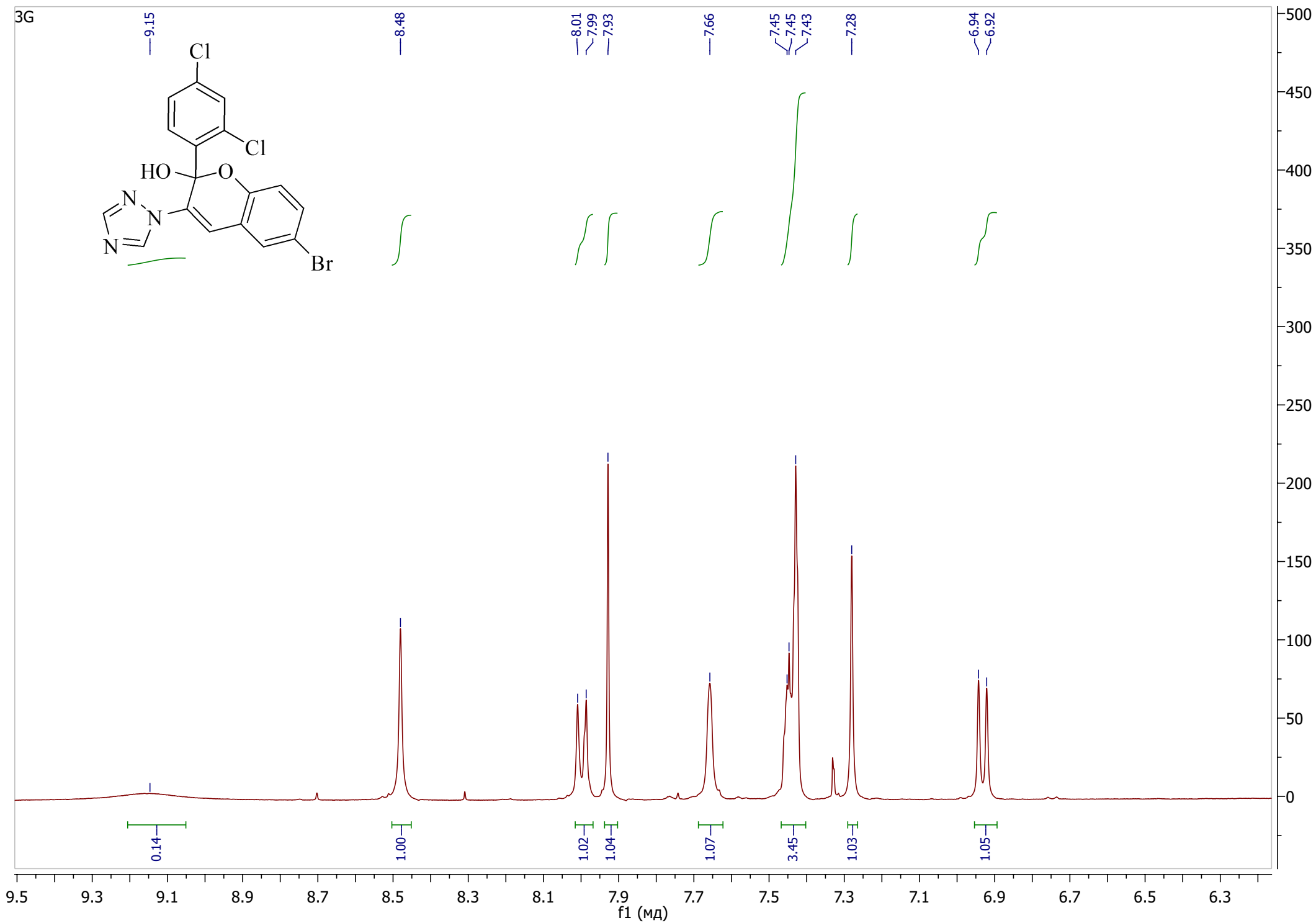

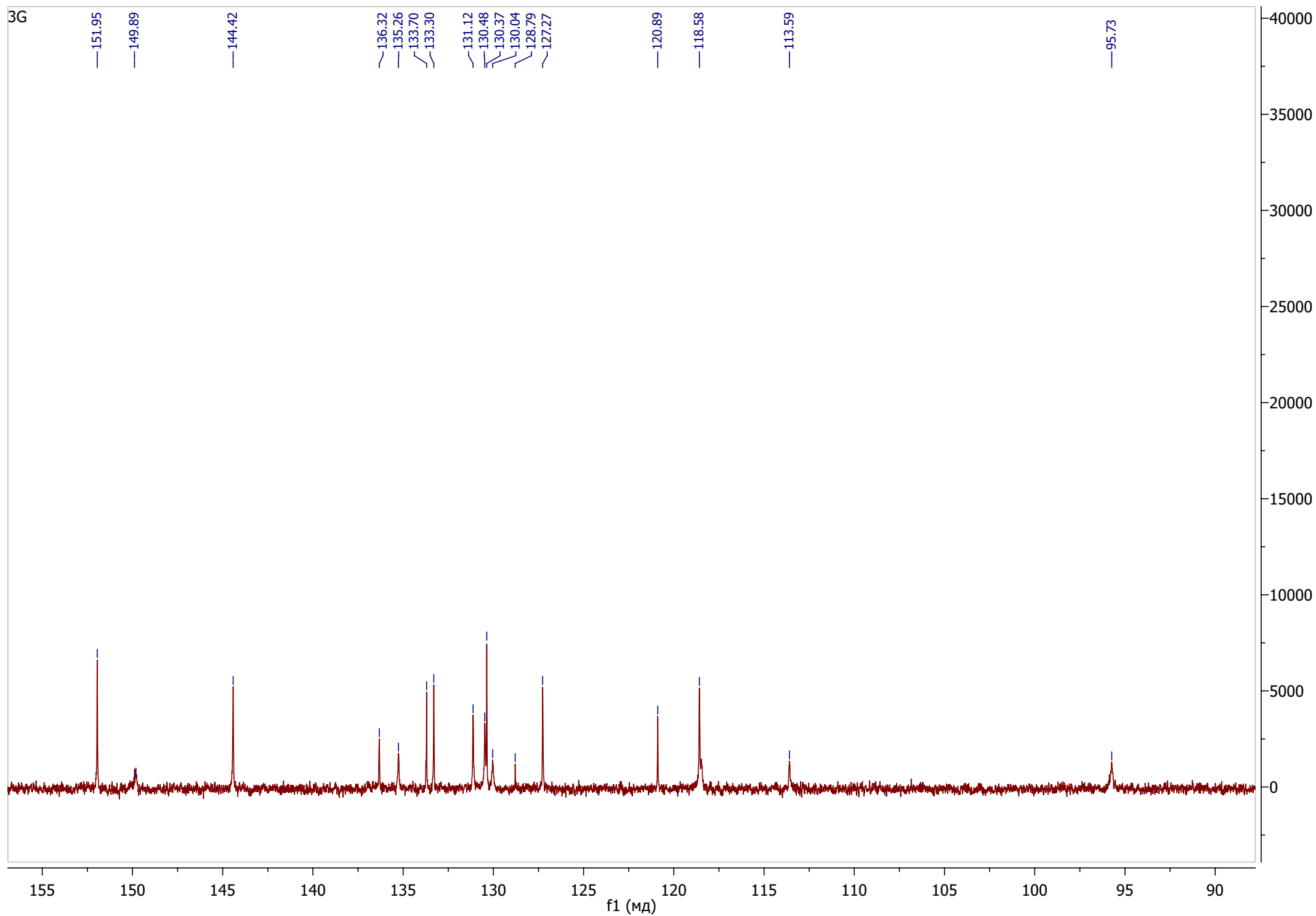

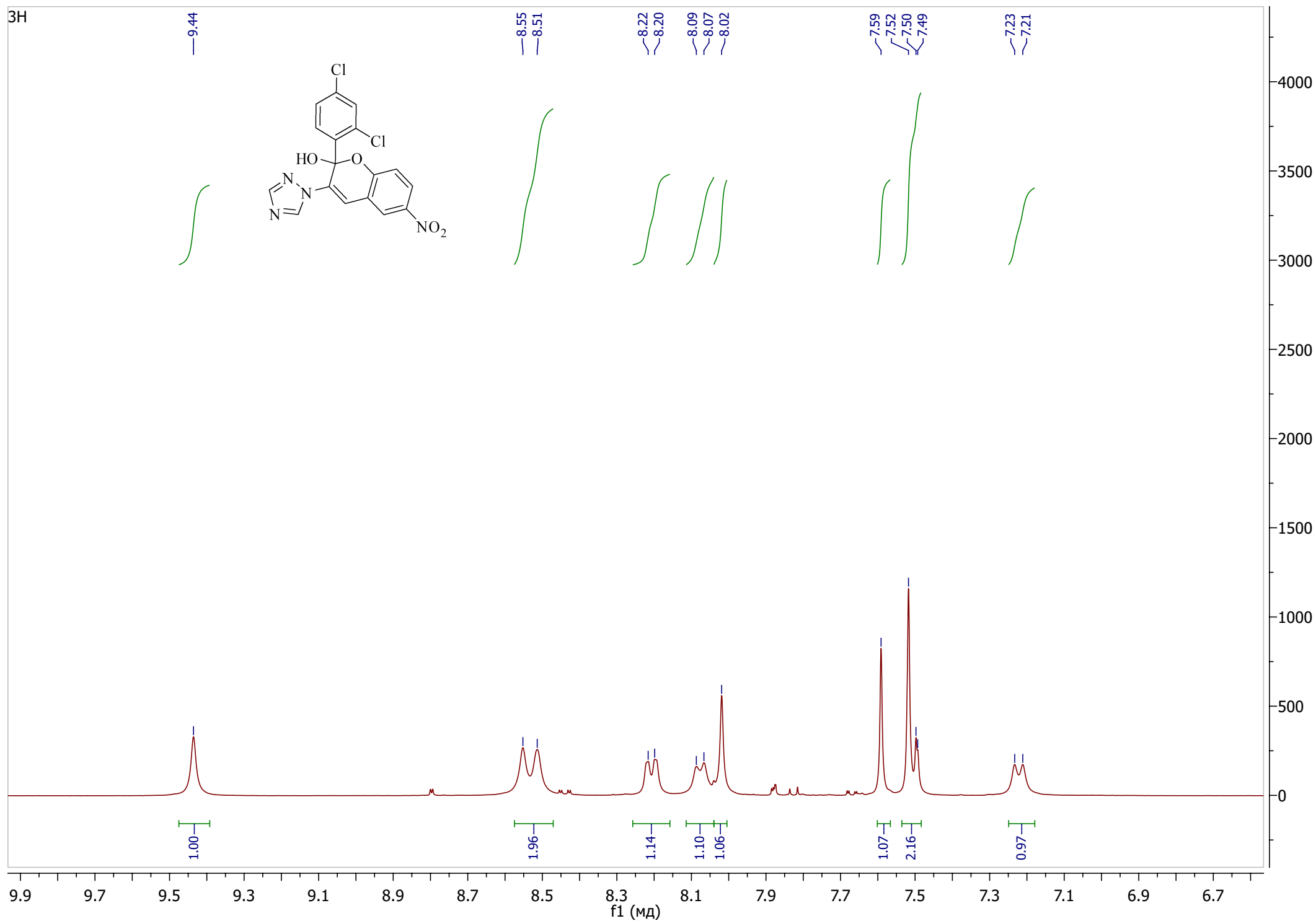

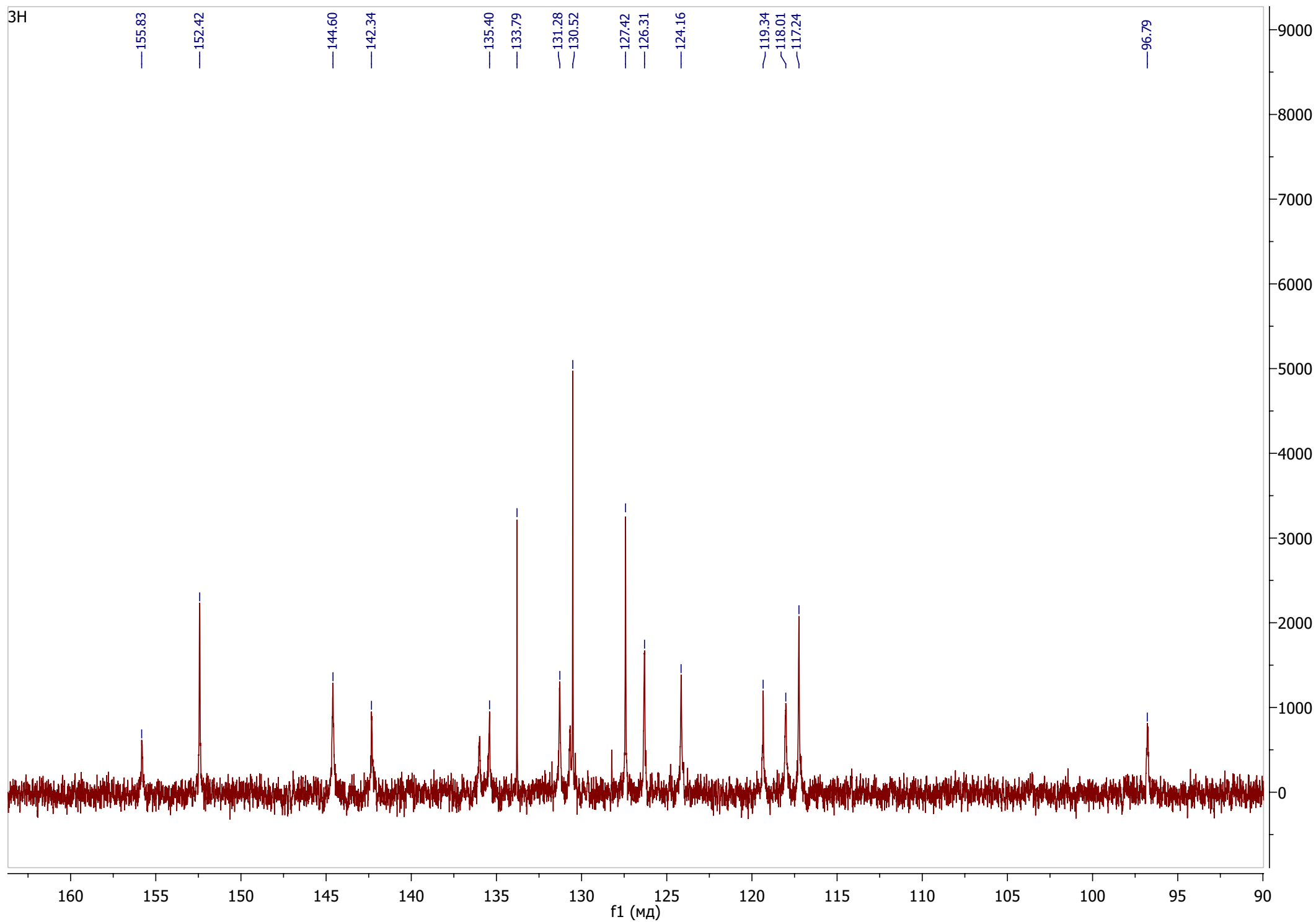

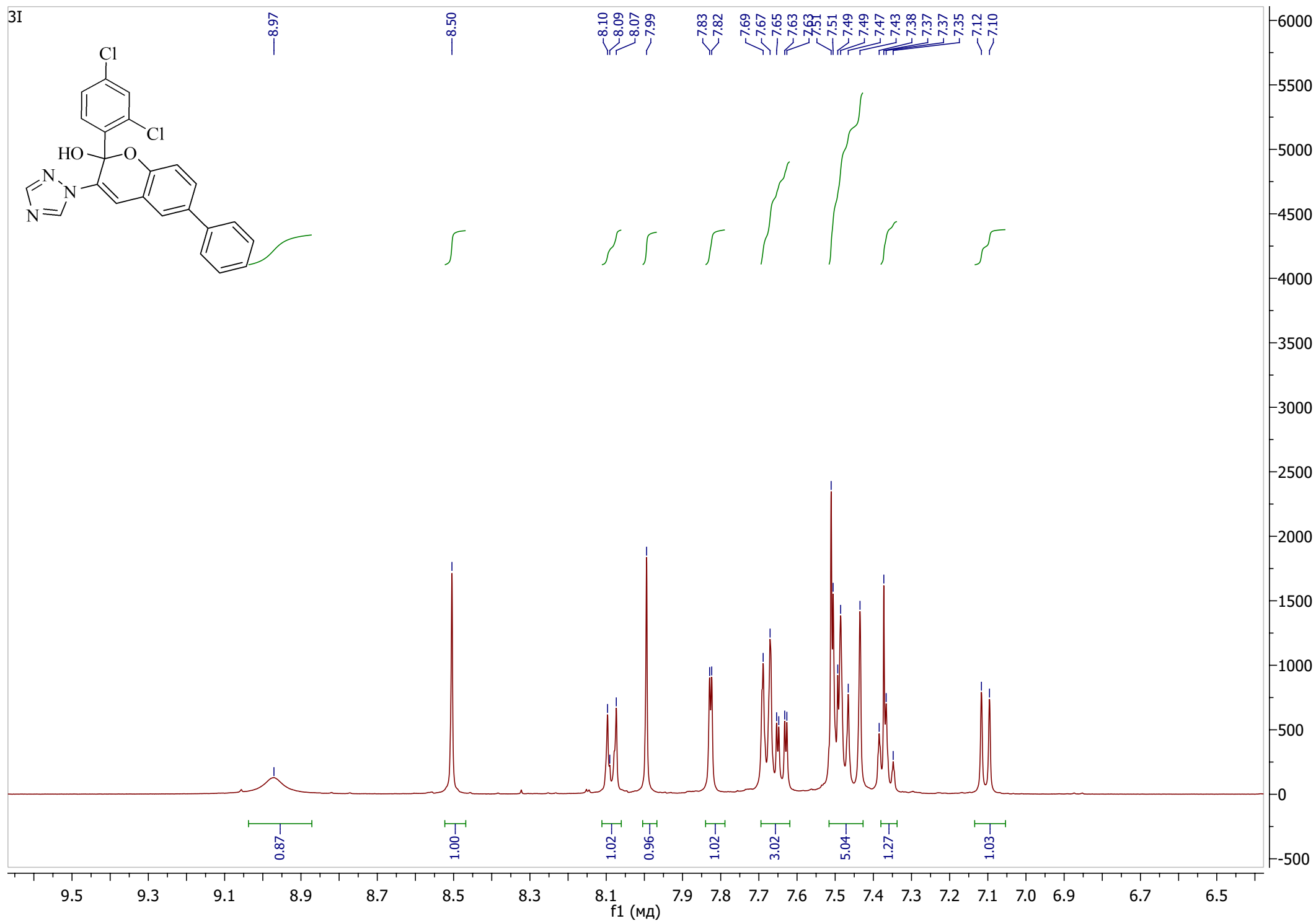

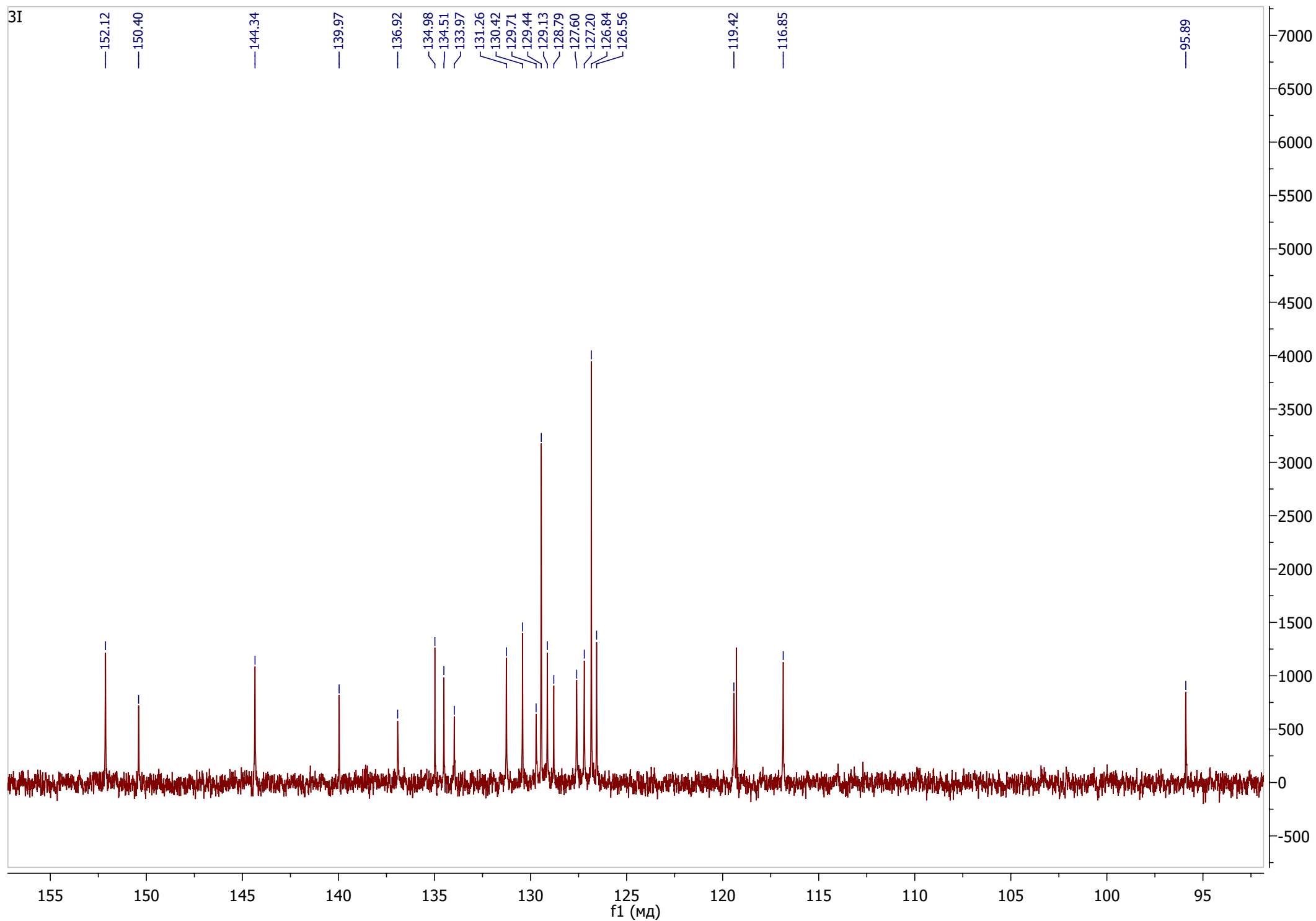

3)

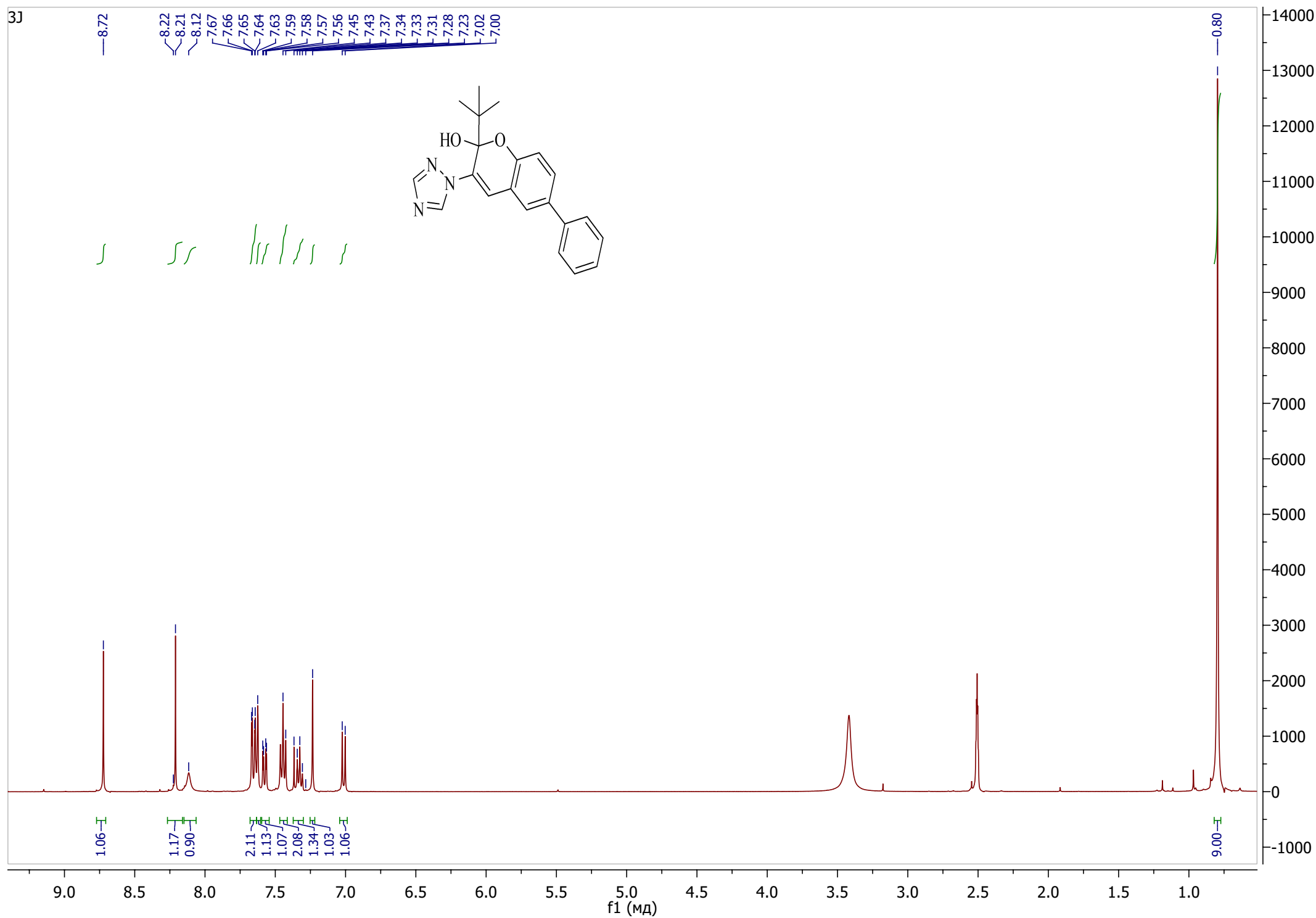

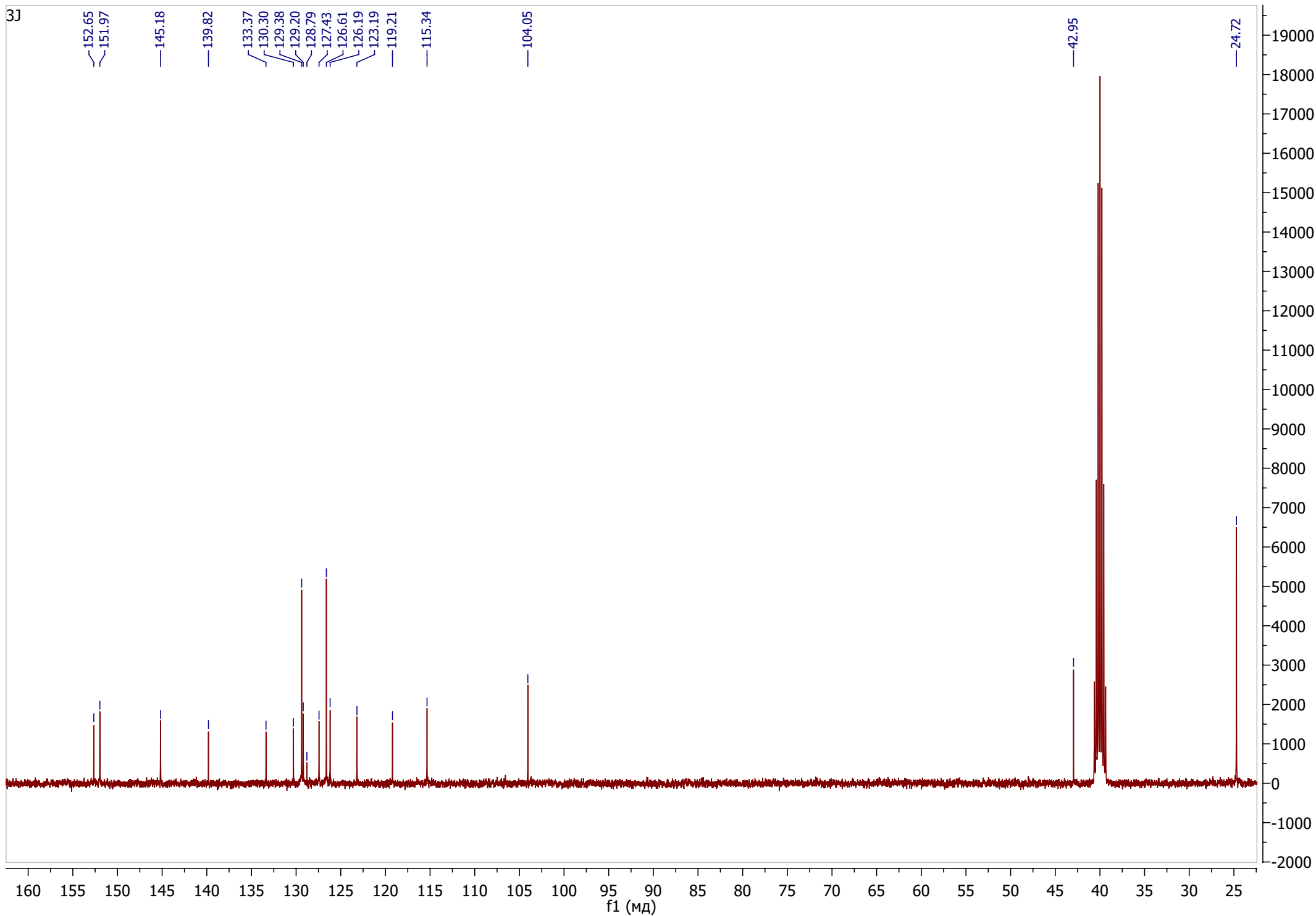

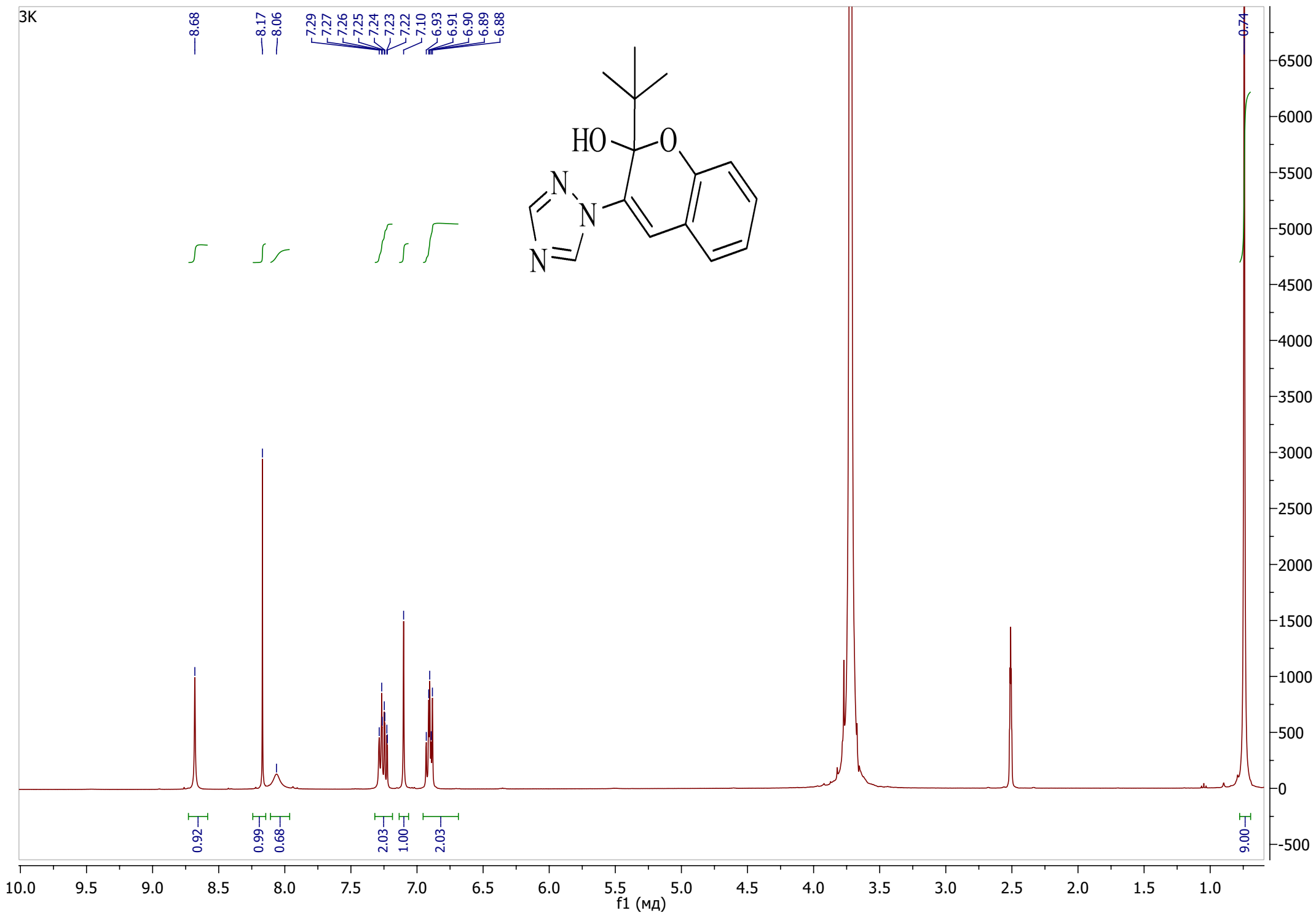

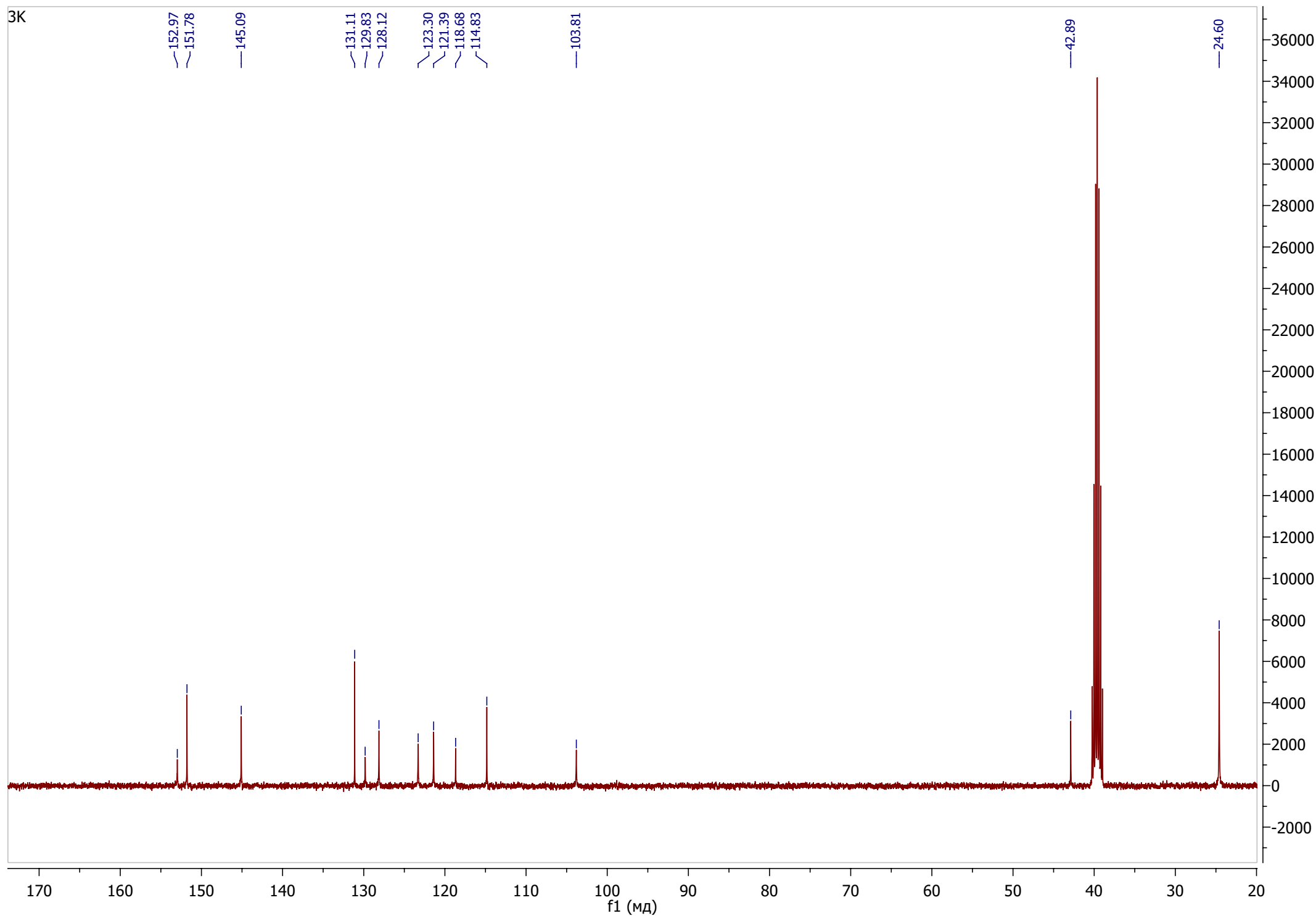

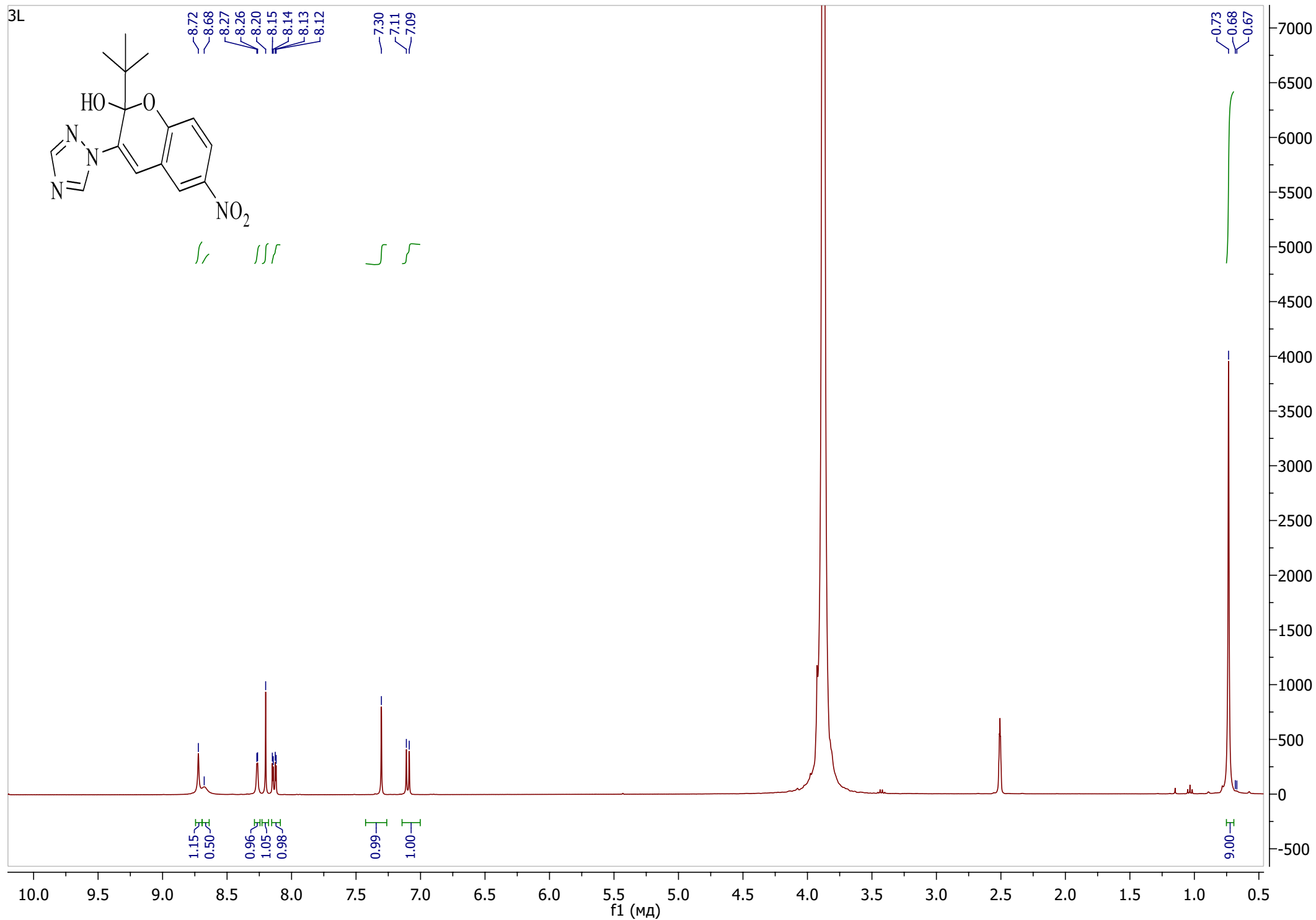

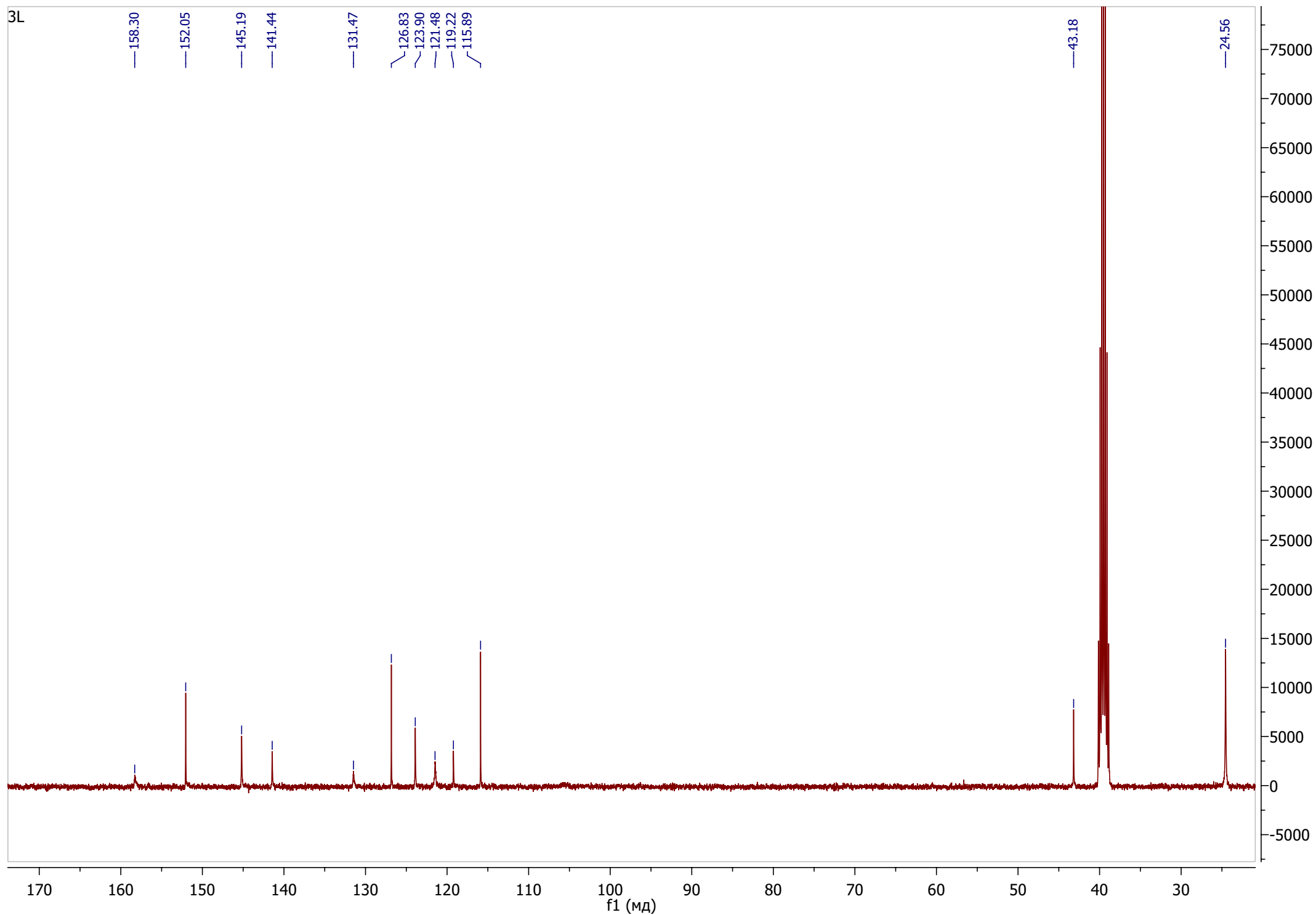

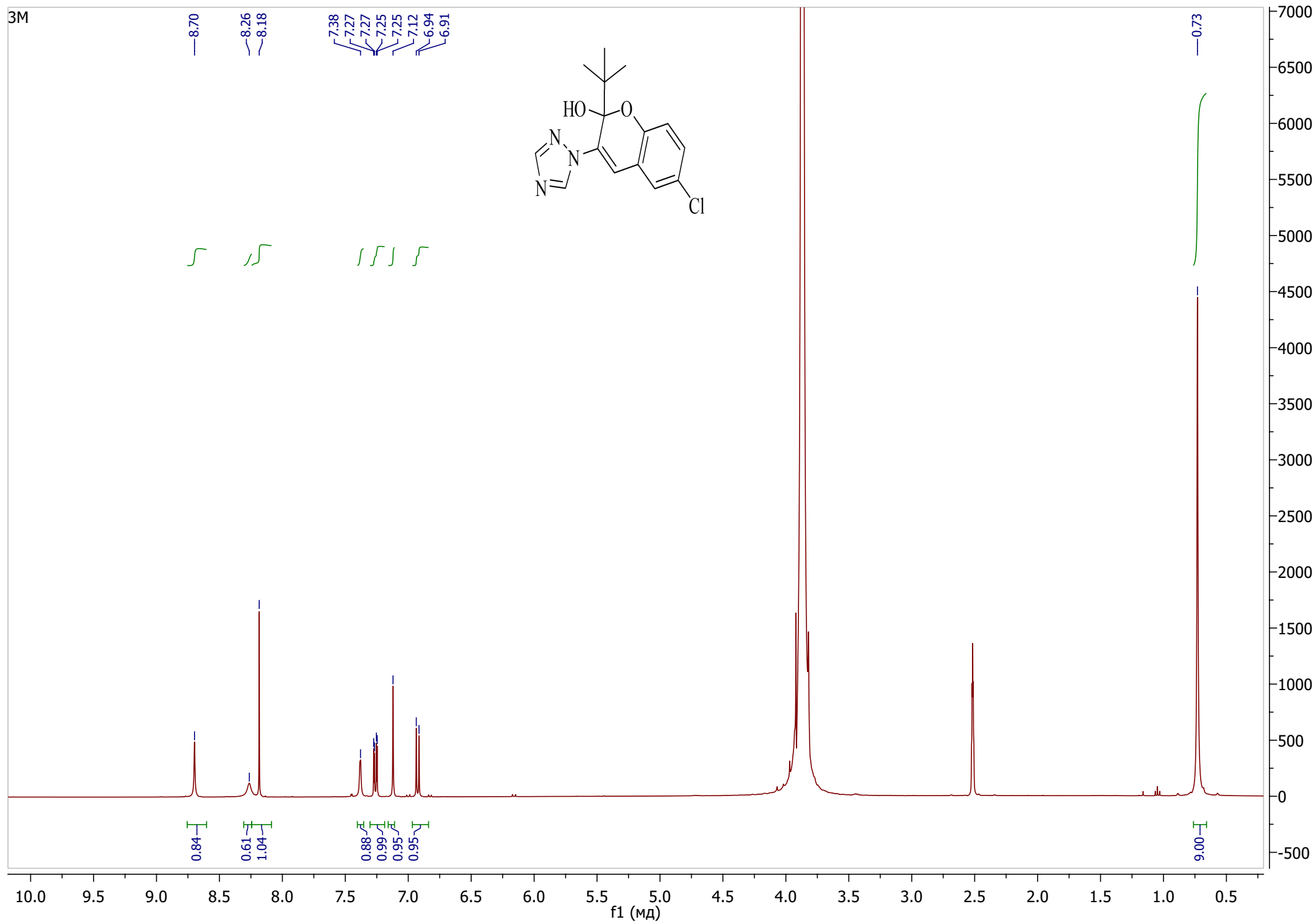

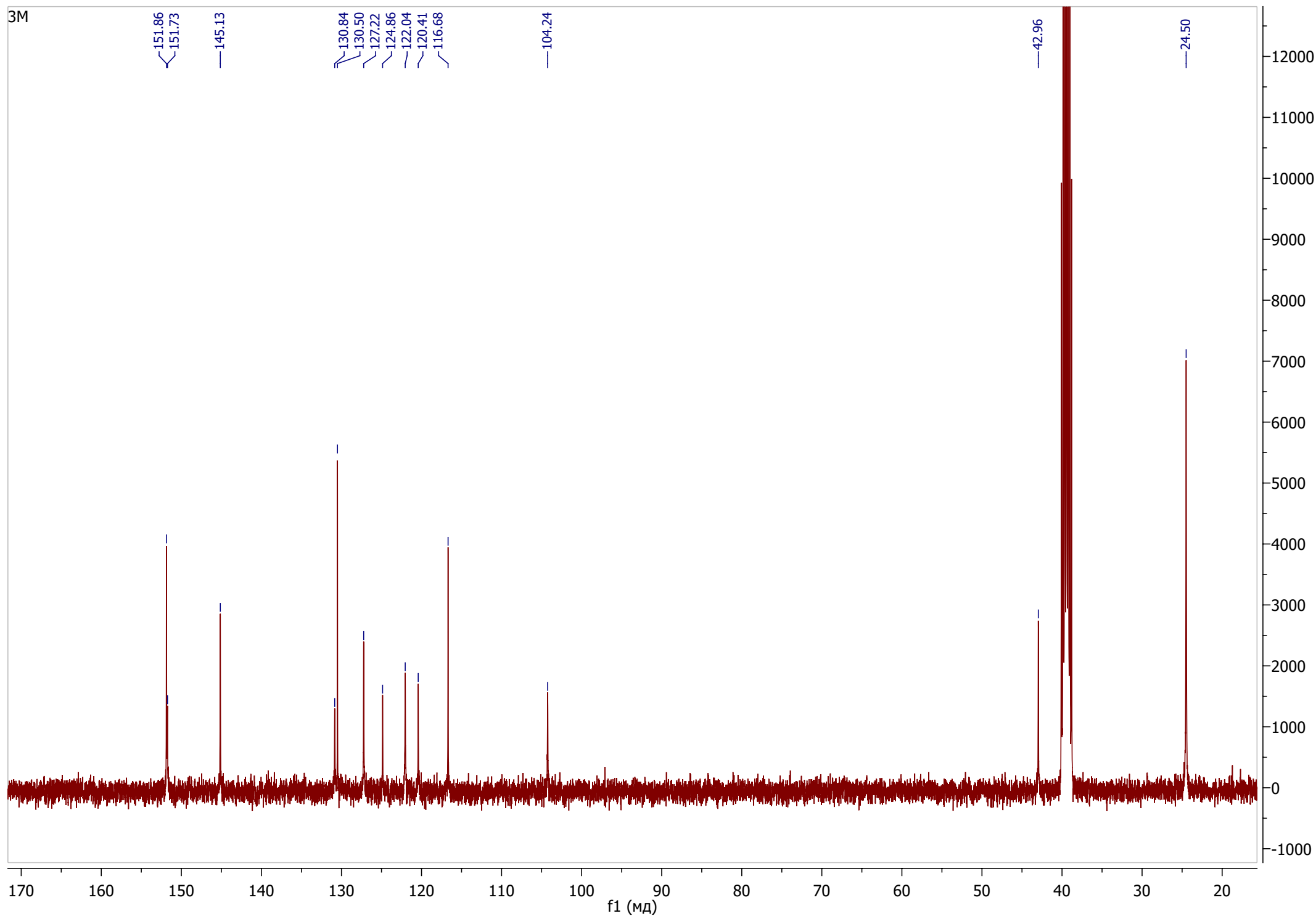

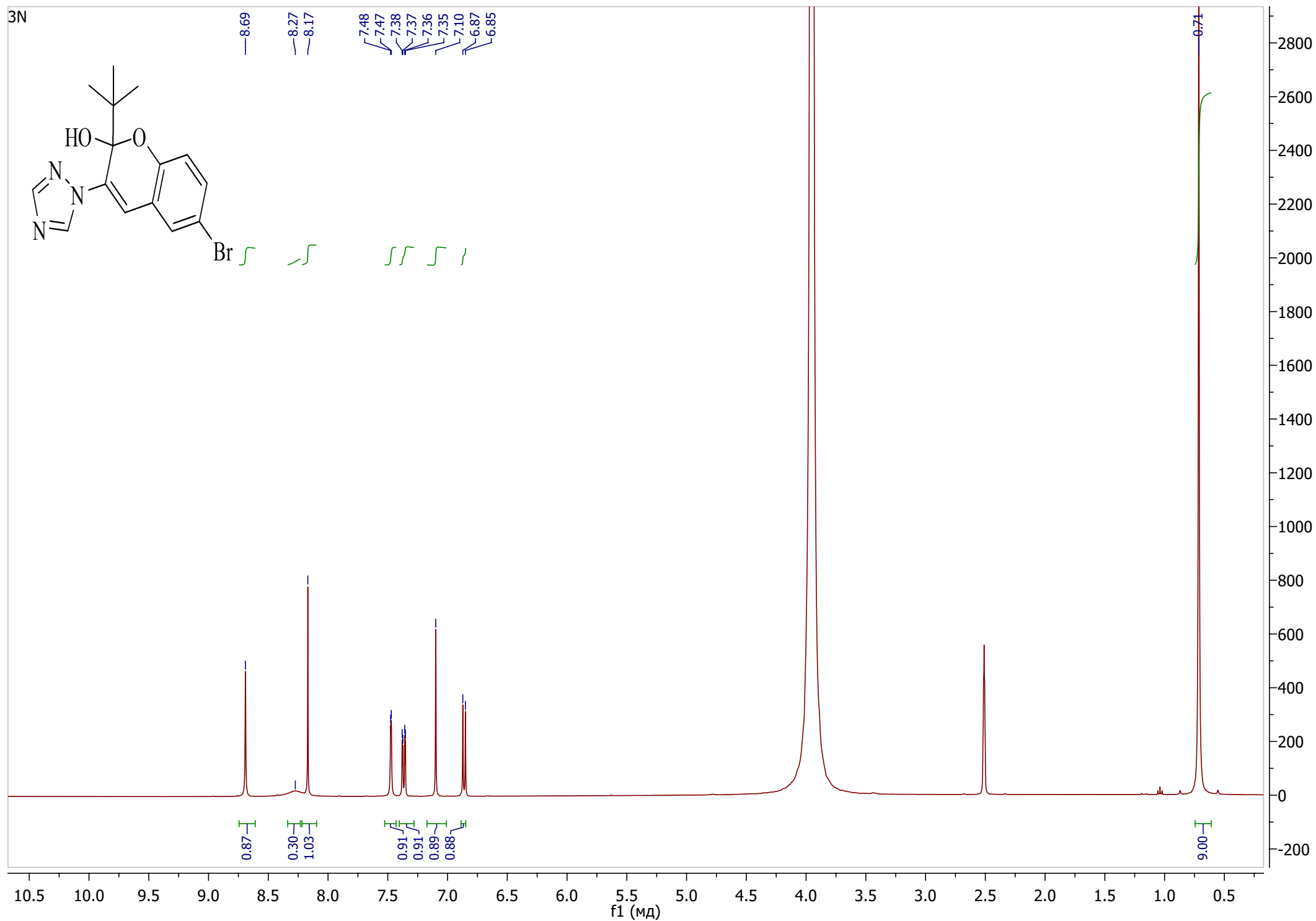

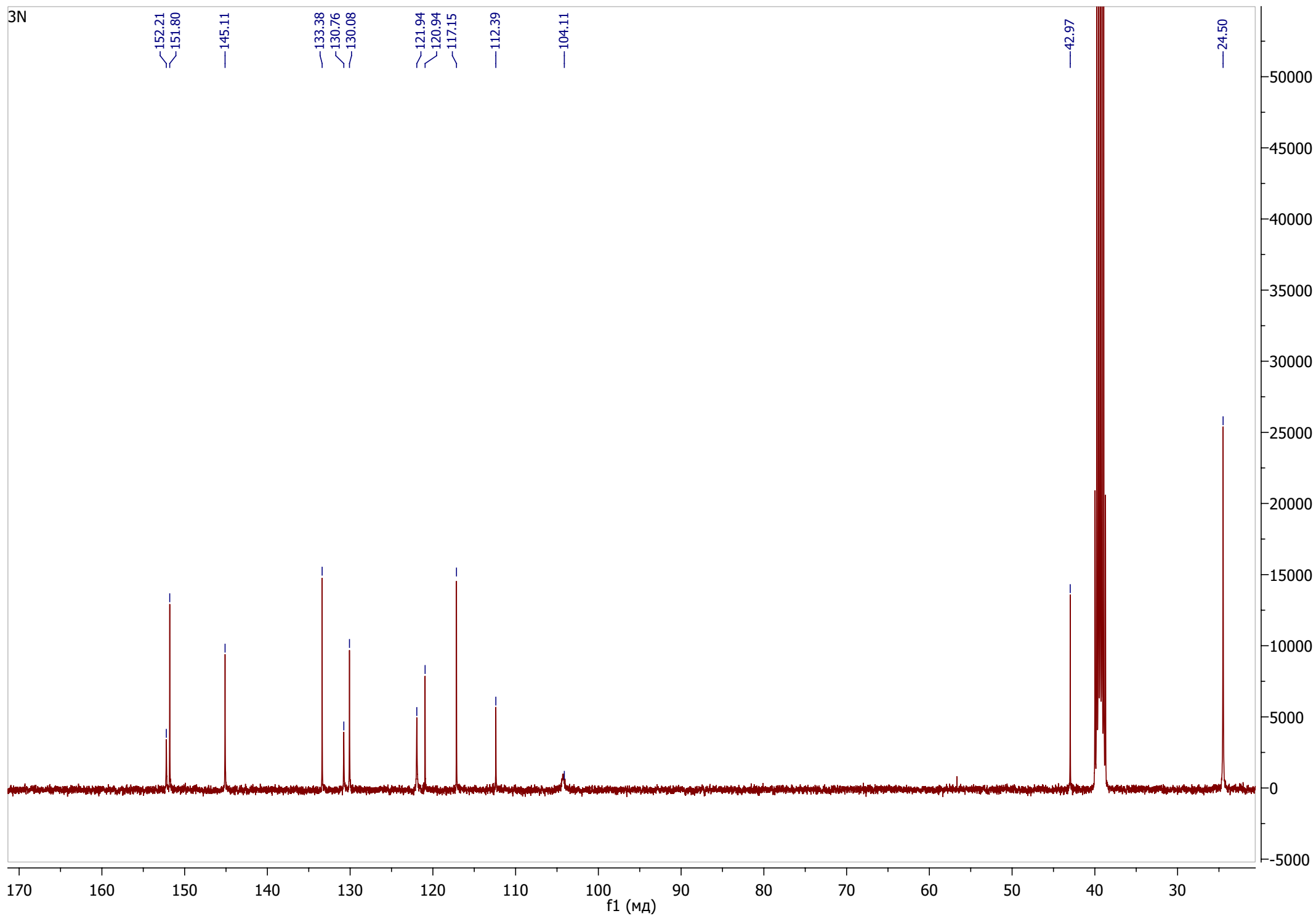

3a

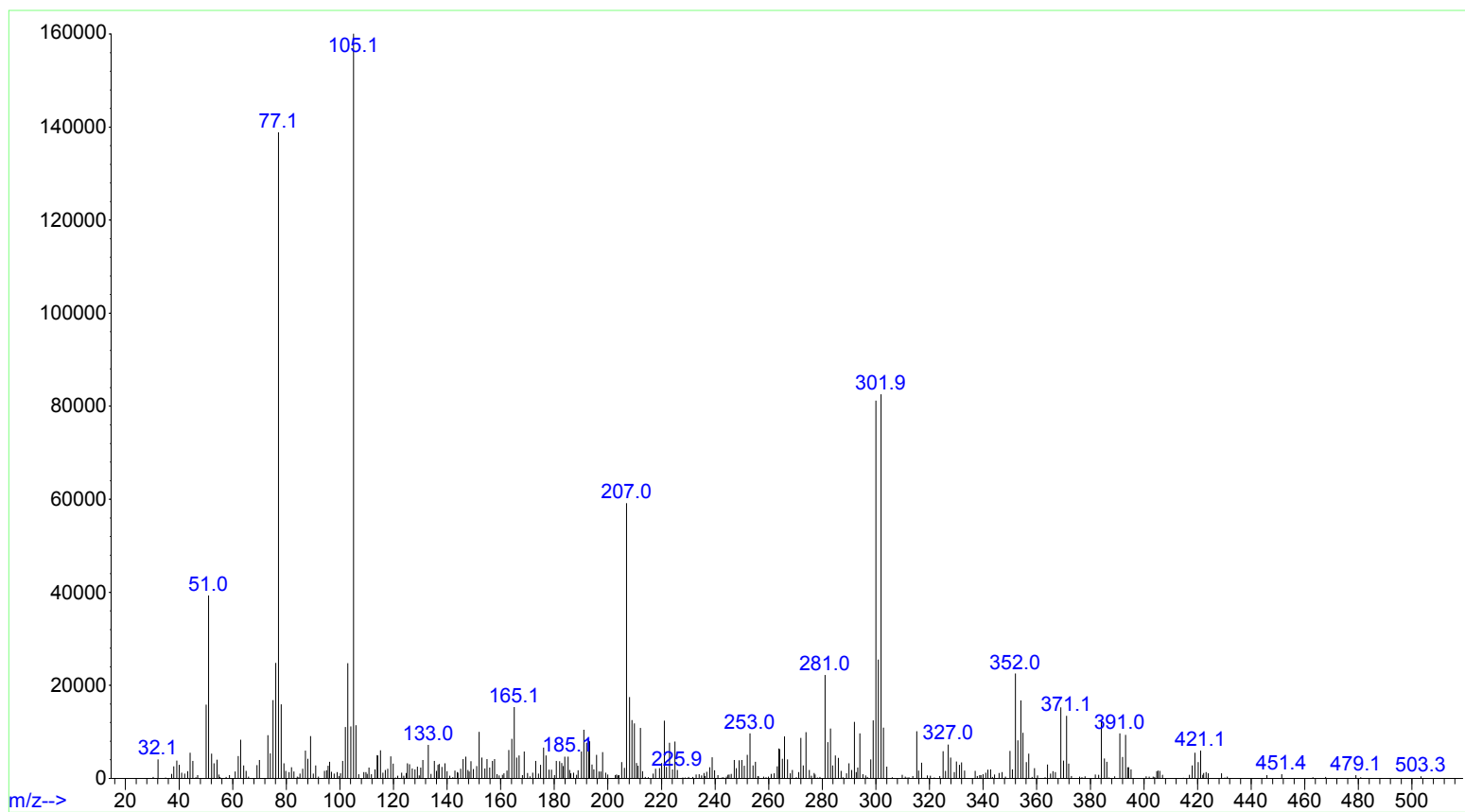

3b

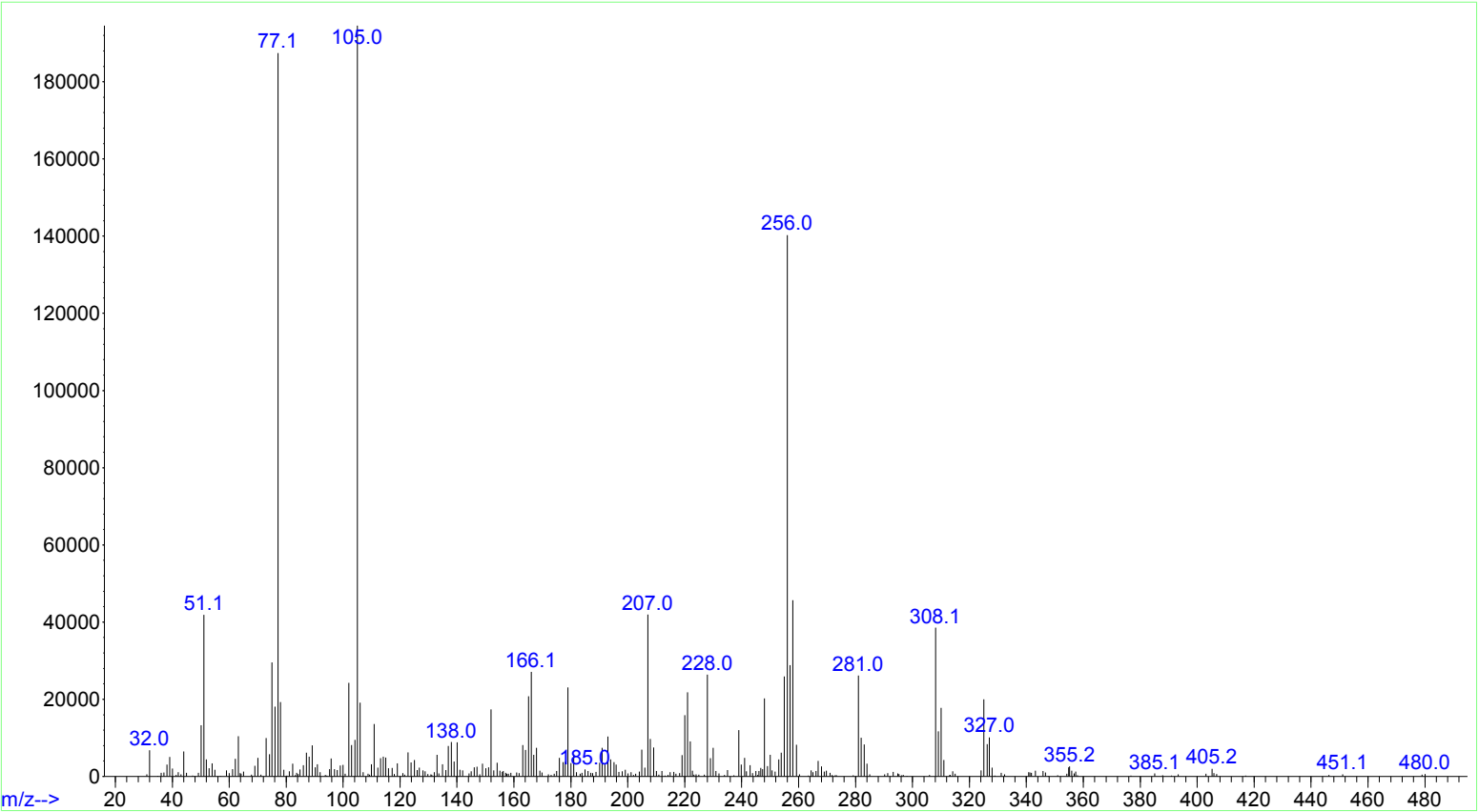

3c

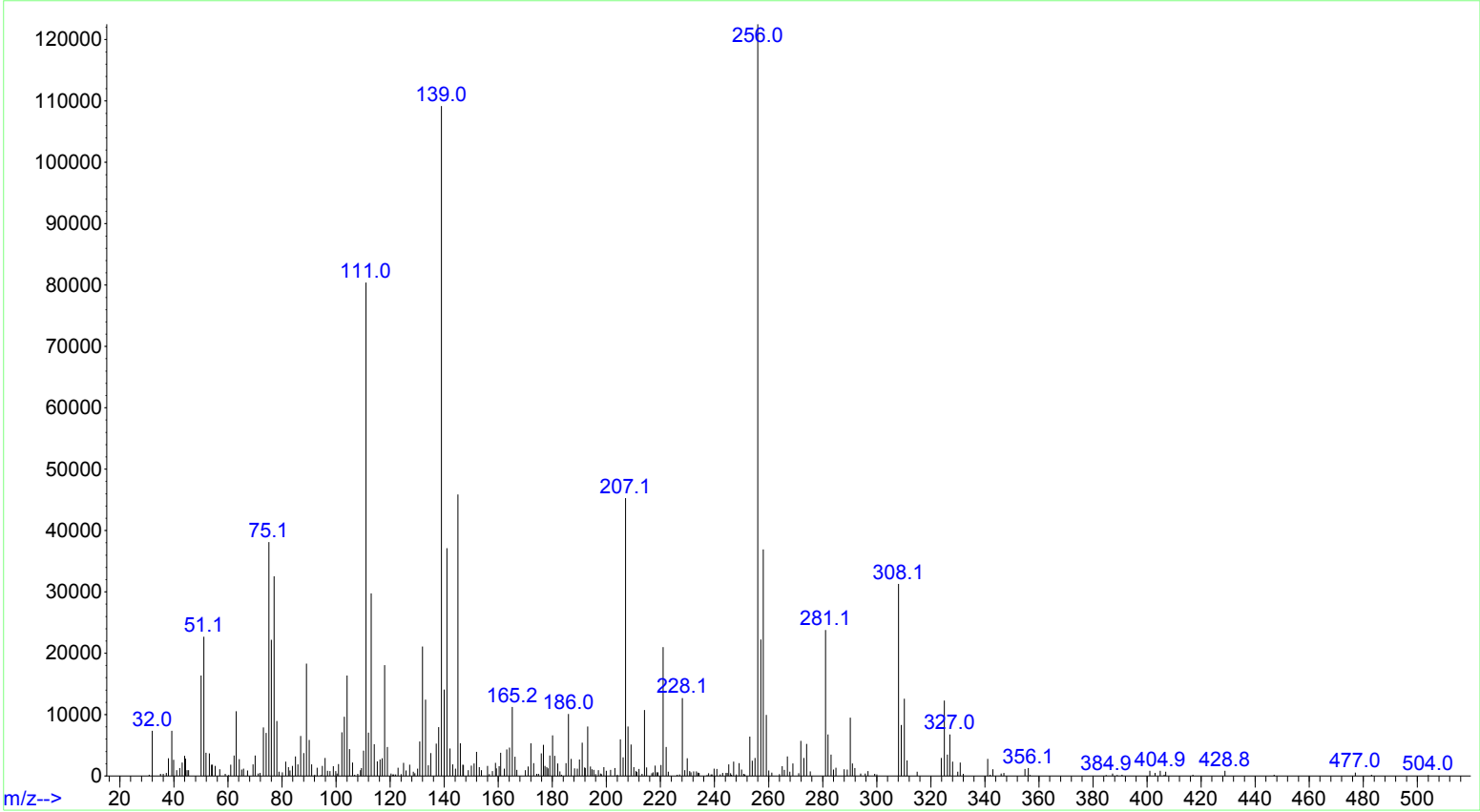

3d

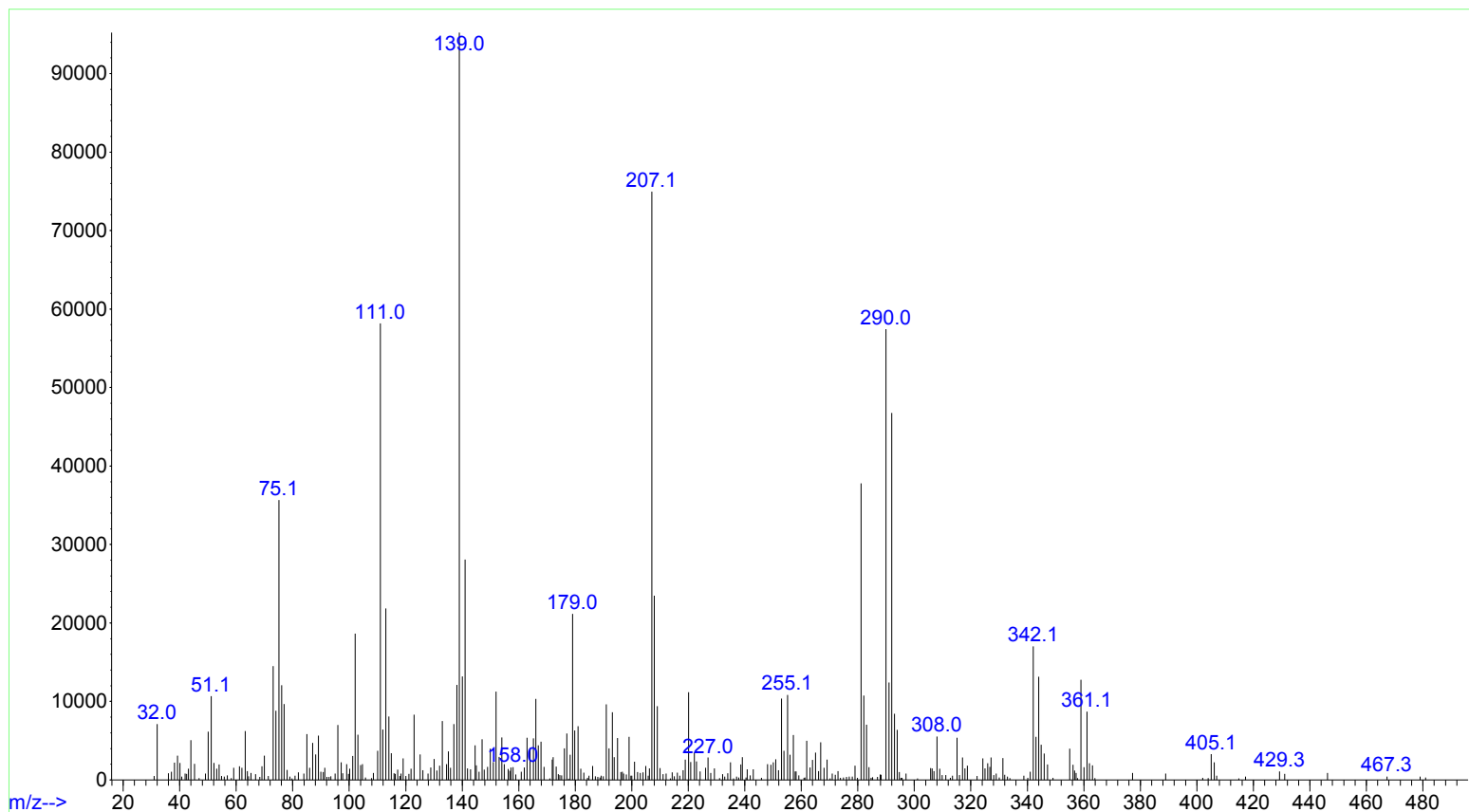

3e

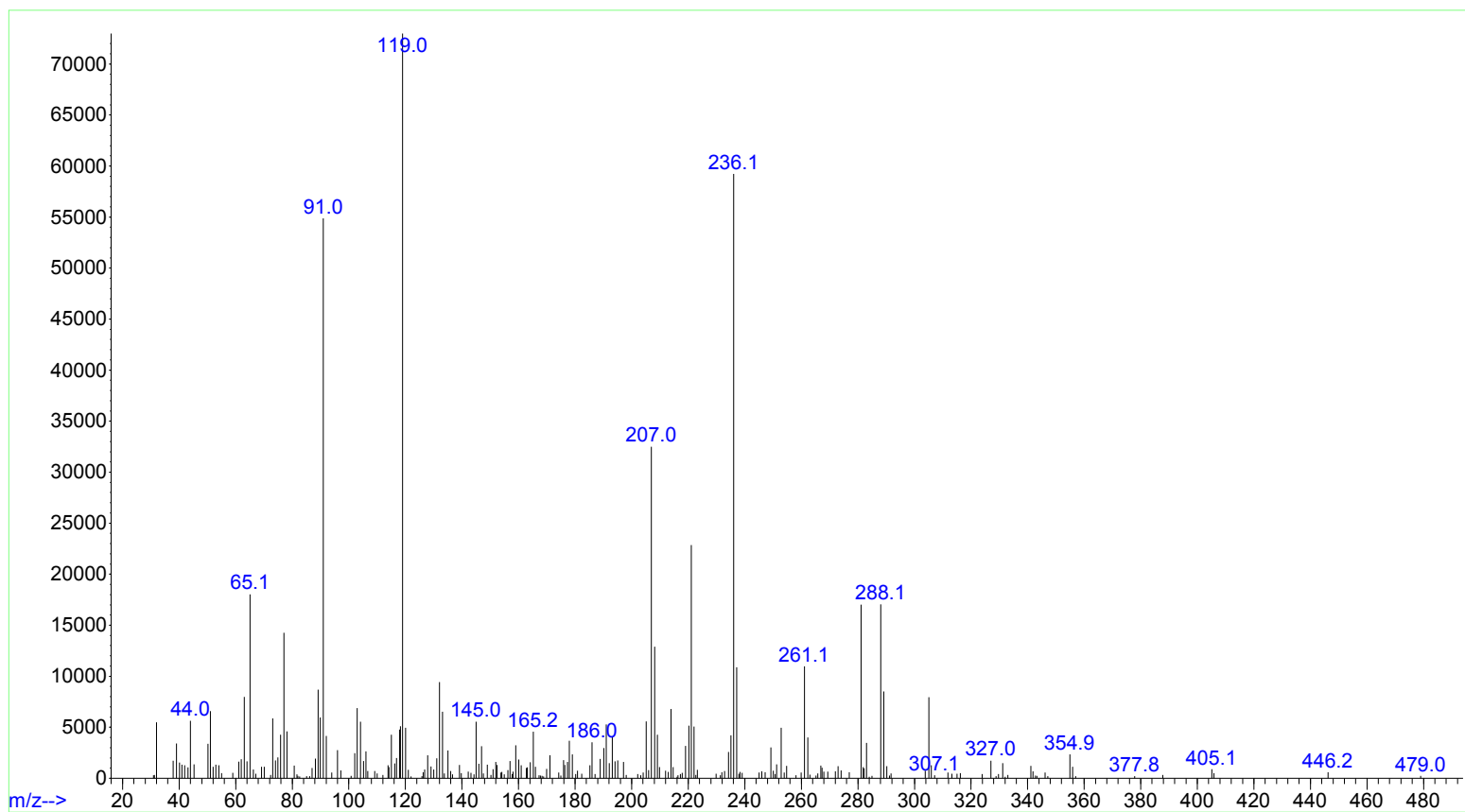

3j

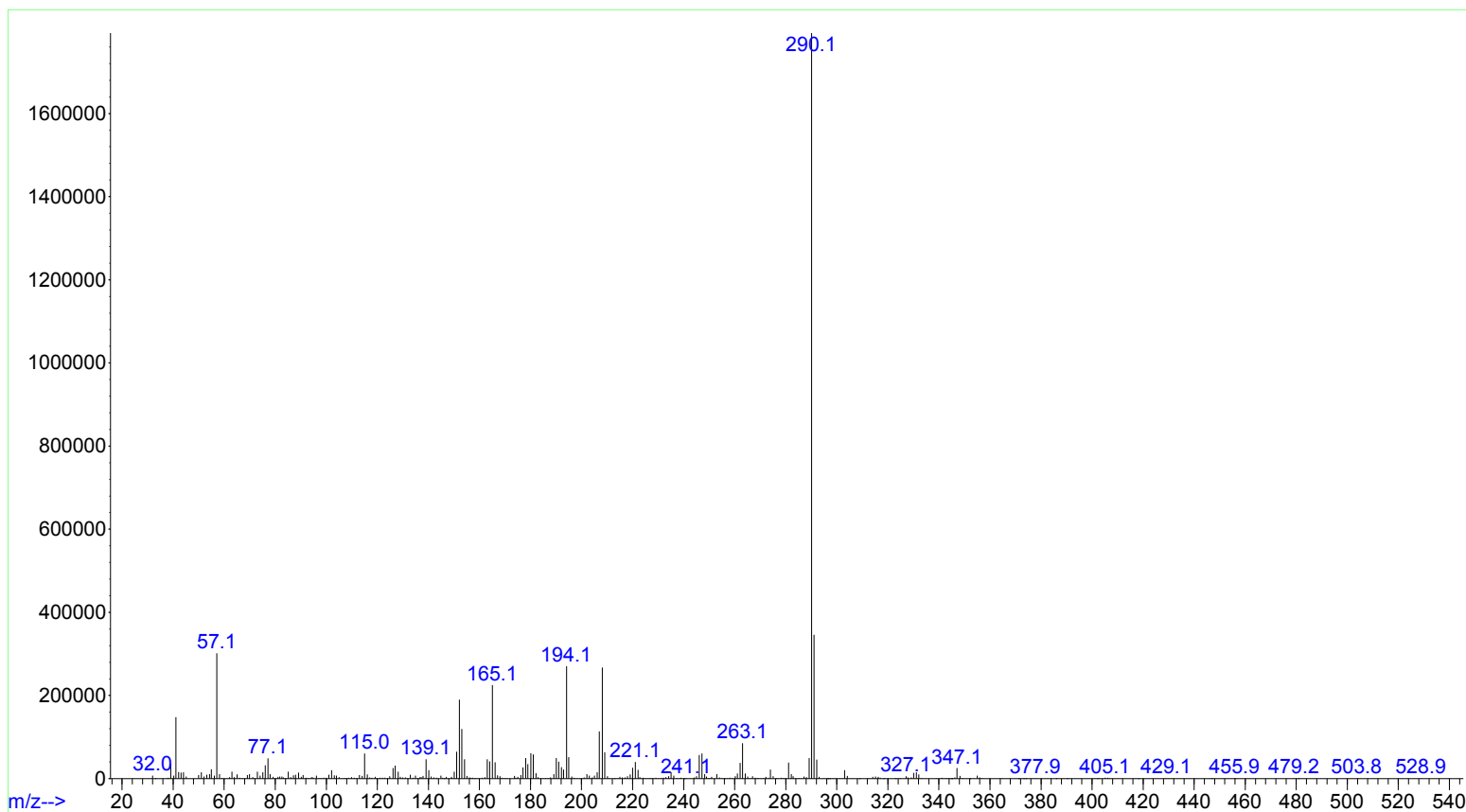

3k

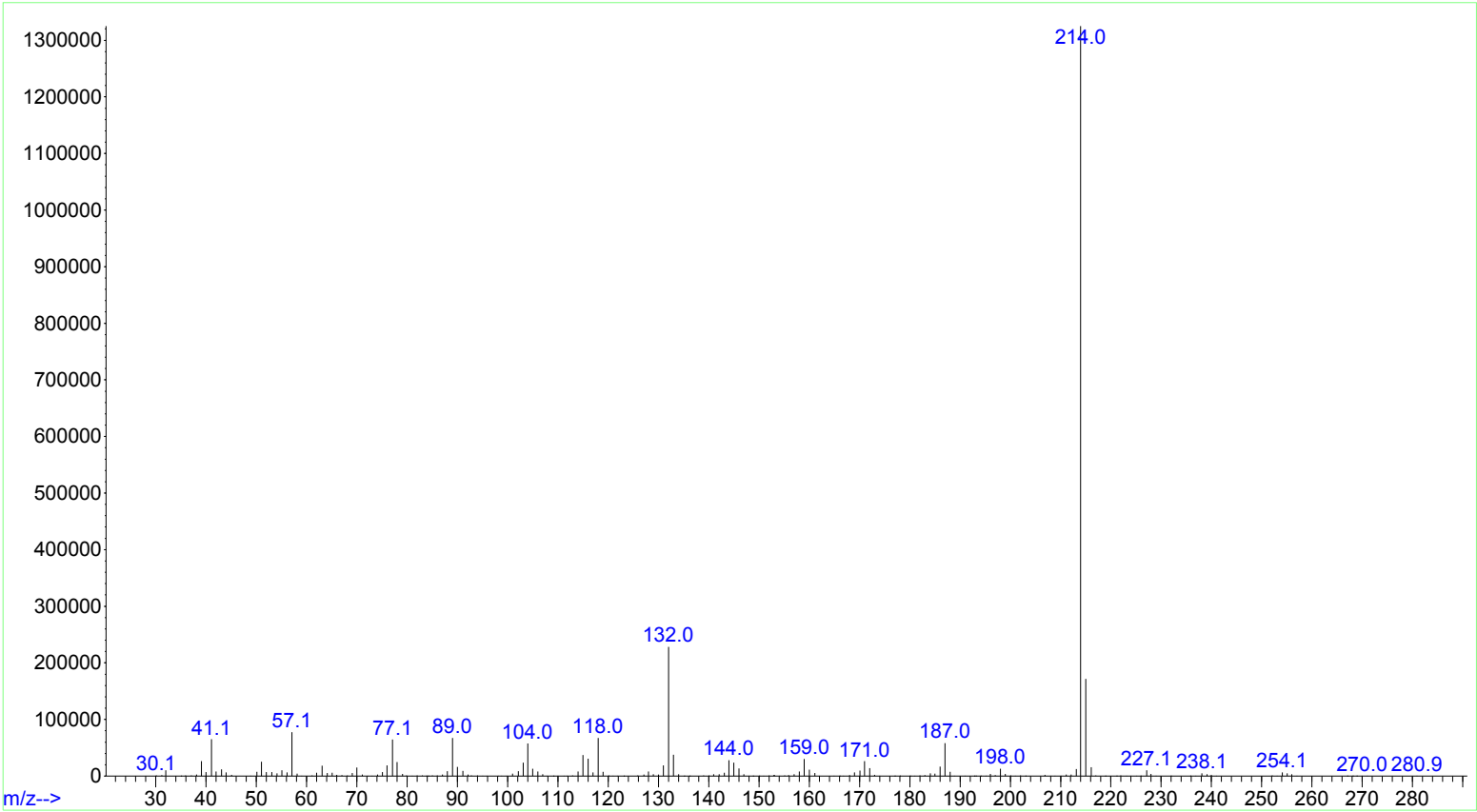

3L

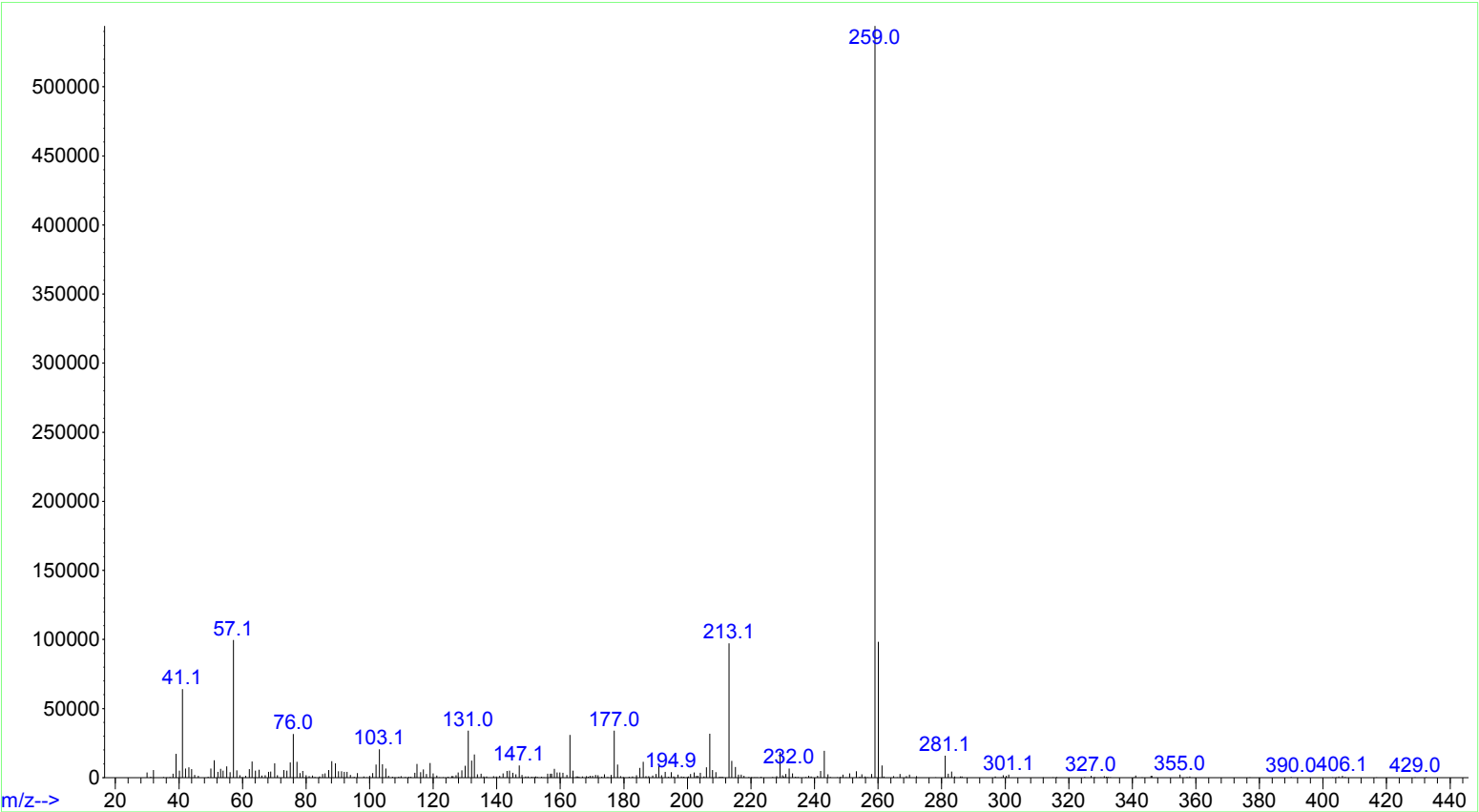

3m

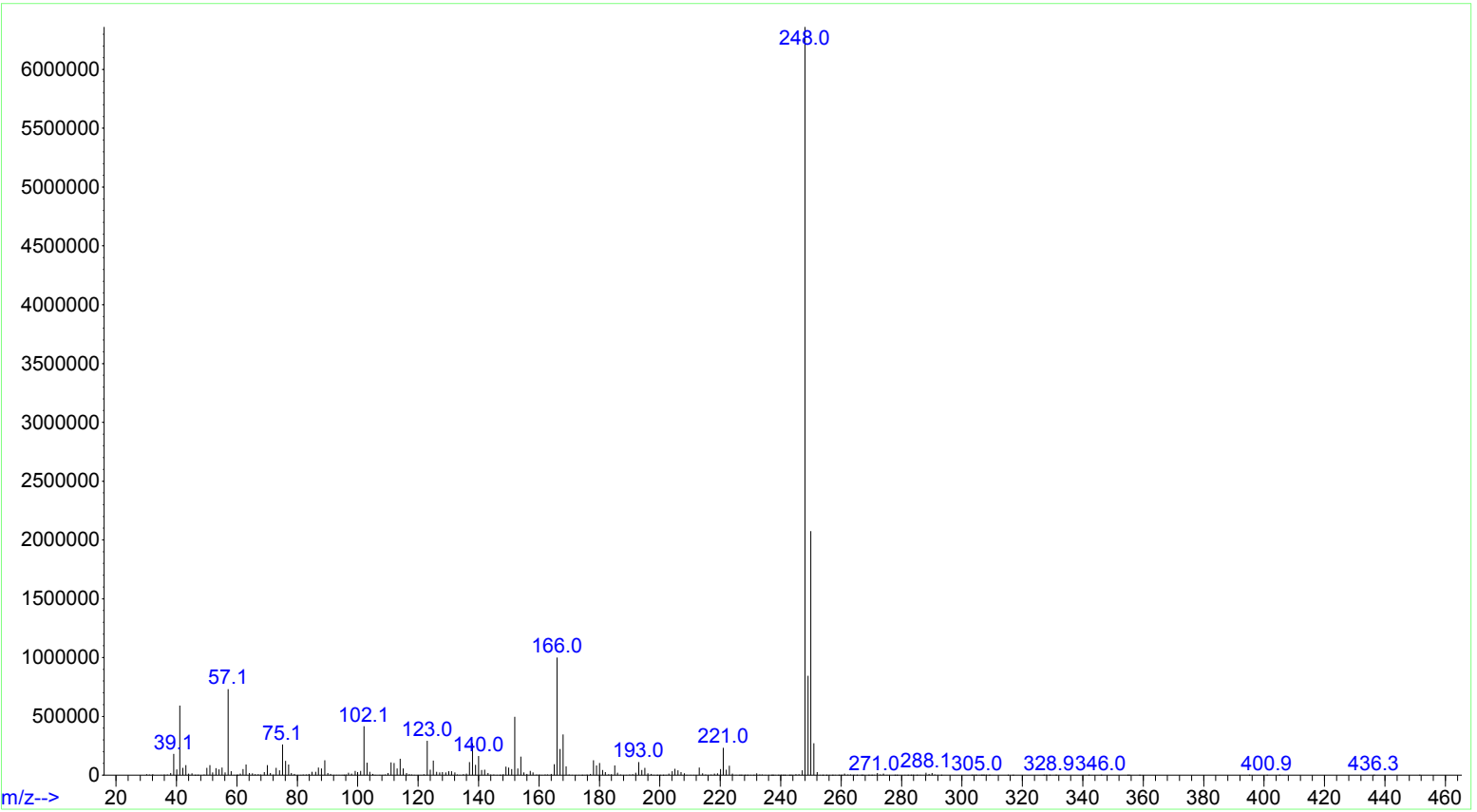

3N

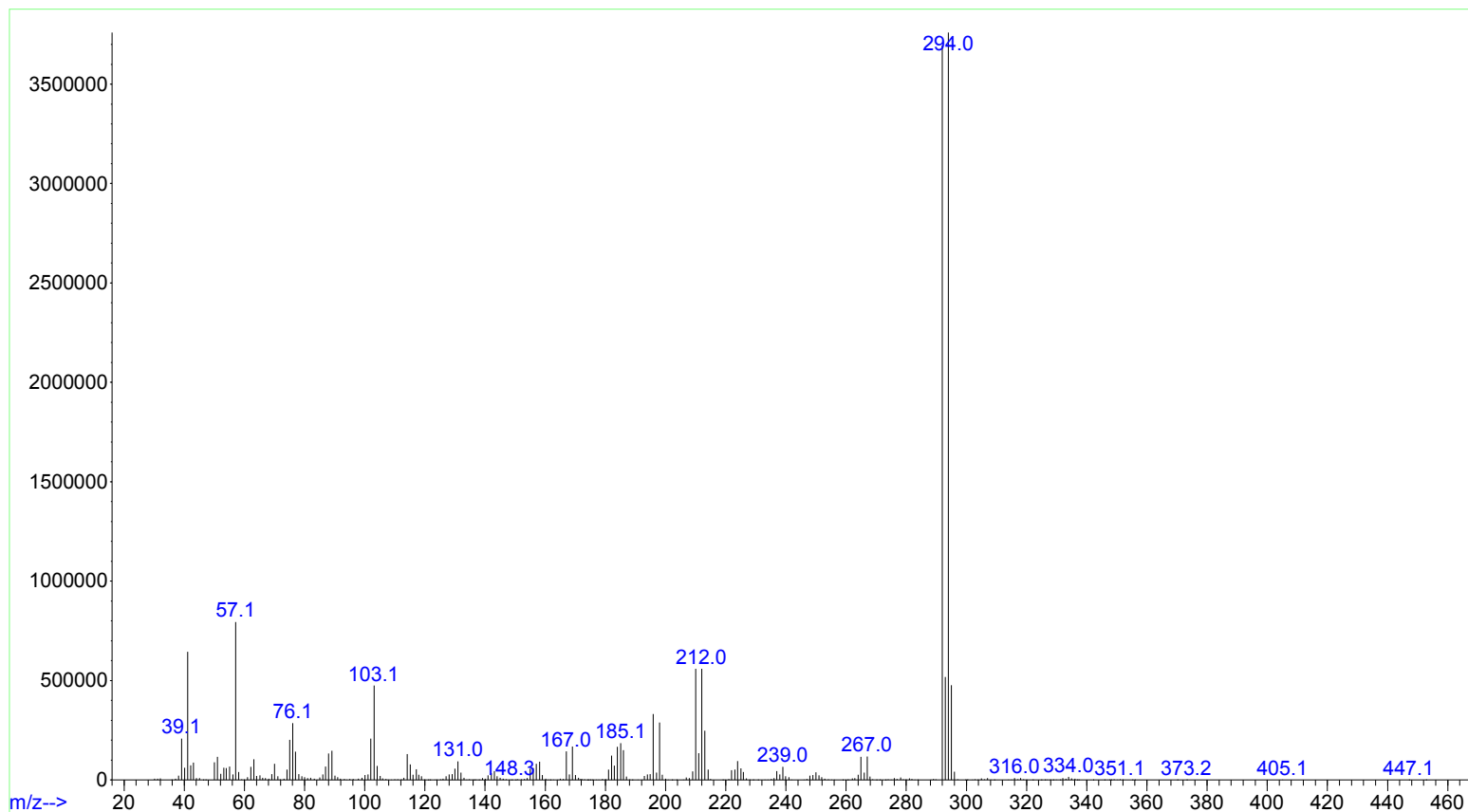

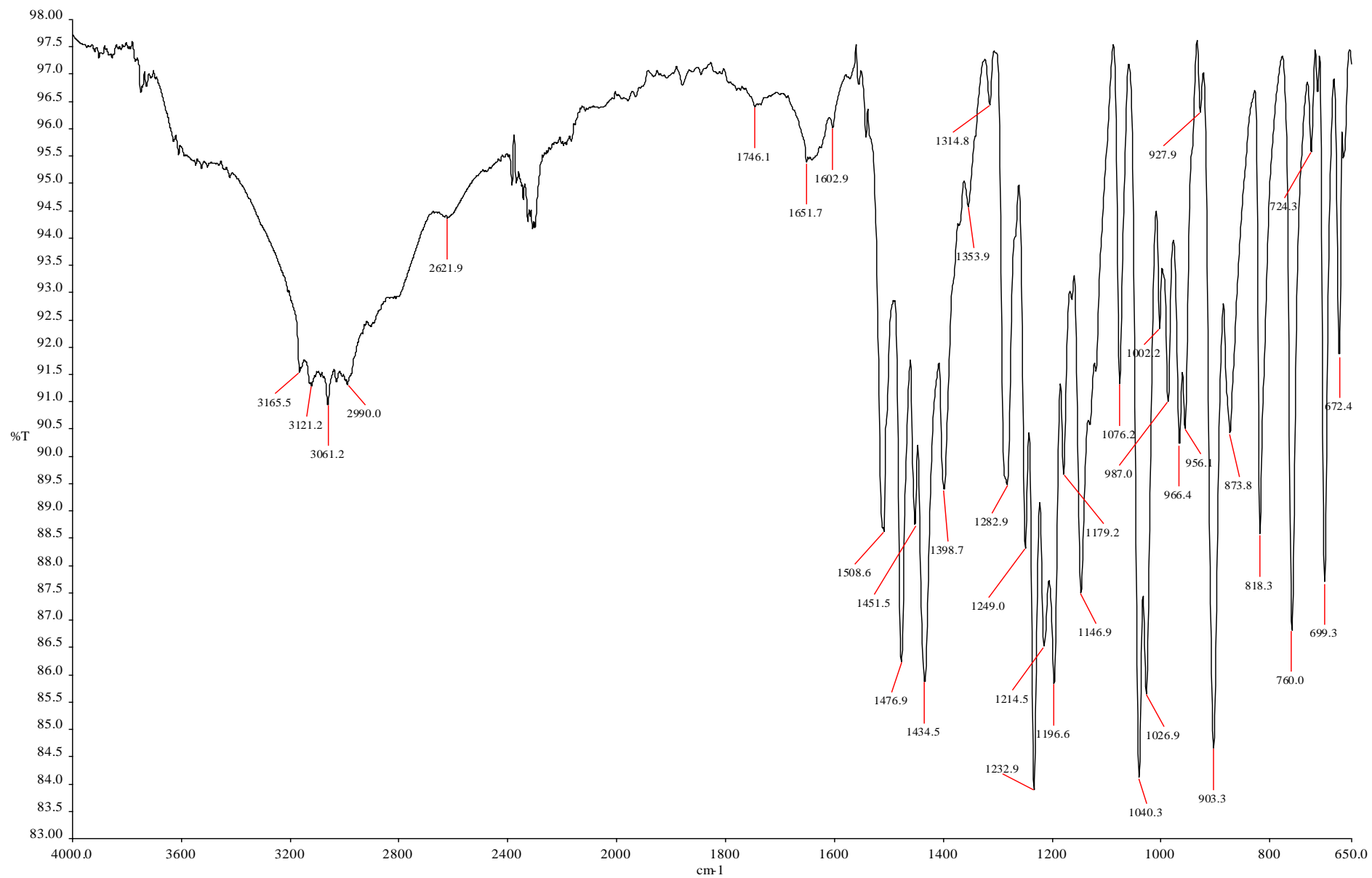

3a

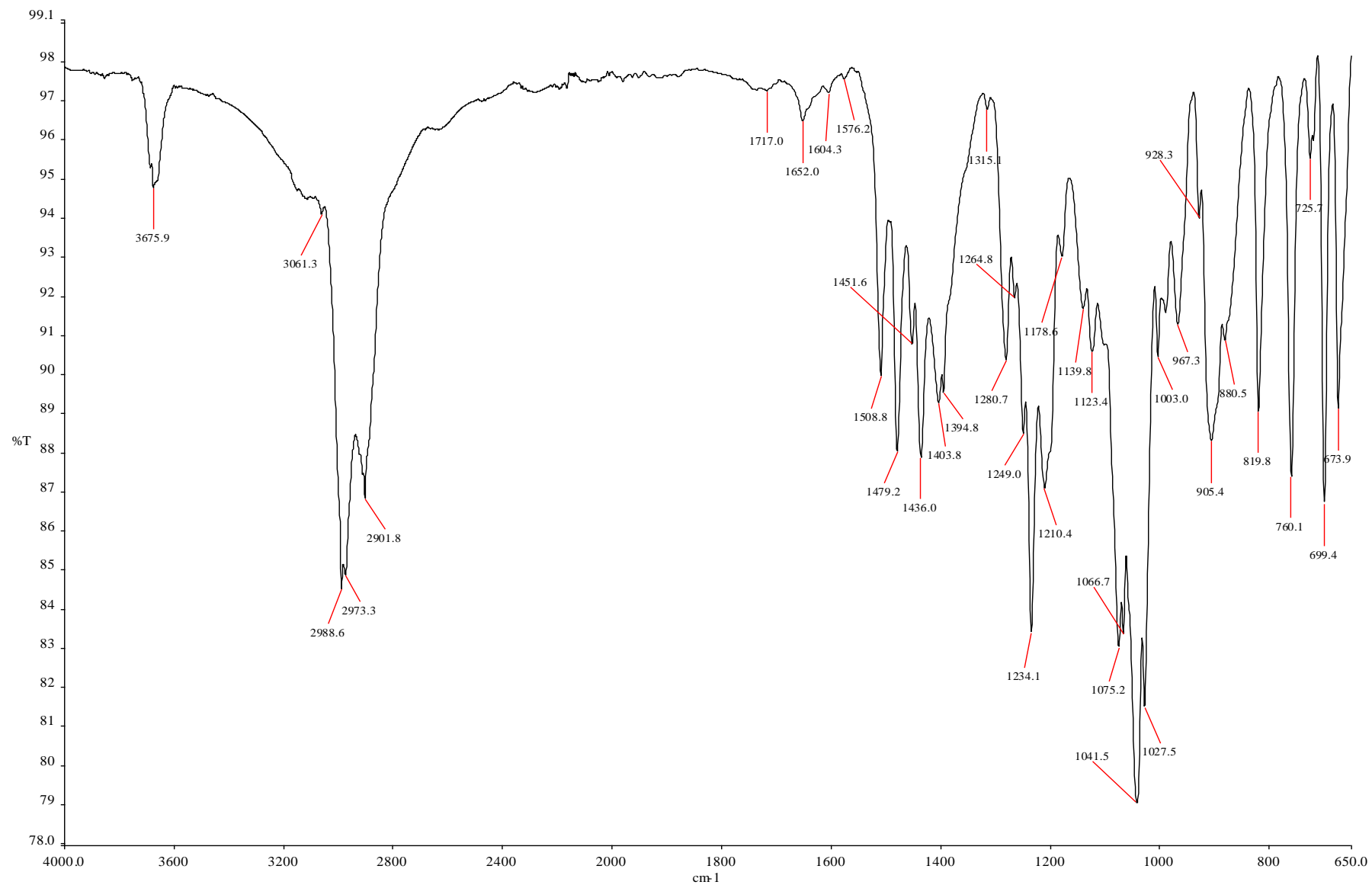

**3b**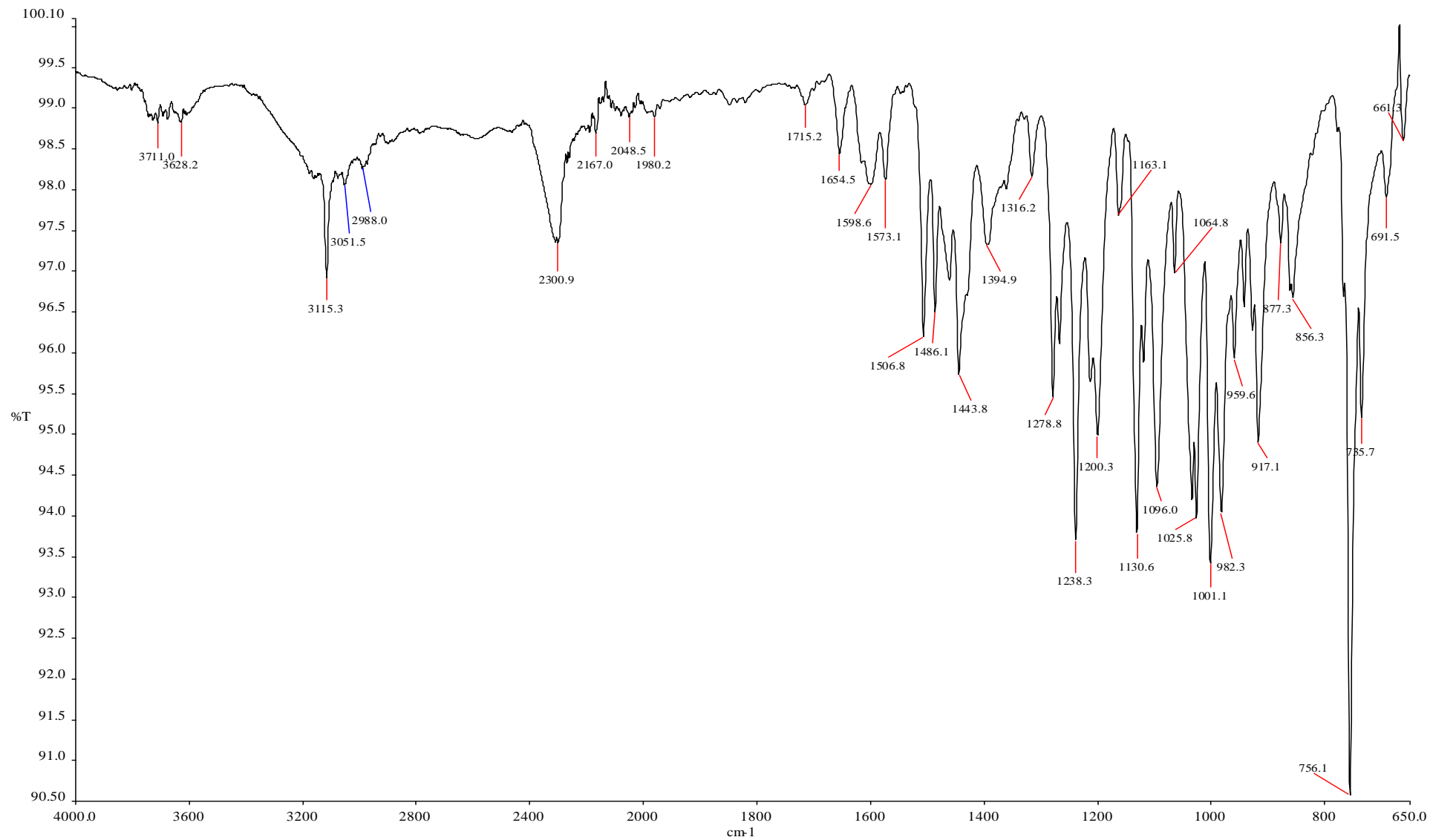**3c**

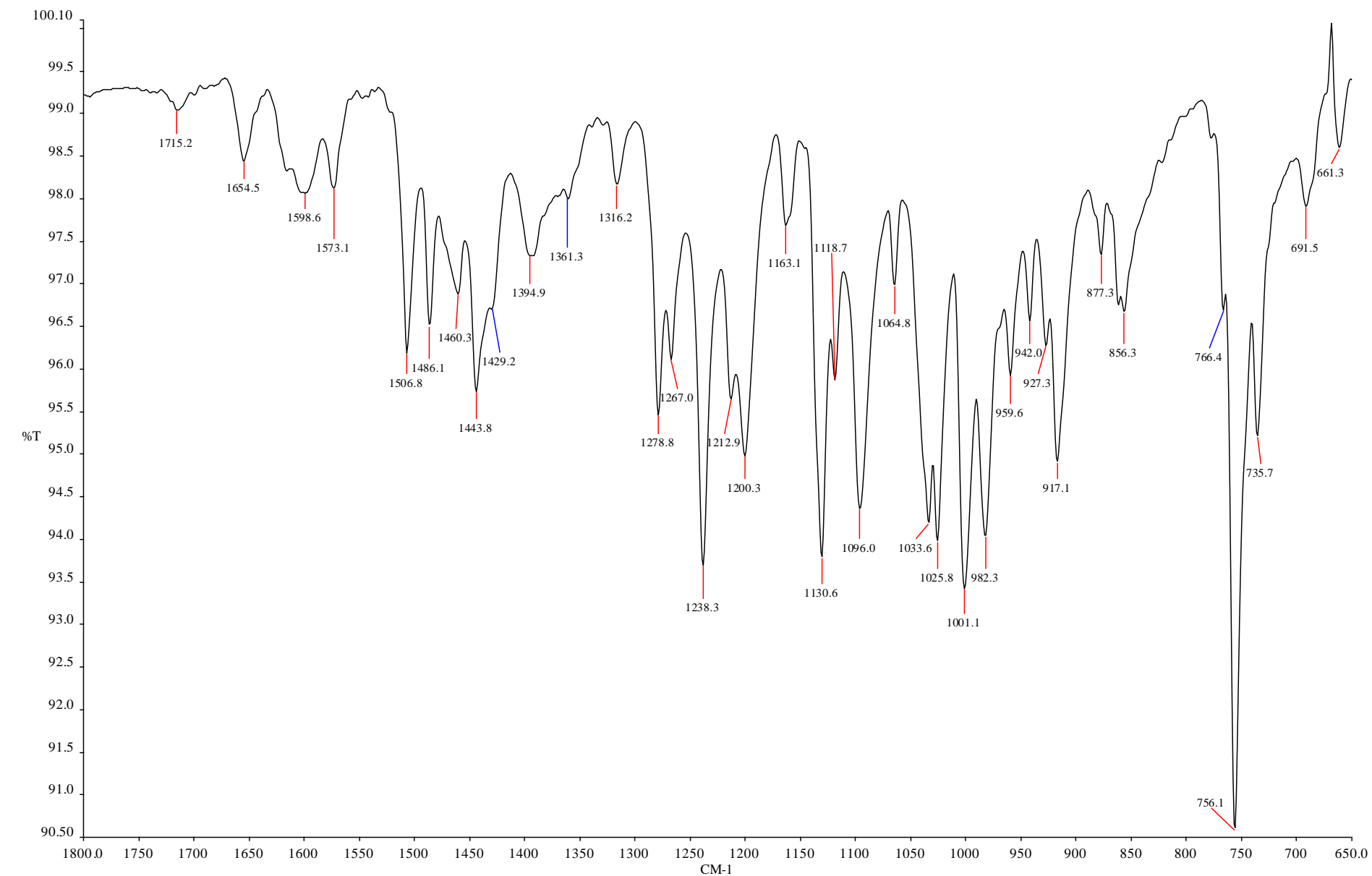

3c

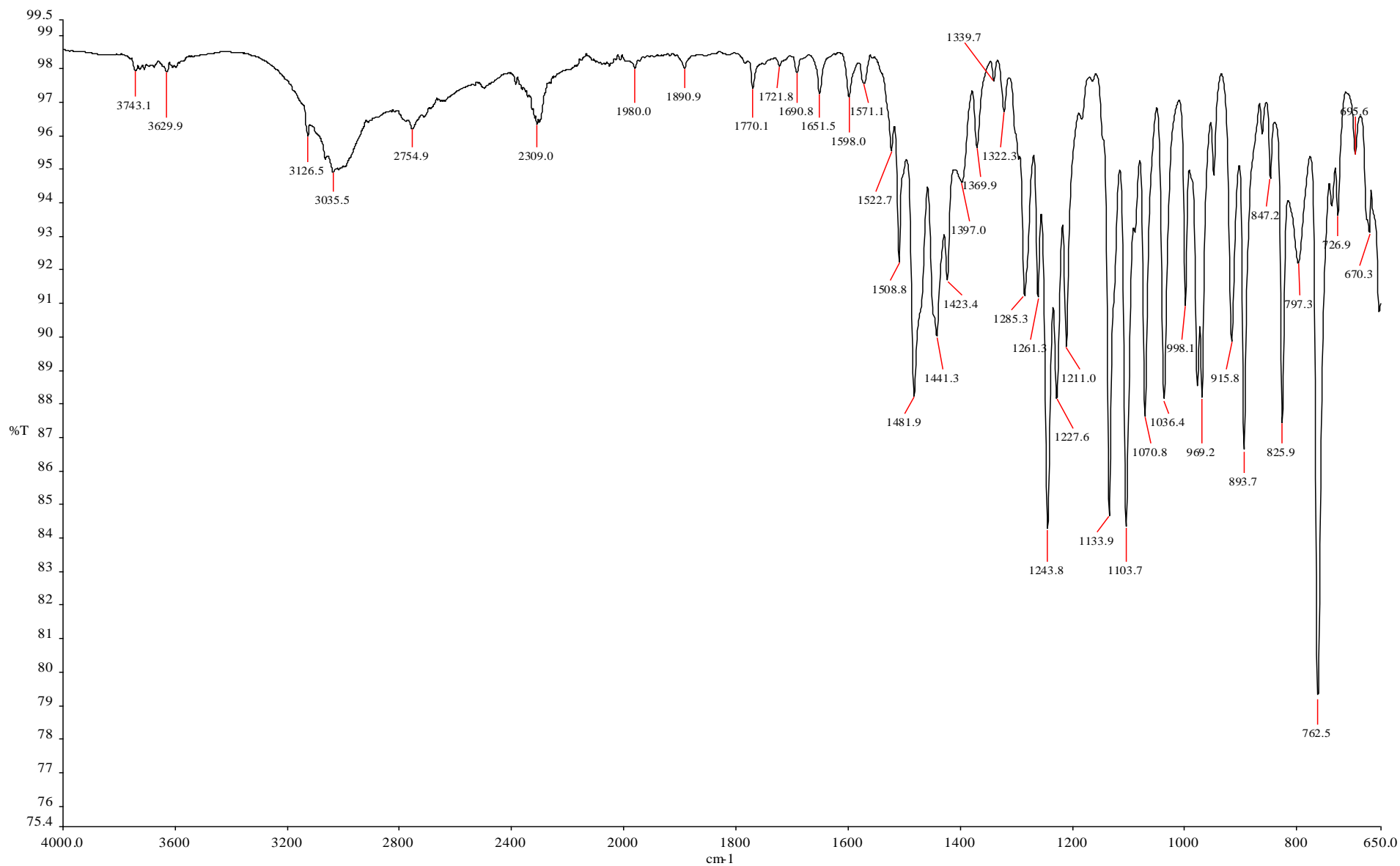

3d

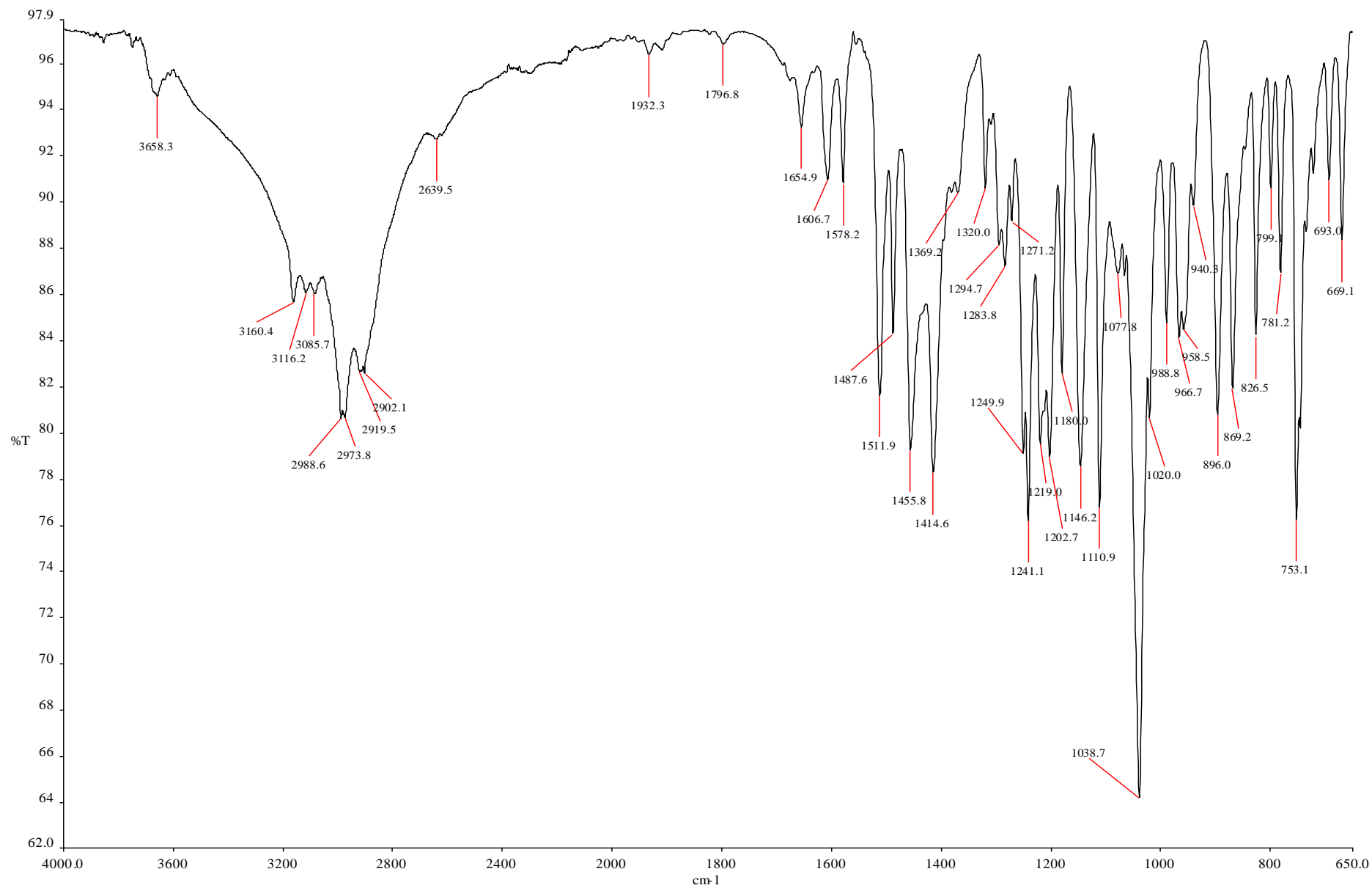

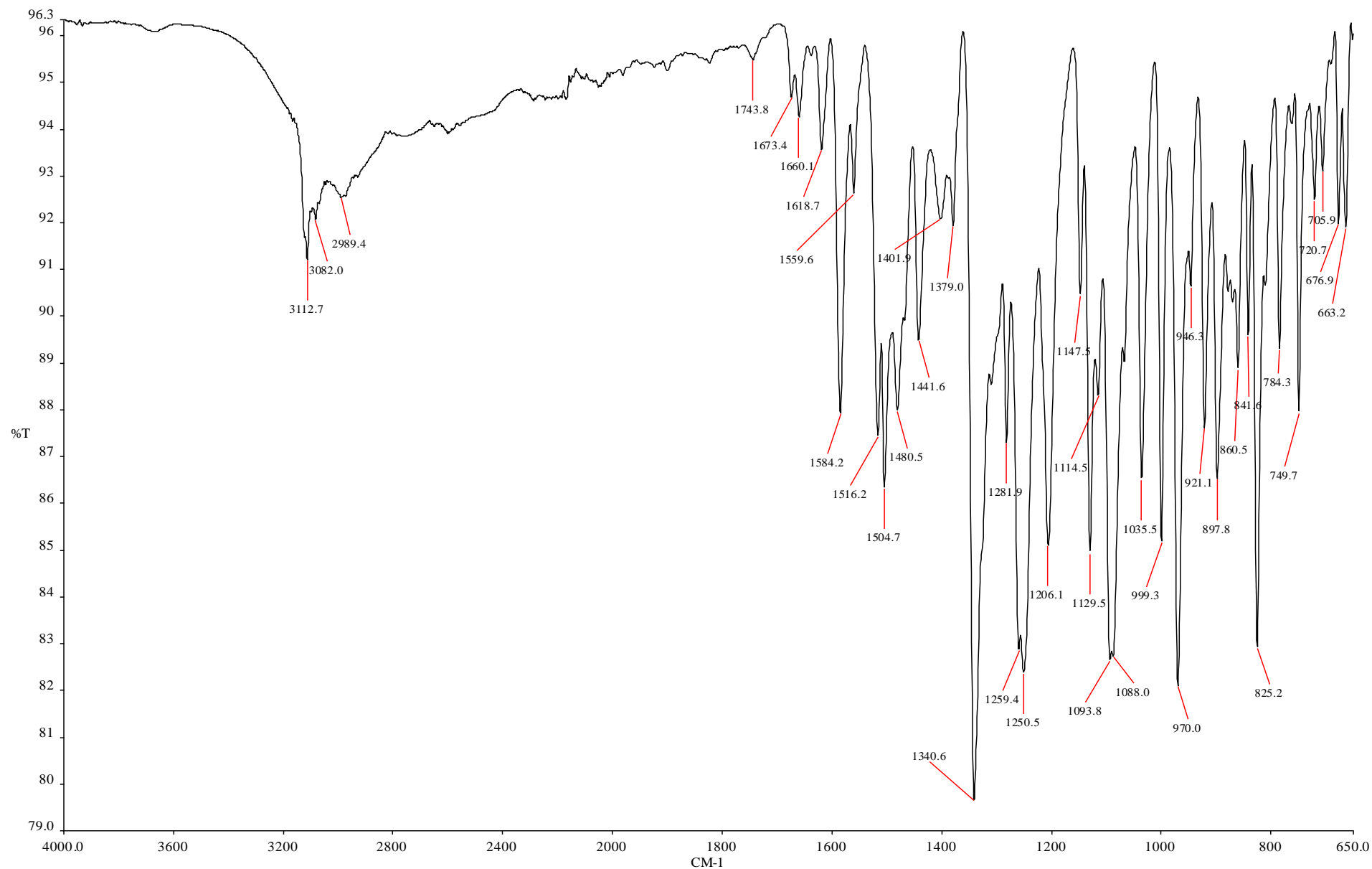

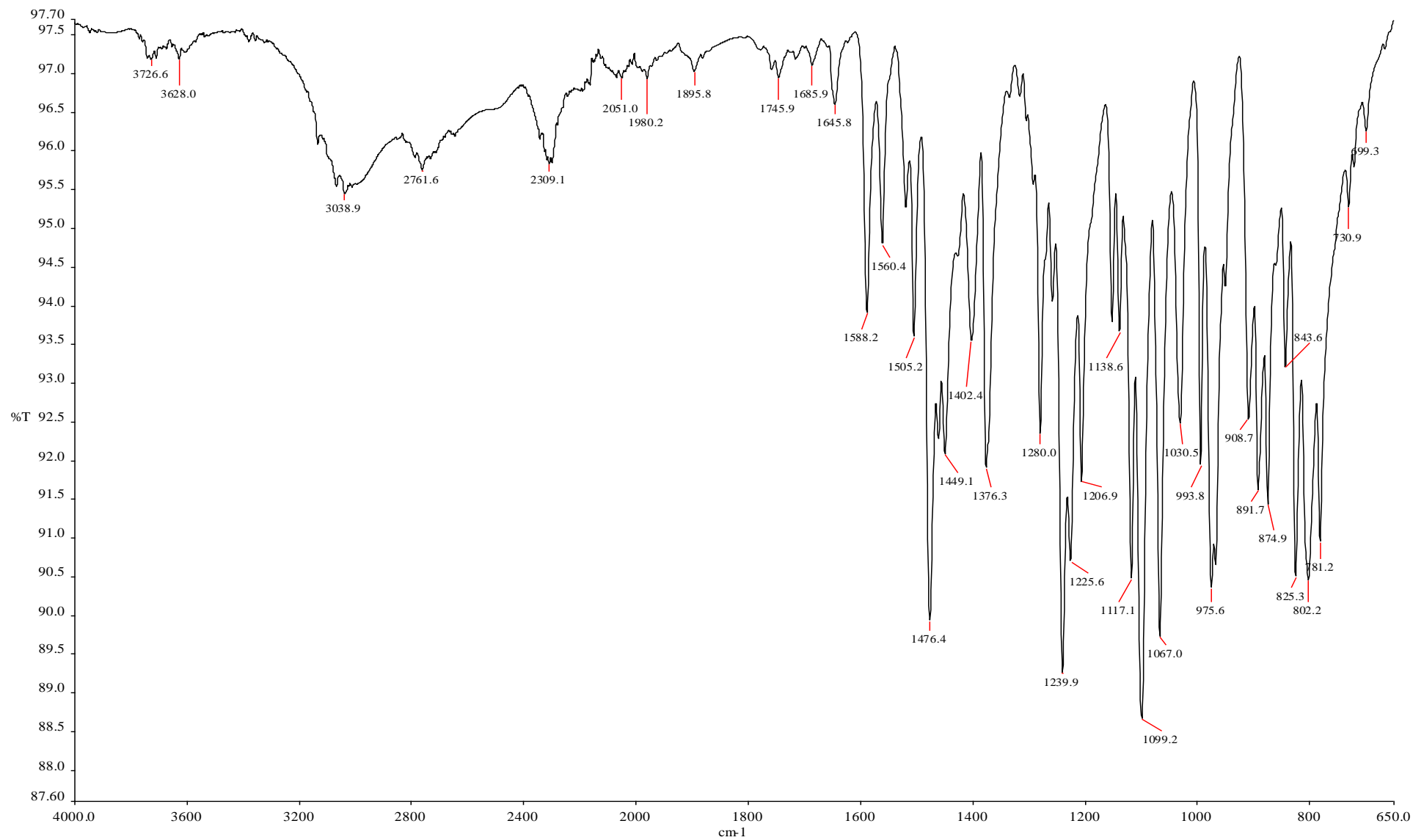

3g

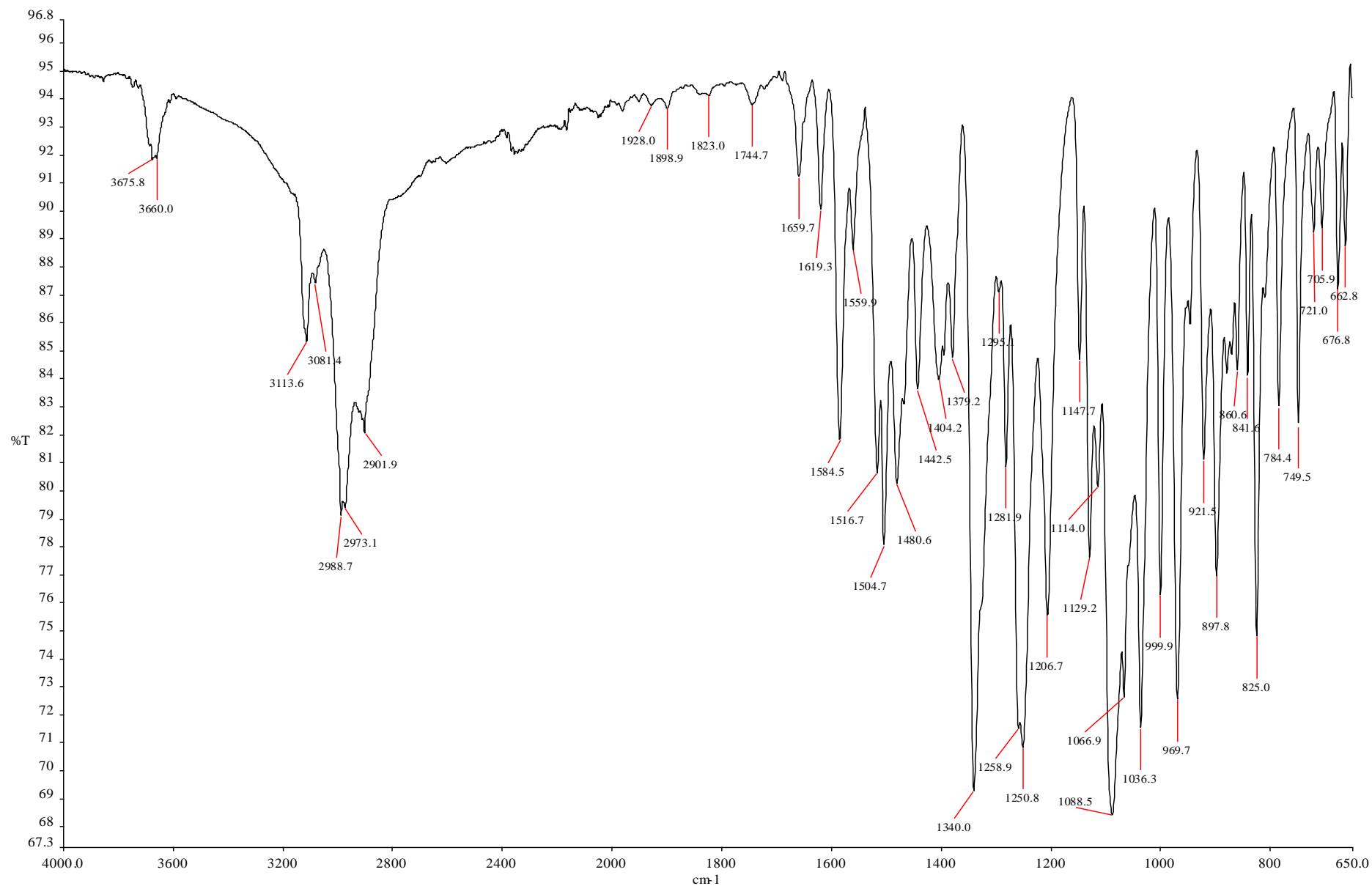

3h

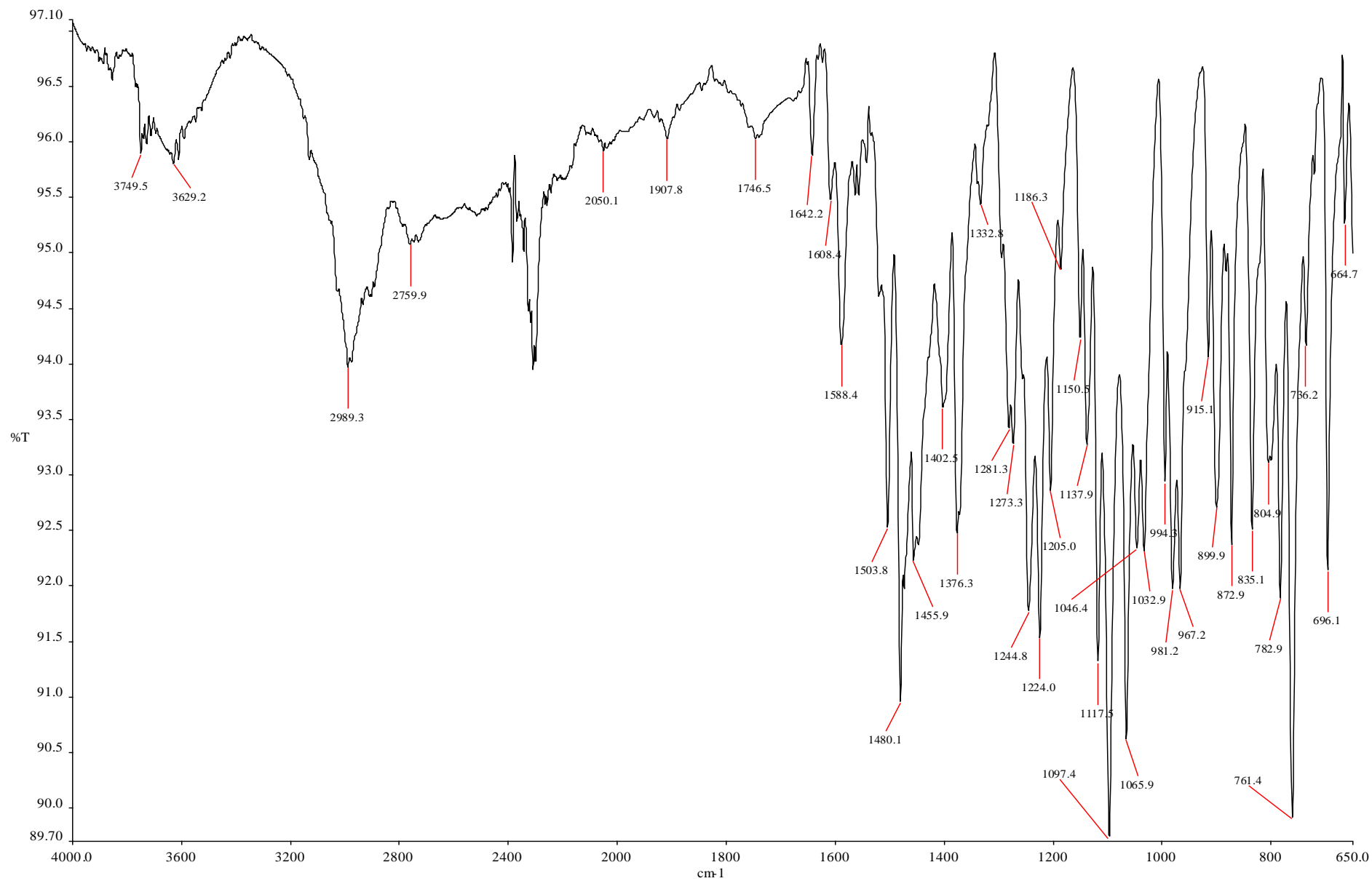

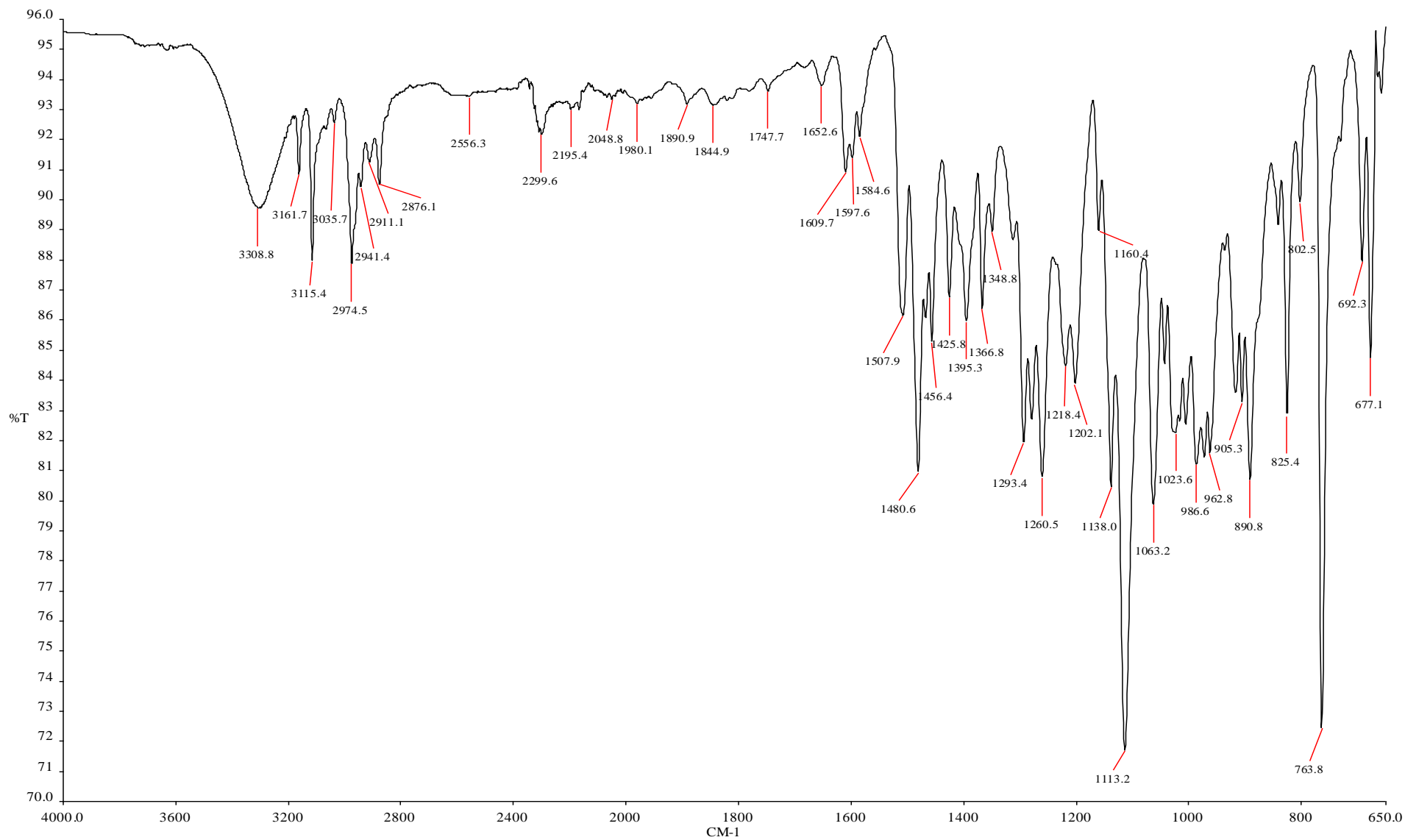

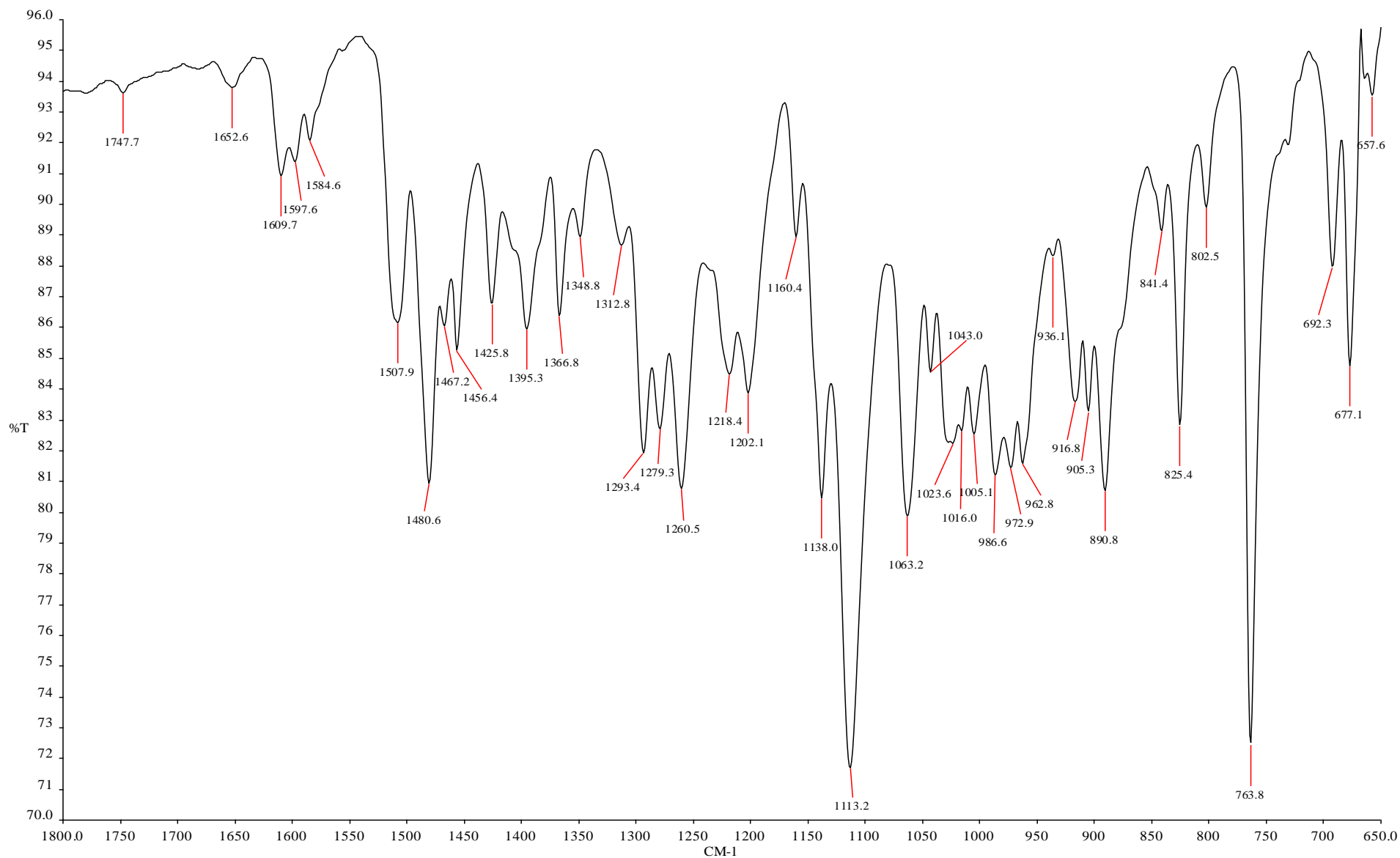

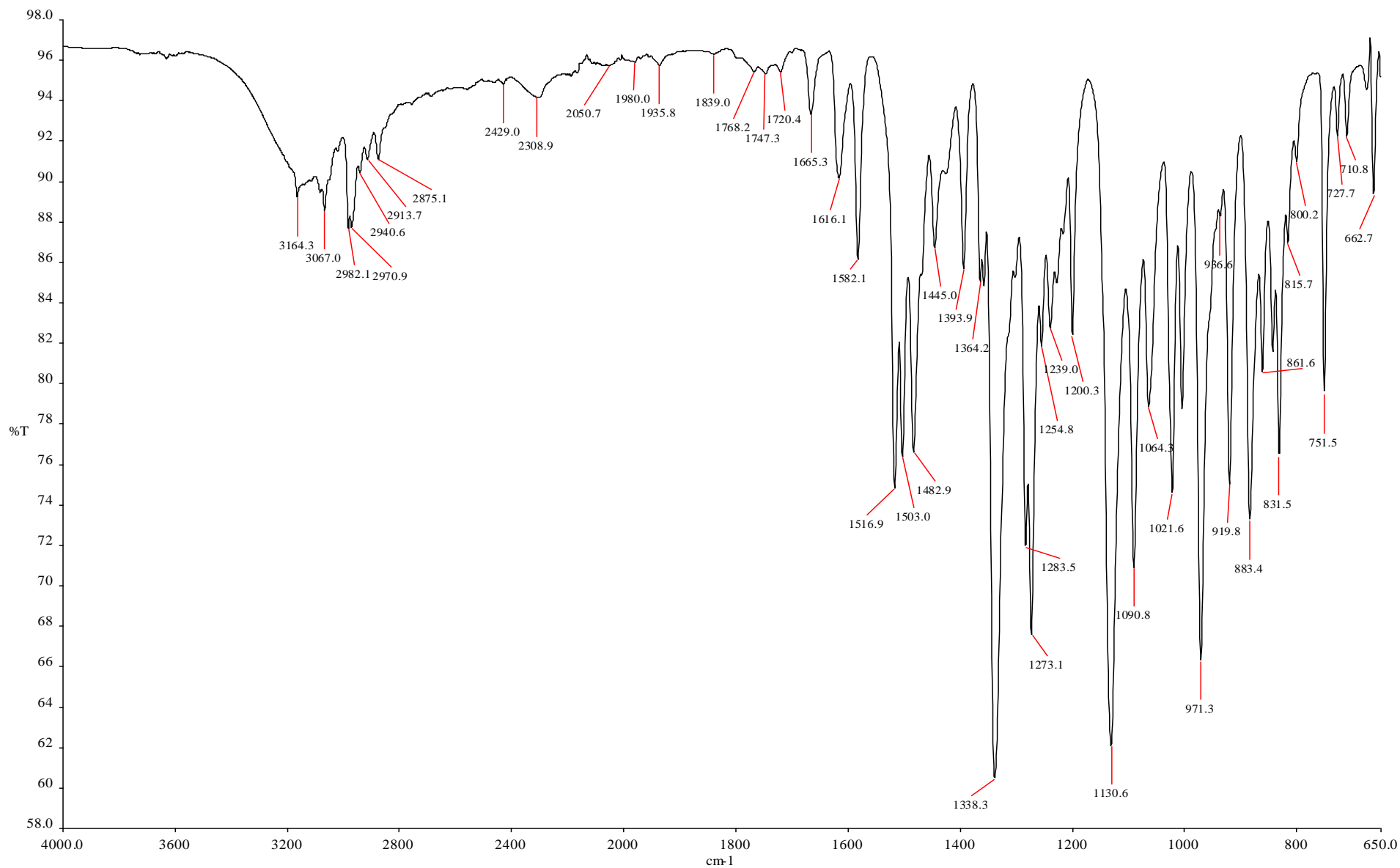

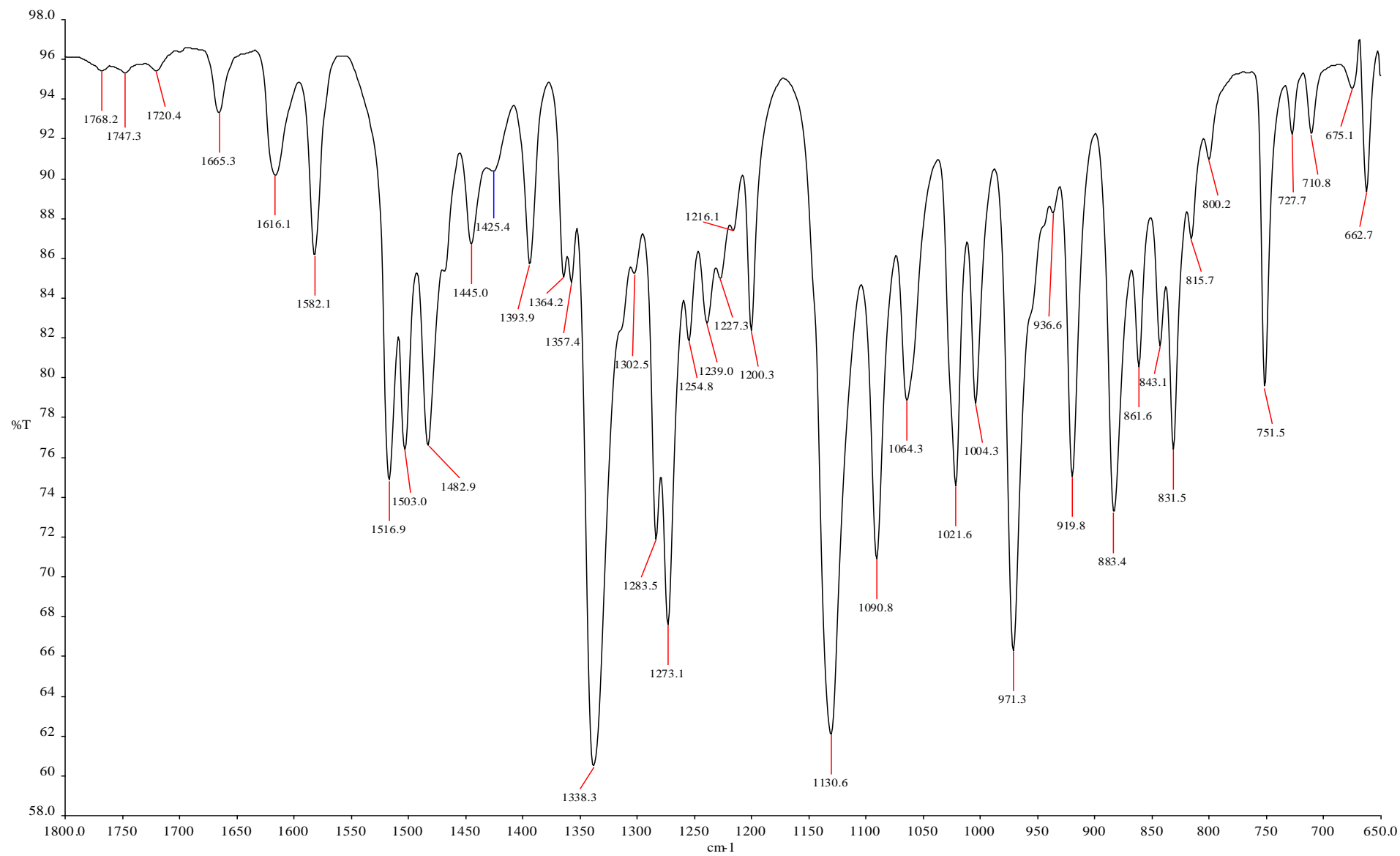

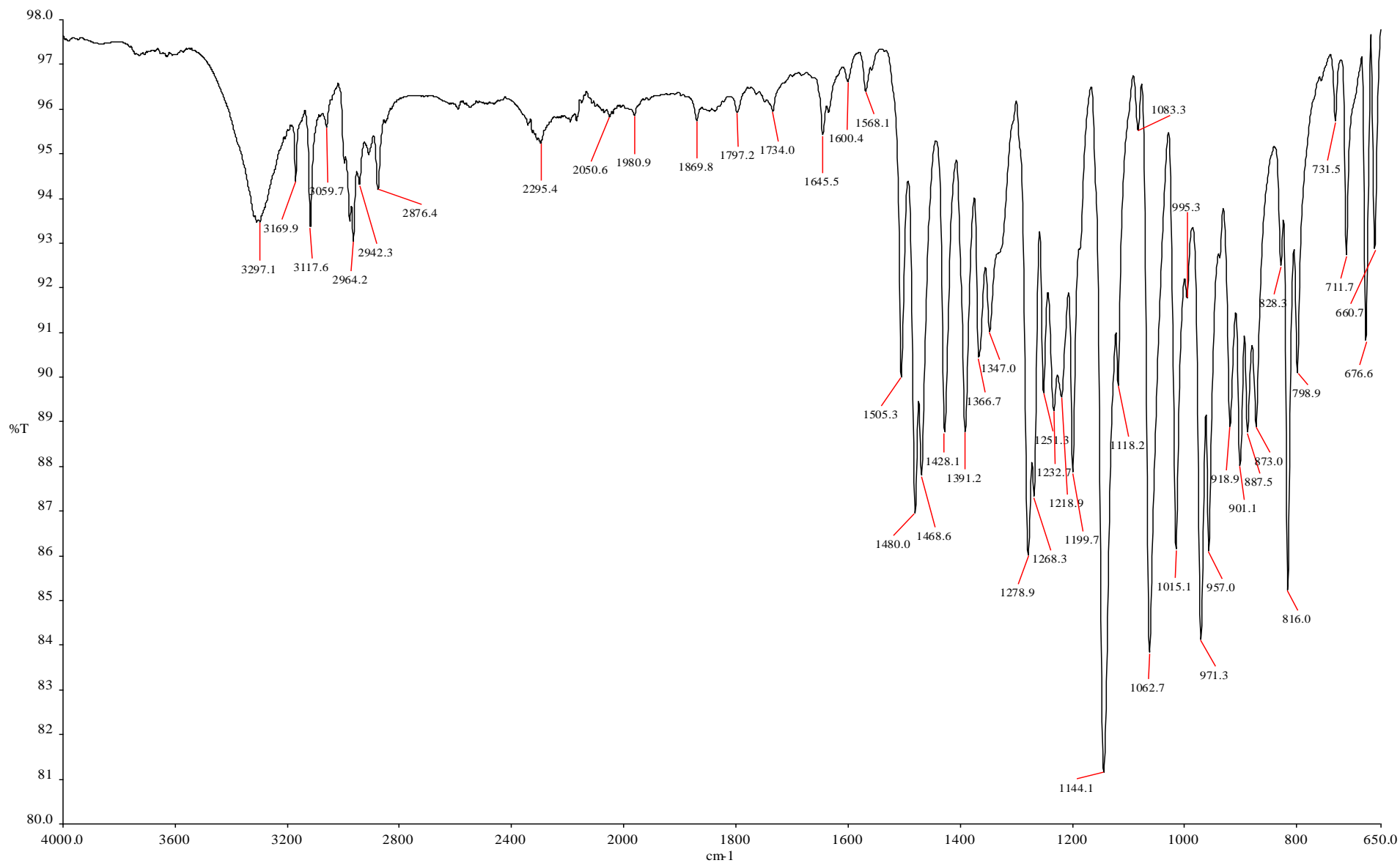

3m

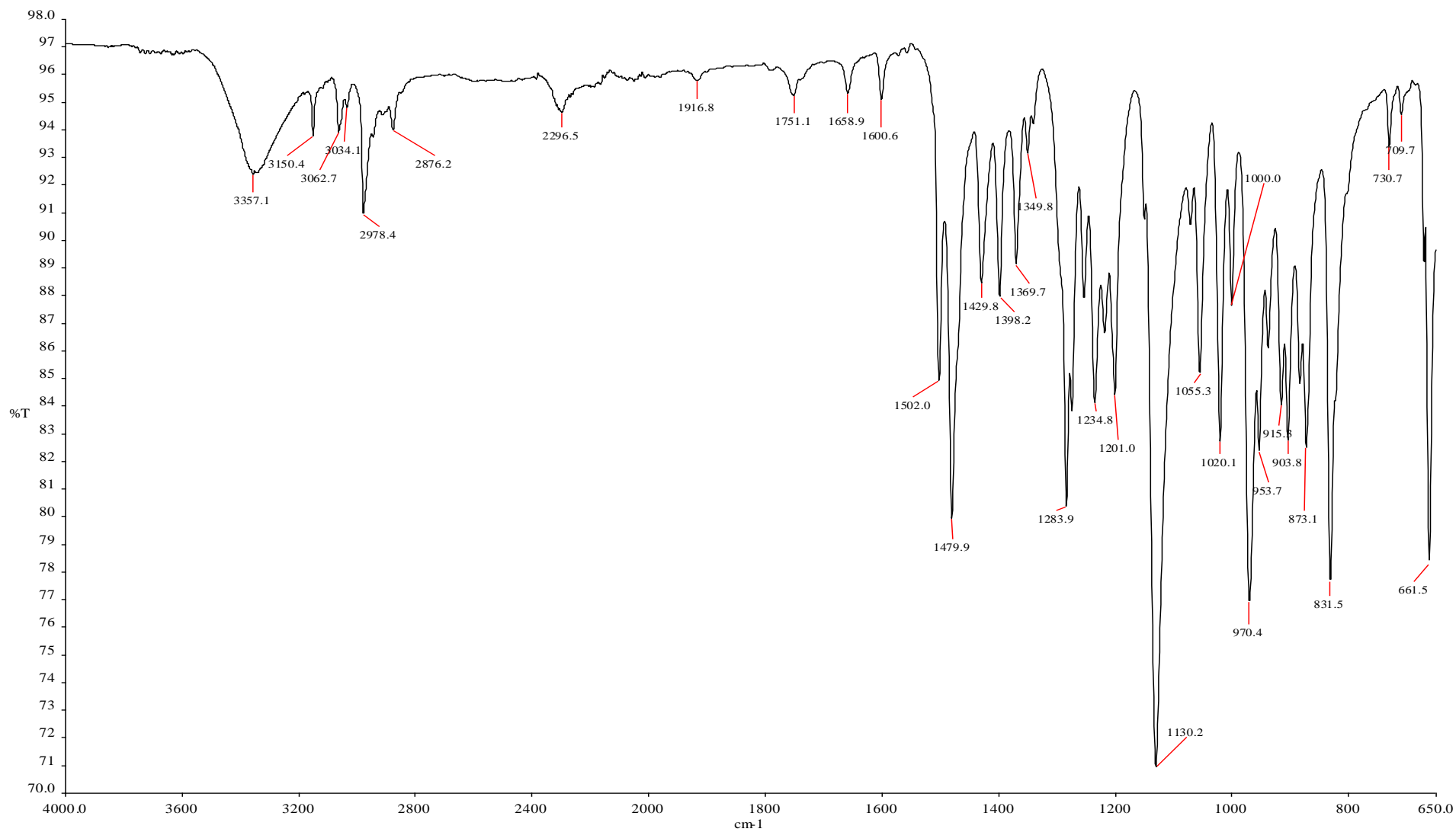

3n

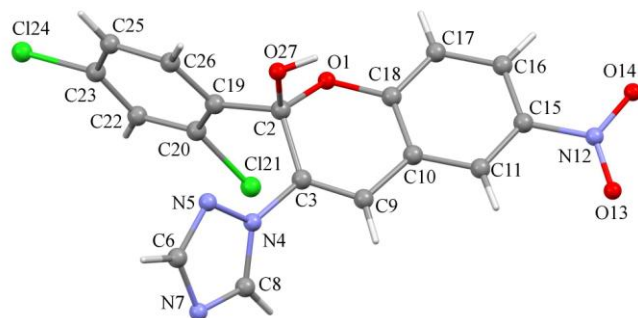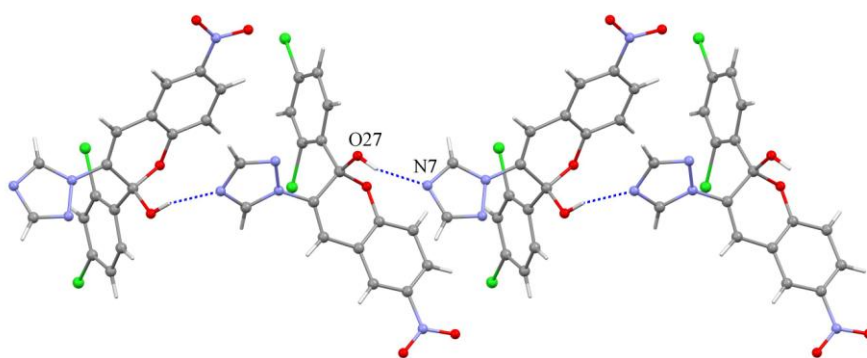

Supplement: Supplementary file 1 [file molecules-26-04304-s001.zip › molecules-1290317-supplementary.pdf]
